# Monosubstituted Acetophenone Thiosemicarbazones as Potent Inhibitors of Tyrosinase: Synthesis, Inhibitory Studies, and Molecular Docking

**DOI:** 10.3390/ph14010074

**Published:** 2021-01-18

**Authors:** Katarzyna Hałdys, Waldemar Goldeman, Natalia Anger-Góra, Joanna Rossowska, Rafał Latajka

**Affiliations:** 1 Department of Bioorganic Chemistry, Wrocław University of Science and Technology, 50-370 Wrocław, Poland; 2 Department of Organic and Medicinal Chemistry, Wrocław University of Science and Technology, 50-370 Wrocław, Poland; waldemar.goldeman@pwr.edu.pl; 3 Ludwik Hirszfeld Institute of Immunology and Experimental Therapy, Polish Academy of Science, 53-114 Wrocław, Poland; natalia.anger@hirszfeld.pl (N.A.-G.); joanna.rossowska@hirszfeld.pl (J.R.)

**Keywords:** tyrosinase, thiosemicarbazones, kinetic studies, structure–activity relationship (SAR), molecular docking, melanogenesis

## Abstract

A set of 12 monosubstituted acetophenone thiosemicarbazone derivatives (TSCs) were synthesized and their inhibitory properties toward tyrosinase activity were tested. Moreover, their ability to inhibit melanogenesis in the B16F10 murine melanoma cell line was studied. In order to investigate the nature of interactions between the enzyme and the inhibitors, molecular docking to the active site was performed. TSCs 5, 6, 8, and 9 revealed a half maximal inhibitory concentration (IC_50_) below 1 µM. Compound 6 turned out to be the most potent tyrosinase inhibitor. All investigated compounds showed reversible inhibition of competitive or mixed type. The *para*-substituted TSCs had higher affinity for the enzyme as compared to their *ortho*- and *meta*-analogues. All investigated compounds inhibited melanin production in B16F10 cells at the micromolar level. Molecular docking showed that the sulfur atom of the thiourea moiety penetrates the active site and interacts with copper ions. The above outcomes might be helpful in the design of new tyrosinase inhibitors in the food and cosmetic industries.

## 1. Introduction

Tyrosinase belongs to the oxidoreductase family (EC 1.14.18.1). There are two copper atoms in its active site which are important for catalytic activity [1]. Tyrosinase can catalyze two different reactions. The first is oxidation of monophenols to ortho-quinones and the second is oxidation of *ortho*-diphenols (catechols) to *ortho*-quinones [2].

Melanins are pigments widely distributed in nature. They derive from dopaquinone, which is formed from l-tyrosine in a reaction catalyzed by tyrosinase in the pathway called melanogenesis. Melanins are responsible for skin and hair color in mammals. They also absorb ultraviolet radiation, protecting the skin from damage. However, excess of pigment accumulated in the skin leads to numerous disorders, e.g., freckles, melasma, age spots, or malignant melanoma [3,4].

Tyrosinase is also responsible for the formation of dark pigment (enzymatic browning) in plant-derived foods. This process affects the shelf life of fresh products, decreasing their sensory properties [5].

There are several tyrosinase inhibitors of both natural and synthetic origin described in the literature [6,7,8]. Unfortunately, most of them are toxic or unstable; thus, they cannot be used orally or applied on the skin. Hydroquinone and kojic acid, known components of antipigmentation cosmetics, have been reported to be unsafe in concentrations required for a skin-lightening effect [9,10]. Therefore, there is a need ti search for and characterize new, safe, and effective tyrosinase inhibitors.

Thiosemicarbazones are an interesting group of compounds. They are known for their numerous pharmacological and biological properties as effective antimicrobial [11], antimalarial [12], and anticancer [13] agents.

Tyrosinase possesses two copper ions in its active site, which makes it a potential target for thiosemicarbazones that might chelate ions via the thiourea moiety [14]. Indeed, thiosemicarbazones have gained interest as tyrosinase inhibitors, providing a half maximal inhibitory concentration (IC_50_) on a micromolar level [15,16,17,18,19,20,21,22,23].

In our previous papers [20,21], we reported that acetophenone thiosemicarbazones are more potent tyrosinase inhibitors than their benzaldehyde analogues. Halogenated acetophenone thiosemicarbazones revealed very good inhibitory properties, and some of them showed quite low toxicity toward the B16F10 cell line [21].

In the present work, we discuss the inhibitory effect of a series of monosubstituted (–OH, –NH_2_, –OCH_3_, –NO_2_) acetophenone thiosemicarbazones on the diphenolase activity of tyrosinase. Twelve compounds were synthesized. The kinetics of the interaction between the inhibitors and tyrosinase, the effectiveness of the studied compounds in the inhibition of melanin production by murine melanoma B16F10 cells, and their cytotoxic activity toward B16F10 cells were investigated. The nature of tyrosinase–inhibitor interactions was explained on the basis of molecular docking of the structures to the active site of the enzyme.

## 2. Results

### 2.1. Chemistry

**Figure 1:**
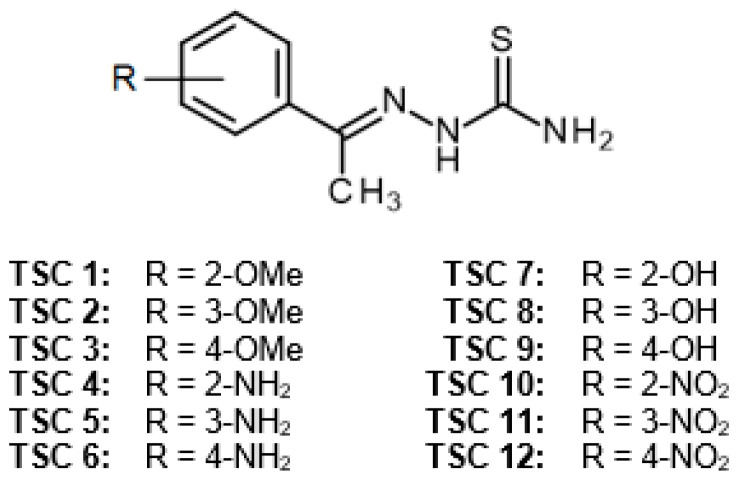
Chemical structures of tested thiosemicarbazone derivatives (TSCs).

The chemical structures of all investigated compounds are shown in the Figure 1.

**Scheme 1:**
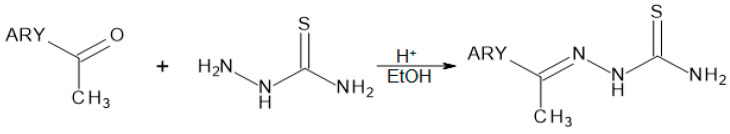
Reaction scheme according to ^1^H-NMR spectroscopy and compared with literature data; in the case of the new TSCs, ^13^C-NMR and HRMS were also performed (Figures S1–S40, Supplementary Materials).

The thiosemicarbazones TSCs 1–12 were prepared via the reaction of the appropriate substituted acetophenone with thiosemicarbazide in the presence of *p*-toluenesulfonic acid (Scheme 1) using a previously reported procedure [20,21].

Most of the synthesized TSCs were obtained as single *E*-isomers, with the exception of the *ortho*-methoxy (TSC 1), *ortho*-amino (TSC 4), and *ortho*-hydroxy (TSC 7) derivatives which were obtained as a mixture of *E*- and *Z*-isomers in the molar ratio 80:20, 90:10, and 92:8, respectively. The configuration of the obtained TSCs was distinguished on the basis of the analysis of the ^1^H-NMR spectra [24,25,26].

The signal of the N^2^H proton (C=N–N*H*–) of the *E*-isomer of thiosemicarbazones of the aryl alkyl ketones appears in the range of ~10.2–10.6 ppm [15,27,28].

**Figure 2:**
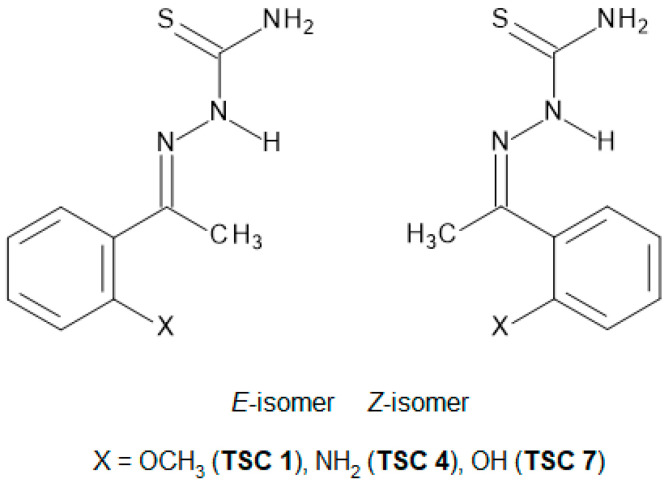
*E*- and *Z*-isomers of *ortho*-substituted thiosemicarbazones (TSC 1, 4, and 7).

The chemical shift value of the N^2^H signal of the major isomer of thiosemicarbazones TSCs 1, 4, and 7 (Figure 2) indicates an *E*-configuration, which suggests that the minor isomer corresponds to the *Z*-isomer. Additionally, the geometry of the C=N double bond of the main isomers of the mentioned TSCs was confirmed by two-dimensional (2D) NMR NOESY spectroscopy. In all three cases, a strong NOE interaction of the appropriate N^2^H signals with the protons of methyl group was observed, which allowed assigning the main isomers to *E*-isomers.

### 2.2. Effect of TSCs 1–12 on the Activity of Mushroom Tyrosinase

The effect of tested TSCs 1–12 on the diphenolase activity of the enzyme was shown as the IC_50_. In order to determine the IC_50_, dose–response analysis for all investigated compounds was performed. The plots representing the relationship between relative activity (percent of inhibition) of the enzyme and the inhibitor concentration (logarithm-transformed for a better fit to a sigmoidal shape).

**Figure 3:**
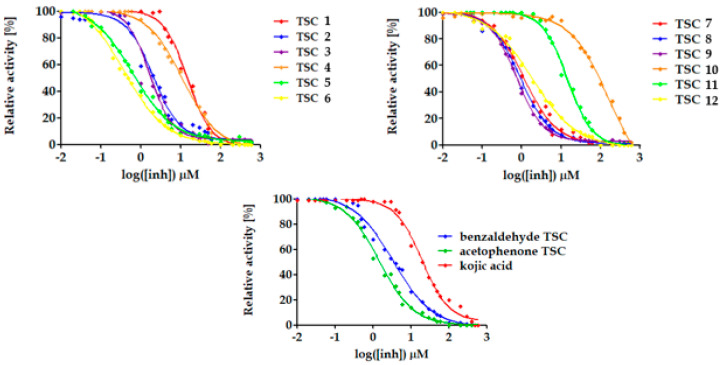
Dose–response curves for determination of half maximal inhibitory concentration (IC_50_) for TSCs 1–12 and control compounds (benzaldehyde TSC, acetophenone TSC, and kojic acid) toward mushroom tyrosinase. The concentration of l-3,4-dihydroxyphenylalanine (l-DOPA) was 1 mM in all the cases. Inhibitor concentration was in the range of 0.01 to 600 µM.

Dose–response curves for TSCs 1–12 are shown in Figure 3. Benzaldehyde TSC, acetophenone TSC, and kojic acid were used as positive controls in this research study. Benzaldehyde TSC and acetophenone TSC were reported as potent tyrosinase inhibitors in our previous papers [20,21]. Kojic acid is the most well known and studied tyrosinase inhibitor. It is often used as a reference compound when new tyrosinase inhibitors are tested [29,30].

All tested compounds showed a dose-dependent manner of inhibition. Dose–response curves allowed determining the IC_50_ values, which are collected in Table 1. All thiosemicarbazones inhibited the diphenolase activity of tyrosinase on a micromolar level of IC_50_, mostly ranging from 0.3 to almost 15 µM. Only TSC 10 showed an IC_50_ over 150 µM. 

The IC_50_ parameter is very convenient, and it is the most often used to show the potency of inhibitors. However, depending on the kinetics of the enzyme–inhibitor interaction, it may depend on the substrate concentration; thus, this parameter is comparable across papers only when the test is taken in the same circumstances [31]. To avoid this drawback, the ratio of the IC_50_ of the investigated inhibitors to the reference compound can be calculated. As kojic acid is the most popular reference compound, the ratio of IC_50_ of kojic acid to the IC_50_ of the tested inhibitors was additionally calculated.

All tested TSCs revealed better inhibitory properties than kojic acid (except for TSC 10). Compounds 5–9 gave better results than acetophenone thiosemicarbazones (unsubstituted analogue of tested compounds).

TSCs 5, 6, 8, and 9 achieved IC_50_ values lower than 1 µM and can be considered as some of the most potent tyrosinase inhibitors described in the literature. They are 20-fold stronger inhibitors than kojic acid. TSC 6 achieving an IC_50_ of 0.34 µM was the most potent one among all those studied in this research.

### 2.3. Kinetic Studies

The kinetics of the interaction between TSCs 1–12 and mushroom tyrosinase were determined. Kojic acid was used as a positive control. The inhibition type, mechanism, and inhibitory constants (K_i_ and K_is_) were determined using graphical methods as described in our previous papers [20,21] and by Copeland [31]. The results are shown in Table 2. 

The inhibition type was determined through creation of the Lineweaver–Burk plot (double reciprocal plot), which enables easier analysis of the Michaelis–Menten plot. Compounds 3, 6, 9, and 11 inhibited tyrosinase competitively, i.e., they competed with the tyrosinase substrates for the enzyme active site. TSCs 1, 2, 4, 5, 7, 8, 10, and 11 were mixed-type inhibitors, with an affinity for both the free enzyme and the enzyme–substrate complex. In all the cases of mixed inhibitors, K_i_ values were lower than K_is_, which means that the inhibitor affinity for the free enzyme was higher than that for the enzyme–substrate complex.

Inhibition constants K_i_ were determined from the secondary plots of *k*_m_ (Michaelis constant) versus the inhibitor concentration for competitive inhibition and by the secondary plots of the slope of the lines from the Lineweaver–Burk plot as a function of the inhibitor concentration for mixed-type inhibition. Inhibition constant K_is_ was determined from the secondary plots of 1/V_max_ as a function of the inhibitor concentration for mixed-type inhibition. All investigated compounds achieved a lower K_i_ value than kojic acid. TSCs 3, 5–9, and 11 revealed a K_i_ lower than 1 µM. It is worth mentioning that *para*-substituted compounds showed a shared inhibition type. They were all competitive inhibitors, whereas *meta*- and *ortho*-substituted compounds were mixed inhibitors.

**Figure 4:**
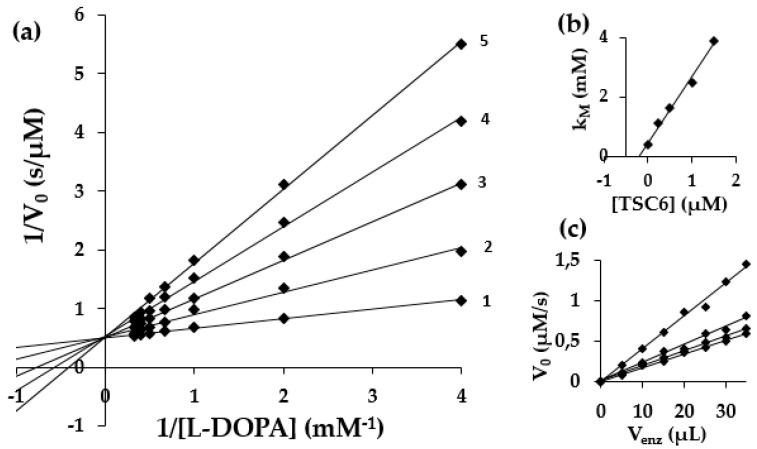
Lineweaver–Burk plots for inhibition of TSC 6 (**a**) on mushroom tyrosinase with l-DOPA as substrate. The concentrations of TSC 6 for curves 1–5 were 0.0, 0.1, 0.2, 0.3, and 0.4 µM, respectively. (**b**) Secondary plot of *k_m_* versus inhibitor concentrations for determination of K_i_ for competitive inhibition. (**c**) Plot to determine the inhibitory mechanism.

Results for the inhibitor affinity for the enzyme expressed in terms of IC_50_ and K_i_ were mostly consistent. Compound 6 showed the lowest values for both these parameters. An exemplary set of plots for the determination of kinetic parameters is shown in Figure 4. 

The inhibition mechanism for the investigated compounds was also tested. All studied thiosemicarbazones revealed a reversible manner of inhibition. As shown in Figure 4c, the plot representing the remaining tyrosinase activity versus the enzyme amount at different inhibitor concentrations for TSC 6 gave a family of straight lines which all passed through the origin of the coordinate system. The presence of the inhibitor reduced the enzyme activity for the same amount of the enzyme.

### 2.4. Molecular Docking

Molecular docking is a helpful tool for an explanation of the interactions between investigated thiosemicarbazones and the enzyme. All TSCs were docked to the active site of mushroom tyrosinase (Protein Data Bank (PDB): 2Y9X) [32]. As a result of the docking process, for each inhibitor, three possible orientations of the docked molecules were obtained. The GoldScore fitness function [33] evaluated the probability of the orientation of the TSCs in the active site. The docked molecules obtaining the highest GoldScore (Table S1, Supplementary Materials) were chosen for the visualization of their orientation in the active site.

**Figure 5:**
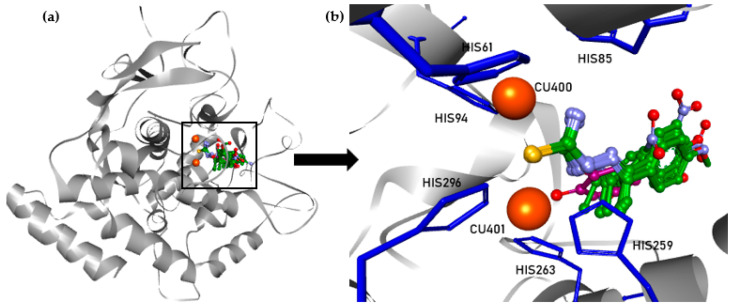
Orientation of TSCs 1–12 and tropolone in chain A of tyrosinase (**a**) and the active site of tyrosinase (**b**). Copper ions are represented as orange spheres; histidine residues are represented as blue sticks; inhibitors are represented by balls and sticks (green—carbon of TSCs; purple—carbon of tropolone, yellow—sulfur; light blue—nitrogen; red—oxygen, hydrogens are not presented).

All molecules are shown in one figure along with the reference ligand (tropolone, the native ligand from the crystal structure of the protein used in the molecular modeling). Indeed, all 12 inhibitors fell into one cluster as along with the native ligand, as clearly visible in Figure 5.

In all cases, the adjustment of TSCs 1–12 in the active site of the enzyme showed the same manner. Inhibitors penetrated the active pocket with the thiourea moiety. For all inhibitors, the sulfur atom of thiourea was located between the two catalytic copper ions of the active site.

**Figure 6:**
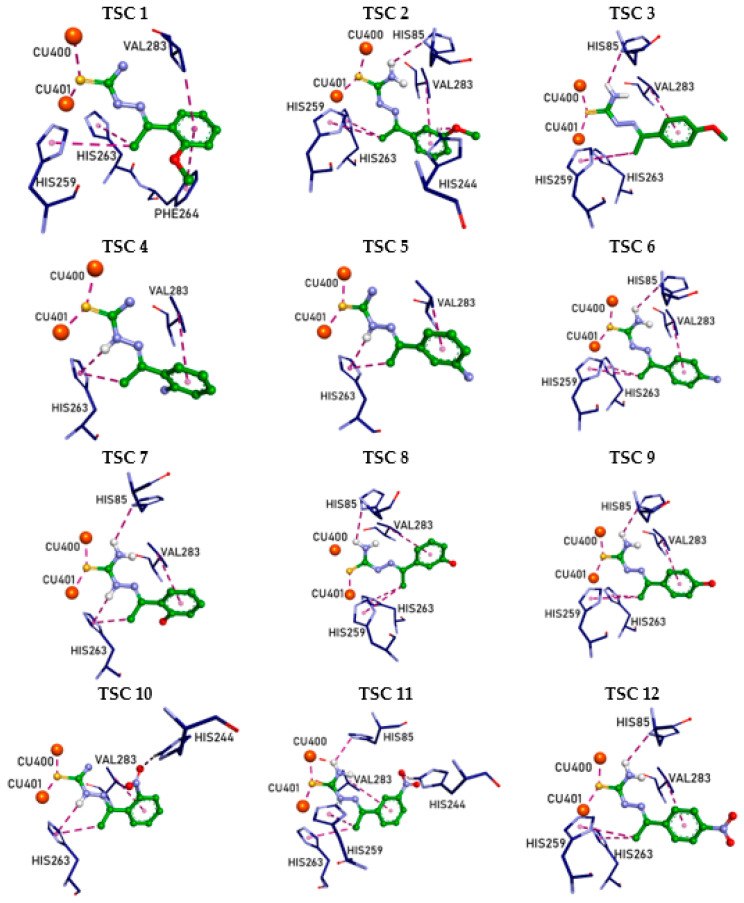
Interactions of TSCs 1–12 in the active site of tyrosinase visualized in BIOVIA Discovery StudioVisualizer [34]. Copper ions are represented as orange spheres; amino-acid residues are represented as sticks; inhibitors are represented by balls and sticks (green—carbon of TSCs; dark blue—carbon of amino acids, yellow—sulfur; light blue—nitrogen; red—oxygen; hydrogens are not presented if they did not participate in the interactions; purple lines—interactions).

As all inhibitors were oriented in the same mode in the active site, there were similar types of interactions between them and residues of the enzyme (Figure 6).

All compounds interacted with copper ions (Cu400 and Cu401) in the active site.

For most of the investigated compounds, there was no interaction with the group located in the aromatic ring of the inhibitor. Hydroxyl and amino groups did not interact with the active site of the enzyme. Only TSCs 2, 10, and 11 showed an interaction with one of the hydrogens of the imidazole ring of His244 via a hydrogen bond with the oxygen of the methoxy (TSC 2) or nitro group (TSCs 10 and 11) of the inhibitor. Interestingly, TSCs with a *para*-substituted aromatic ring did not reveal any interaction with the enzyme, in any case.

TSCs 2, 3, 6–9, 11, and 12 were stabilized by a hydrogen bond formed between one of the hydrogens of the terminal amino group in the thiosemicarbazide moiety and the nitrogen in the imidazole ring of His85. TSCs 4, 5, 7, and 10 formed another hydrogen bond between the hydrogen of the hydrazine group and the imidazole ring (π donor) of His263. 

All structures were additionally stabilized by multiple hydrophobic interactions: π–alkyl interactions of the CH_3_ group of the inhibitor with the imidazole of His259 and His263, as well as π–alkyl or π–sigma interactions between the aromatic ring of the inhibitors and Val283.

A detailed description of all interactions between TSCs 1–12 and the active site of tyrosinase, along with the length of these interactions, can be found in the Supplementary Materials (Section 1).

### 2.5. Effectiveness of TSCs in Melanogenesis Inhibition

In order to investigate the inhibitory effect of the studied TSCs 1–12 on melanogenesis, B16F10 murine melanoma cells were stimulated with α-melanocyte-stimulating hormone (α-MSH). In the presence of α-MSH, the cells were treated with the inhibitors after 24 h. Then, the cells were incubated with TSCs at various concentrations (1–100 μM) for 48 h. Kojic acid was used as a positive control, and dimethyl sulfoxide (DMSO) was used as a solvent control. Melanin content was measured spectrophotometrically at 405 nm. B16F10 cell viability was also estimated in the presence of the tested inhibitors, benzaldehyde thiosemicarbazone, kojic acid, and DMSO using the 3-(4,5-dimethylthiazol-2-yl)-2,5-diphenyltetrazolium bromide (MTT) colorimetric assay.

The results of dose–response measurements (IC_50_), along with confidence intervals, for the inhibition of melanin production and cell proliferation are shown in Table 3. 

Melanin production in B16F10 cells was inhibited by all tested TSCs. All of the investigated compounds revealed an IC_50_ at the micromolar level. Because of problems with the colorimetric assays, compounds 4 and 12 were not taken into consideration for further analysis. Almost all inhibitors showed a strong potency of melanogenesis inhibition (low IC_50_ values in comparison with positive control—kojic acid, which achieved an IC_50_ of 114.1 µM). The exception was TSC 6 which achieved an IC_50_ of 139.4 µM for the inhibition of melanin production.

These results were verified with the effect of tested inhibitors on the proliferation of B16F10 cells (see Table 3). Low melanin production might have been caused by high toxicity (low IC_50_ for inhibition of cell proliferation) of the inhibitors which, in turn, could have led to the inhibition of cell proliferation.

TSCs 2, 3, 7, and 9 influenced the melanin production at the relatively low concentration rate (IC_50_ below 30 µM); however, in the case of compounds 2 and 3, this might have been caused by the toxicity of the compounds toward the cells.

**Figure 7:**
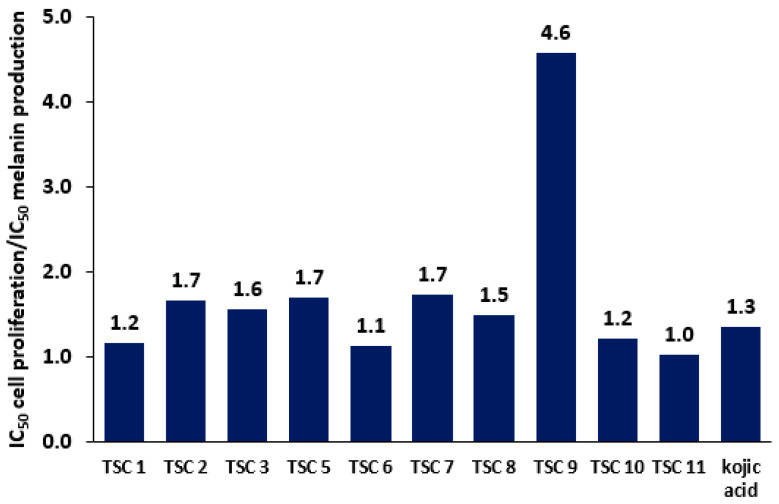
Ratio of IC_50_ for the inhibition of B16F10 cell proliferation to IC_50_ for the inhibition of melanin production.

In order to visualize the potency of TSCs in inhibiting melanin production with regard to their toxicity, the bar graph representing the ratio of the IC_50_ for the inhibition of B16F10 cell proliferation to the IC_50_ for the inhibition of melanin production was created for TSCs and kojic acid (Figure 7).

A higher bar denotes a better ratio of melanogenesis inhibition to toxicity. For compounds 1–3, 5–8, 10, and 11, the value of this ratio was between 1 and 2. The compound leading to the highest bar was compound 9, which means it can be considered as a potent nontoxic inhibitor of melanogenesis in B16F10 cells.

## 3. Discussion

In the frame of this study, 12 TSCs were synthesized and their inhibitory activity toward mushroom tyrosinase was examined. Moreover, the molecular docking of these compounds to the active site of the enzyme was performed, and their ability to inhibit melanogenesis and cell proliferation in B16F10 cells was tested.

Some general points regarding the structure–activity relationship of thiosemicarbazones can be concluded on the basis of the results of the dose–response analysis and obtained IC_50_ values.

According to the observed great impact of the methyl group, instead of hydrogen, on the biological activity of the thiosemicarbazones toward tyrosinase in our previous papers [20,21], it was decided to test a group of acetophenone thiosemicarbazone derivatives possessing methoxy, amino, hydroxyl, and nitro substituents on the phenyl ring at the *ortho*-, *meta-,* and *para*-positions. The structure–activity relationship is very similar to compounds substituted with halogen atoms (Br, Cl, F) concerning the position of substitution on the phenyl ring [21]. In all cases, the *ortho*-position provided the highest IC_50_ values as compared to the *meta*- and *para*-substitutions. For compounds with amino (TSC 1), methoxy (TSC 4), and hydroxyl (TSC 7) groups, the values of IC_50_ were 11.3, 12.2, and 1.2 µM, respectively. A nitro group at the *ortho*-position (TSC 10) increased the IC_50_ to 154.5 µM. There were no significant differences in IC_50_ values between the *meta*- and *para*-position for compounds possessing hydroxyl, amino, and methoxy groups. However, in all abovementioned cases, the *para*-substitution was slightly more beneficial. For compounds with a nitro group, the inhibitory activity visibly decreased from the *para*- to *ortho*-position. In the case of these compounds, the type of group substituted seemed to have a greater impact on the biological activity than in the case of halogen substituents [21]. The methoxy substituent provided IC_50_ values of 1.8 and 1.9 µM for TSCs 3 (*para*) and 2 (*meta*), respectively. The hydroxyl and amino groups at the *meta*- and *para*-position achieved an IC_50_ below 1 µM, and TSC 6 (4-aminoacetophenone thiosemicarbazone) possessed the best inhibitory properties among all investigated thiosemicarbazones in this study, reaching the lowest IC_50_ value of 0.34 µM.

In general, it is reported in the literature that thiosemicarbazones are reversible inhibitors of mushroom tyrosinase, revealing all types of inhibition (competitive, noncompetitive, uncompetitive, and mixed) depending on the substituents. This indicates that TSCs have an affinity for both the free enzyme and the substrate–enzyme complex. In the case of our studies, there was a certain relationship between the type of inhibition and the structure. The *para*-substituted acetophenone thiosemicarbazones were competitive inhibitors, whereas the *meta*- and *ortho*-substituted compounds showed a mixed type of inhibition. This is coherent with our results reported for halogenated acetophenone TSCs [21].

Molecular docking studies showed that the investigated TSCs penetrated the active site of tyrosinase via the thiourea moiety. All molecules fell into the same cluster; thus, their orientation in the active site did not explain the changes in the activity of particular TSCs. However, it is clear that the thiourea moiety is essential in the inhibition process, as in all cases for all compounds, the sulfur atom interacted with copper ions in the active site. Moreover, tropolone (native ligand of the crystal structure) occupied the same position as all TSCs but did not chelate any of the catalytic copper ions [32]. Hydrophobic interactions seem to substantially contribute to the inhibition process, which could have been predicted from the fact that tyrosinase’s active site possesses multiple hydrophobic amino-acid residues [35]. Another important type of interaction stabilizing the structures in the active site was the hydrogen bond. Monosubstituted acetophenone thiosemicarbazone derivatives seemed to interact in a very similar way with the active site of the enzyme independently of the type of substituent on the aromatic ring. The results of the current research are similar to our results reported [21] for halogenated acetophenone thiosemicarbazone derivatives. These insights might be helpful in designing and developing novel tyrosinase inhibitors.

All investigated thiosemicarbazones inhibited melanogenesis in B16F10 cells at the micromolar level. Surprisingly, the substitution of the aromatic ring of the inhibitors with groups analyzed in this paper (methoxy, hydroxy, amino, and nitro) provided higher IC_50_ values for both the inhibition of melanin production and the inhibition of cell proliferation as compared with halogen substituents [21]. Moreover, the ratio of the IC_50_ for the inhibition of B16F10 cell proliferation to the IC_50_ for the inhibition of melanin production was much lower than for halogenated analogues. It seems that, despite a similar affinity for tyrosinase, the investigated compounds showed a lesser inhibitory effect on the whole melanogenesis process. Interestingly, TSC 6, which showed the best inhibitory properties toward tyrosinase, turned out to be very weak inhibitor of melanogenesis.

Almost all studies related to the inhibition of tyrosinase were performed using mushroom tyrosinase. In fact, the enzyme from *Agaricus bisporus* is easily available in purified form, it is very active, and the enzymatic assay is convenient. Moreover, it is highly homologous with the mammalian enzyme, which makes it a common model for studies on melanogenesis in mammals [36,37,38]. Unfortunately, there are limitations to such an experimental approach. In spite of the similarities between human and mushroom tyrosinases, such as the conserved active site, there are also numerous structural differences which might influence the inhibition process. Human tyrosinase is a monomer undergoing glycosylation during its maturation, whereas the mushroom enzyme is a tetramer. The human enzyme is membrane-bound in contrast with the mushroom one which is located in the cytosol [29]. Some studies reported that the affinity of inhibitors for mammalian tyrosinase is lower than that for the mushroom one [39]. There is no research on thiosemicarbazones as human or mammalian tyrosinase inhibitors. Moreover, there is no crystal structure of human tyrosinase reported. Further investigations should also be made for the human enzyme to confirm the strong potency of thiosemicarbazones as tyrosinase inhibitors.

Results of our studies show that introducing simple groups (methoxy, hydroxy, amino, and nitro) to the thiosemicarbazone structure can improve its inhibitory properties toward tyrosinase, and this might be useful in the design of new skin-lightening agents or anti-browning compounds.

## 4. Materials and Methods

### 4.1. Chemistry

General information: thiosemicarbazide, *p*-toluenesulfonic acid monohydrate, and all used substituted acetophenones were purchased from Sigma-Aldrich (Taufkirchen, Germany), while 96% ethanol and methanol were obtained from a local supplier (Avantor Performance Materials Poland S.A.). High-resolution mass spectra were obtained on an LCT Premier XE Waters apparatus, in positive electrospray ionization (ESI+) mode. The spectra were recorded on 600 MHz Bruker Avance and Jeol ECZ 400S spectrometers in DMSO-d_6_ as the solvent. Chemical shifts (δ) were reported in ppm relative to solvent signals (2.50 ppm and 39.52 ppm in ^1^H- and ^13^C-NMR spectra, respectively). The following abbreviations for multiplicity were used: s = singlet, d = doublet, t = triplet, q = quartet, quint = quintet, sext = sextet, hept = heptet, m = multiplet, and br = broad.

#### General Procedure for the Synthesis of TSCs 1–12

The mixture of appropriate substituted acetophenone (0.016 mol), thiosemicarbazide (1.8 g, 0.02 mol), and *p*-toluenesulfonic acid monohydrate (3.0 g, 0.016 mol) in 96% ethanol (50 mL) was refluxed for about 8 h, cooled to room temperature, and added in few portions to vigorously stirred 10% sodium bicarbonate solution (250 mL). Stirring was continued for about 30 min, and the resulting solid was filtered, washed intensively with water (2 × 50 mL, 10 × 20 mL), and dried in air giving crude TSCs. *Analytical samples were obtained* by crystallization from 96% ethanol (TSCs 2–4, TSC 7 and TSC 9) or methanol (TSC 1, TSCs 5–6, TSC 8, and TSCs 10–12).

Mixture of (*E*)- and (*Z*)-2’-methoxyacetophenone thiosemicarbazone (in ca. 80:20 molar ratio, according to ^1^H-NMR) (TSC 1): yield 80%; ^1^H-NMR (600 MHz, DMSO-d_6_) δ: 2.20 (s, C*H_3_*, *Z*-isomer) and 2.23 (s, C*H_3_*, *E*-isomer) (total integration 3H), 3.817 (s, OC*H_3_*, *E*-isomer) and 3.82 (s, OC*H_3_*, *Z*-isomer) (total integration 3H), 6.96 (dt, J = 0.6 Hz, J = 7.2 Hz, Ar*H*, *E*-isomer) and 7.11 (dt, J = 1.2 Hz, J = 7.2 Hz, Ar*H*, Z-isomer) (total integration 1H), 7.06 (d, J = 8.4 Hz, *E*-isomer) and 7.21 (d, J = 7.8 Hz, *Z*-isomer) (total integration 1H), 7.23 (d, J = 7.8 Hz, *Z*-isomer) and 7.42 (dd, J = 1.8 Hz, J = 7.8 Hz, *E*-isomer) (total integration 1H), 7.38 (dt, J = 1.8 Hz, J = 8.4 Hz, Ar*H*, *E*-isomer) and 7.50 (dt, J = 1.8 Hz, J = 7.8 Hz, Ar*H*, *Z*-isomer) (total integration 1H), 7.64 (bs, *NH, E*-isomer) and 7.89 (bs, *NH, Z*-isomer) (total integration 1H), 8.19 (bs, N*H*, *E*-isomer) and 8.24 (bs, *NH*, *Z*-isomer) (total integration 1H), 8.36 (bs, N*H*, Z-isomer) and 10.18 (s, N*H*, *E*-isomer) (total integration 1H). ^13^C-NMR (100 MHz, DMSO-d_6_) δ: major, *E*-isomer: 18.68, 56.07, 112.11, 120.83, 129.09, 130.06, 130.83, 150.97, 157.77, 179.46; minor *Z*-isomer: 24.64, 56.28, 112.82, 121.82, 122.75, 128.50, 131.86, 148.84, 155.68, 179.46. HRMS (ESI+): *m*/*z* calculated for C_10_H_14_N_3_OS (M + H)^+^ 224.0858, found 224.0856.

(*E*)-3’-Methoxyacetophenone thiosemicarbazone (TSC 2): yield 97%; ^1^H-NMR (600 MHz, DMSO-d_6_) δ: 2.30 (s, 3H, C*H_3_*), 3.81 (s, 3H, C*H_3_O*), 6.97 (dd, 1H, J = 3.0 Hz, J = 8.4 Hz, Ar*H*), 7.31 (t, 1H, J = 7.8 Hz, Ar*H*), 7.44–7.49 (m, 2H, Ar*H*), 7.95 (bs, 1H, N*H*), 8.29 (bs, 1H, N*H*), 10.20 (s, 1H, N*H*). NMR data are in agreement with those reported in the literature [40,41]. 

(*E*)-4’-Methoxyacetophenone thiosemicarbazone (TSC 3): yield 91%; ^1^H-NMR (600 MHz, DMSO-d_6_) δ: 2.28 (s, 3H, C*H_3_*), 3.80 (s, 3H, C*H_3_O*), 6.93 (d, 2H, J = 8.7 Hz, Ar*H*), 7.86–792 (d + bs, 3H, J = 7.8 Hz, Ar*H* + N*H*), 8.22 (bs, 1H, N*H*), 10.13 (s, 1H, N*H*). NMR data are in agreement with those reported in the literature [27].

Mixture of (*E*)- and (Z)-2’-aminoacetophenone thiosemicarbazone (in ca. 90:10 molar ratio, according to ^1^H-NMR) (TSC 4): yield 91 %; ^1^H-NMR (400 MHz, DMSO-d_6_) δ: 2.18 (s, C*H_3_*, *Z*-isomer) and 2.23 (s, C*H_3_*, *E*-isomer) (total integration 3H), 5.16 (s, NH*_2_*, *Z*-isomer) and 6.00 (bs, NH*_2_*, *E*-isomer) (total integration 2H), 6.50 (t, J = 8.0 Hz, Ar*H*, *E*-isomer) and 6.65 (t, J = 7.6 Hz, Ar*H*, Z-isomer) (total integration 1H), 6.67 (d, J = 8.0 Hz, *E*-isomer) and 6.79 (d, J = 7.6 Hz, *Z*-isomer) (total integration 1H), 7.00 (t, J = 7.2 Hz, *E*-isomer + overlapped signal *Z*-isomer) and 7.13 (dt, J = 1.6 Hz, J = 8.4 Hz, *Z*-isomer) and 7.29 (d, J = 8.0 Hz, *E*-isomer) (total integration 2H), 7.43 (bs, *NH, E*-isomer) and 7.77 (bs, *NH, Z*-isomer) (total integration 1H), 8.13 (bs, N*H*, *E*-isomer) and 8.26 (bs, *NH*, *Z*-isomer) (total integration 1H), 8.61 (bs, N*H*, Z-isomer) and 10.06 (s, N*H*, *E*-isomer) (total integration 1H). ^13^C-NMR (100MHz, DMSO-d_6_) δ: major, *E*-isomer: 16.92 (bs), 115.89 (bs), 116.64 (bs), 120.34 (bs), 130.00 (bs), 147.60 (bs), 153.65 (bs), 179.03 (bs), (one aromatic *C*H carbon signal is missing, probably due to the broadening and overlaps); minor *Z*-isomer: 24.47, 116.72, 117.55, 119.60, 128.08, 130.91, 144.41, 150.51, 178.35. HRMS (ESI+): *m*/*z* calculated for C_9_H_13_N_4_S (M + H)^+^ 209.0861, found 209.0858.

(*E*)-3’-Aminoacetophenone thiosemicarbazone (TSC 5): yield 97%; ^1^H-NMR (600 MHz, DMSO-d_6_) δ: 2.24 (s, 3H, C*H_3_*), 5.06 (bs, 2H, NH_2_), 6.61 (m, 1H, Ar*H*), 7.01–7.06 (m, 2H, Ar*H*), 7.11 (m, 1H, Ar*H*), 7.69 (bs, 1H, N*H*), 8.31 (bs, 1H, N*H*), 10.20 (s, 1H, N*H*). NMR data are in agreement with those reported in the literature [16,42,43].

(*E*)-4’-Aminoacetophenone thiosemicarbazone (TSC 6): yield 94%; ^1^H-NMR (400 MHz, DMSO-d_6_) δ: 2.13 (s, 3H, C*H_3_*), 5.42 (bs, 2H, NH_2_), 6.48 (d, 2H, J = 8.8 Hz, Ar*H*), 7.57 (d, 2H, J = 8.8 Hz, Ar*H*), 7.68 (bs, 1H, N*H*), 8.04 (bs, 1H, N*H*), 9.92 (s, 1H, N*H*). NMR data are in agreement with those reported in the literature [16,43].

Mixture of (*E*)- and (Z)-2’-hydroxyacetophenone thiosemicarbazone (in 92:8 molar ratio, according to ^1^H-NMR) (TSC 7): yield 74 %; ^1^H-NMR (400 MHz, DMSO-d_6_) δ: 2.17 (s, C*H_3_*, *Z*-isomer) and 2.27 (s, C*H_3_*, *E*-isomer) (total integration 3H), 6.81 (t, J = 8.4 Hz, Ar*H*, *E*-isomer) and 6.91 (t, J = 7.6 Hz, Ar*H*, Z-isomer) (total integration 1H), 6.82 (d, J = 8.8 Hz, *E*-isomer) and 6.96 (d, J = 8.4 Hz, *Z*-isomer) (total integration 1H), 7.15 (dd, J = 1.6 Hz, J = 7.6 Hz, *Z*-isomer) and 7.48 (d, J = 8.0 Hz, *E*-isomer) (total integration 1H), 7.20 (dt, J = 1.2 Hz, J = 8.0 Hz, *E*-isomer) and 7.28 (dt, J = 1.6 Hz, J = 8.8 Hz, *Z*-isomer) (total integration 1H), 7.55 (bs, *NH, E*-isomer), 7.81 (bs, N*H*, Z-isomer), 8.13 (bs, *NH, E*-isomer), 8.29 (bs, N*H*, *Z*-isomer), 8.39 (bs, *NH*, *Z*-isomer) and 10.58 (bs, N*H*, *E*-isomer) (total integration 3H), 10.31 (bs, *OH, Z*-isomer) and 12.66 (bs, *OH, E*-isomer) (total integration ~0.6H (due to strong broadening of the signal at 12.66 ppm, observed integration is lower than the theoretical)). ^13^C-NMR (100 MHz, DMSO-d_6_) δ: major, *E*-isomer: 14.60 (bs), 117.44 (bs), 119.18 (bs), 129.06 (bs), 131.12 (bs), 152.68 (bs), 157.79 (bs), 181.04 (bs), (one aromatic *C*H carbon signal is missing, probably due to the broadening and overlaps); minor *Z*-isomer: 24.69, 116.88, 120.41, 121.35, 128.79, 131.63, 149.41, 153.65, 178.22. Note: on ^1^H- and ^13^C-NMR spectra an peaks of residual crystallization solvent (ethanol) are present. HRMS (ESI+): *m*/*z* calculated for C_9_H_12_N_3_OS (M + H)^+^ 210.0701, found 210.0702; Thiosemicarbazone TSC 7 is described in the literature as a single isomer [42,44].

(*E*)-3’-Hydroxyacetophenone thiosemicarbazone (TSC 8): yield 81%; ^1^H-NMR (400 MHz, DMSO-d_6_) of the methanol solvate of TSC 8 (TSC 8•CH_3_OH) δ: 2.21 (s, 3H, C*H_3_*), 2.47 (s, ~3H, C*H_3_*OH), 4.08 (bs, ~1H, CH_3_O*H*), 6.75 (dd, 1H, J = 2.0 Hz, J = 8.0 Hz, Ar*H*), 7.13 (t, 1H, J = 7.6 Hz, Ar*H*), 7.20 (t, 1H, J = 2.0 Hz, Ar*H*), 7.29 (d, 1H, J = 7.6 Hz, Ar*H*), 7.74 (bs, 1H, N*H*), 8.23 (bs, 1H, N*H*), 9.42 (s, 1H, OH), 10.17 (s, 1H, N*H*); ^13^C-NMR (100 MHz, DMSO-d_6_) δ: 14.70, 49.13 (CH_3_OH), 113.80, 116.82, 118.04, 129.75, 139.58, 148.65, 157.76, 179.40. HRMS (ESI+): *m*/*z* calculated for C_9_H_12_N_3_OS (M + H)^+^ 210.0701, found 210.0708; NMR data of the obtained methanol solvate are in good agreement with those reported for the free TSC 8 [42].

(*E*)-4’-Hydroxyacetophenone thiosemicarbazone (TSC 9): yield 93%; ^1^H-NMR (400 MHz, DMSO-d_6_) δ: 2.19 (s, 3H, C*H_3_*), 6.71 (m (AA’BB’ system), 2H, Ar*H*), 7.72 (m (AA’BB’ system), 2H, Ar*H*), 7.77 (bs, 1H, N*H*), 8.13 (bs, 1H, N*H*), 9.69 (s, 1H, OH), 10.03 (bs, 1H, N*H*). NMR data are in agreement with those reported in the literature [42,45,46].

(*E*)-2’-Nitroacetophenone thiosemicarbazone (TSC 10): yield 31%; ^1^H-NMR (400 MHz, DMSO-d_6_) δ: 2.32 (s, 3H, C*H_3_*), 7.28 (bs, 1H, N*H*), 7.59 (m, 1H, Ar*H*), 7.63–7.73 (m, 2H, Ar*H*), 7.92 (d, 1H, J = 7.2 Hz, Ar*H*), 8.28 (bs, 1H, N*H*), 10.36 (s, 1H, N*H*). NMR data are in agreement with those reported in the literature [46].

(*E*)-3’-Nitroacetophenone thiosemicarbazone (TSC 11): yield 97%; ^1^H-NMR (400 MHz, DMSO-d_6_) δ: 2.32 (s, 3H, C*H_3_*), 7.62 (t, 1H, J = 7.6 Hz, Ar*H*), 8.12 (bs, 1H, N*H*), 8.16 (ddd, 1H, J = 0.8 Hz, J = 2.4 Hz, J = 8.4 Hz, Ar*H*), 8.36 (bs, 1H, N*H*), 8.38 (dd, 1H, J = 1.6 Hz, J = 8.4 Hz, Ar*H*), 8.56 (t, 1H, J = 1.6 Hz, Ar*H*), 10.36 (s, 1H, N*H*). NMR data are in agreement with those reported in the literature [43,46].

(*E*)-4’-Nitroacetophenone thiosemicarbazone (TSC 12): yield 94%; ^1^H-NMR (400 MHz, DMSO-d_6_) δ: 2.31 (s, 3H, C*H_3_*), 8.11 (bs, 1H, N*H*), 8.14 (m (AA’BB’ system), 2H, Ar*H*), 8.18 (m (AA’BB’ system), 2H, Ar*H*), 8.43 (bs, 1H, N*H*), 10.42 (s, 1H, N*H*). NMR data are in agreement with those reported in the literature [47].

### 4.2. Enzyme Preparation

The enzyme was isolated from *Agaricus bisporus*, purchased from a local supplier in Wroclaw, and prepared as described in our previous papers [20,21], according to known procedures reported in the literature [48,49,50]. The full description of enzyme preparation is included in the Supplementary Materials (Section 2).

### 4.3. Tyrosinase Enzymatic Assay 

The tyrosinase enzymatic assay was performed as described in our previous papers [20,21] using l-3,4-dihydroxyphenylalanine (l-DOPA) (Sigma-Aldrich, Taufkirchen, Germany) as a substrate. l-DOPA was dissolved in a 0.15 mM phosphoric (V) acid solution to a concentration of 5 mM. All TSCs were dissolved in DMSO to a concentration of 50 mM and then diluted in sodium phosphate buffer (0.1 M, pH 6.8) to test concentrations (DMSO concentrations in final reaction mixtures did not exceeded 1%).

The isolated tyrosinase solution (0.5 mg/mL, 40,000 U/mg) was diluted 10 times in sodium phosphate buffer (0,1 M, pH 6.8). First, 10 µL of the diluted enzyme solution was preincubated with TSC solutions (5 min at 25 °C). The optimal ionic strength and pH for tyrosinase activity were provided by 0.1 M sodium phosphate buffer (pH 6.8).

After the solution of l-DOPA was added, monitoring of the reaction was immediately started by measuring the change in absorbance of the color product (dopachrome) for 10 min (λ = 475 nm, 25 °C) using Molecular Devices *SpectraMax* *Plus* 384 Microplate Reader. The control sample contained the same reagents as the test samples except for the substrate. Kojic acid (Sigma-Aldrich, Taufkirchen, Germany), a positive control, was treated the same way as the other inhibitors. 

### 4.4. Molecular Docking

The structures of TSCs 1–12 were fully optimized in the Gaussian09 set of codes at the B3LYP/6-311g(d,p) level of theory [51] with the polarizable continuum model (PCM) using water as the solvent [52]. The vibrational frequency analysis was performed to confirm that the obtained geometries were true minima on the potential energy surface (determined by the presence of no imaginary frequencies). The crystal structure of tyrosinase from *Agaricus bisporus* was obtained from the Research Collaboratory for Structural Bioinformatics (RCSB) Protein Data Bank (PDB: 2Y9X [32]). The ligand and the water molecules were removed from the structure of the enzyme. The structure was protonated, and charges were added to the protein using the H++ server [53,54,55,56] according to a pH value of 6.8. Molecular docking in the defined active center of the protein was performed using the GOLD Algorithm [33]. The active site was selected as a sphere with the center in the location of the Cu400 atom and radius 10 A. In the GOLD docking program [57], the default settings were used for all calculations: population size (100); selection pressure (1.1); number of operations (10,000); number of islands (3); niche size (2); crossover frequency (100); mutation frequency (100); migration frequency (10) [58]. Analysis of inhibitor–enzyme interactions of the docked molecules was performed with Discovery Studio Visualizer 5 [34].

### 4.5. Cell Proliferation Assay

The B16F10 murine melanoma cell line was cultured in Dulbecco’s modified Eagle medium (DMEM) (Biowest) supplemented with 100 U/mL streptomycin (Sigma-Aldrich, Taufkirchen, Germany), 100 U/mL penicillin (Sigma-Aldrich, Taufkirchen, Germany), and 10% fetal bovine serum (Sigma-Aldrich, Taufkirchen, Germany). Cells were maintained in 5% CO_2_ at 37 °C.

Cell viability was estimated using the MTT colorimetric assay as described in our previous papers [20,21] and by Bellei et al. [59]. B16 cells were seeded into a 96-well plate (4 × 10^3^ cells/well) and incubated in the presence of investigated thiosemicarbazones in various concentrations (1000, 400, 100, 40, 10, 1, and 0.1 μM) for 48 h. Kojic acid and DMSO were used as controls. MTT was added to wells (0.625 mg/mL, Sigma-Aldrich, Taufkirchen, Germany) for the last 4 h. Then, lysing buffer was added to dissolve insoluble formazan. The absorbance was measured (λ = 570 nm) using a Thermo Lab Multiscan RC microplate reader.

### 4.6. Measurement of Melanin Production 

Measurement of melanin production in the B16F10 murine melanoma cell line was performed as described in our previous papers [20,21]. The cells were seeded into a 96-well plate (5 × 10^3^ cells/well) and stimulated with α-MSH (100 nM, Sigma-Aldrich, Taufkirchen, Germany). After 24 h, cells were treated with the investigated thiosemicarbazones, as well as DMSO and kojic acid, at a final concentration of 100, 40, 10, 4, and 1 μM. After 48 h of incubation with inhibitors, cells were solubilized in 1 N sodium hydroxide (1 N, 60 °C, 2 h). In order to measure melanin content, absorbance was measured (λ = 405 nm) using a microplate reader.

## Figures and Tables

**Table 1:** IC_50_ values of the studied compounds on the diphenolase activity of mushroom tyrosinase.

Compound	IC_50_^1^ (µM)	95% CI ^2^ IC_50_ (µM) (*n* = 3)	Ratio of IC_50_ of Kojic Acid to IC_50_ of TSC
TSC 1	12.2	10.3–14.8	1.6
TSC 2	1.9	1.7–2.5	10.5
TSC 3	1.8	1.3–2.1	11.1
TSC 4	11.3	9.9–12.7	1.8
TSC 5	0.65	0.5–0.7	30.6
TSC 6	0.34	0.3–0.4	58.5
TSC 7	1.2	1.0–1.2	16.5
TSC 8	0.95	0.8–1.0	20.9
TSC 9	0.80	0.7–0.9	24.9
TSC 10	154,5	106.4–224.3	0.1
TSC 11	14.2	12.91–15.79	1.4
TSC 12	1.6	1.622–2.115	12.4
Benzaldehyde TSC	3.4	2.9–4.0	5.6
Acetophenone TSC	1.5	1.3–1.7	13.2
Kojic acid	19.9	17.2–23.9	1.0

^1^ Inhibitor concentration required for 50% inhibition; ^2^ confidence interval.

**Table 2:** Inhibition type, mechanism, and the inhibition constant values of the examined compounds on the diphenolase activity of mushroom tyrosinase.

Compound	Inhibition Mechanism	Inhibition Type	K_i_^1^ ± Standard Error (µM) (*n* = 3)	K_is_^2^ ± Standard Error (µM) (*n* = 3)
TSC 1	reversible	mixed	5.0 ± 1.4	27.5 ± 3.5
TSC 2	reversible	mixed	1.0 ± 0.1	9.7 ± 3.1
TSC 3	reversible	competitive	0.6 ± 0.01	-
TSC 4	reversible	mixed	8.0 ± 0.9	30.4 ± 0.5
TSC 5	reversible	mixed	0.6 ± 0.1	3.05 ± 1.1
TSC 6	reversible	competitive	0.2 ± 0.06	3.5 ± 0.8
TSC 7	reversible	mixed	0.7 ± 0.1	5.3 ± 2.1
TSC 8	reversible	mixed	0.6 ± 0.08	5.4 ± 0.1
TSC 9	reversible	competitive	0.6 ± 0.06	-
TSC 10	reversible	mixed	8.6 ± 0.5	43.3 ± 3.0
TSC 11	reversible	competitive	0.4 ± 0.02	-
TSC 12	reversible	mixed	5.0 ± 1.4	27.5 ± 3.5
Kojic acid	reversible	mixed	26 ± 1	72 ± 3

^1^ Inhibition constant for enzyme–inhibitor complex; ^2^ inhibition constant for substrate–enzyme–inhibitor complex.

**Table 3:** Effectiveness of TSCs in inhibition of melanin production and B16F10 cell proliferation.

Compound	Inhibition of Melanin Production	95% CI ^2^ IC_50_ (µM)	Inhibition of Cell Proliferation	95% CI ^2^ IC_50_ (µM)
	IC_50_^1^ (µM)		IC_50_^1^ (µM)	
TSC 1	39.2	36.57–42.03	45.2	36.43–56.19
TSC 2	13.5	12.45–14.68	22.4	17.15–29.35
TSC 3	13.1	11.68–14.65	20.3	15.03–27.35
TSC 4	207.8	135.2–319.6	3113.4 ^3^	499.3–19414
TSC 5	88.9	81.41–97.12	149.6	124.8–179.4
TSC 6	139.4	126.3–153.9	154.9	141.9–169.0
TSC 7	21.7	18.50–25.49	37.3	31.31–44.38
TSC 8	69.5	60.05–80.48	103.2	87.83–121.3
TSC 9	25.2	19.39–32.75	115.3	99.15–134.1
TSC 10	77.8	74.16–81.57	93.2	83.85–103.6
TSC 11	44.0	38.48–50.43	44.8	40.04–50.22
TSC 12	1160.3 ^4^	219.7–6129	136.6	109.7–170.0
Kojic acid	114.1	105.9–123.0	152.7	138.4–168.6
DMSO	8019.8	140.5–457,651	166.4	153.4–180.6

^1^ Inhibitor concentration required for 50% inhibition; ^2^ confidence interval; ^3^ change in color of the solution during 3-(4,5-dimethylthiazol-2-yl)-2,5-diphenyltetrazolium bromide (MTT) assay affected the IC_50_ value; ^4^ yellow color of the solution affected the spectroscopic measurement at 405 nm. DMSO, dimethyl sulfoxide.

## Supplementary Materials

The following are available online at https://www.mdpi.com/1424-8247/14/1/74/s1: Table S1. GoldScore results for all TSCs; Section 1. Detailed description of the interaction between TSCs 1–12 and the active site of tyrosinase; Figure S1. ^1^H-NMR spectrum of mixture of (*E*)- and (Z)-2’-methoxyacetophenone thiosemicarbazone (TSC 1); Figure S2. Expanded aromatic region of ^1^H-NMR spectrum of mixture of (*E*)- and (Z)-2’-methoxyacetophenone thiosemicarbazone (TSC 1); Figure S3. Expanded aliphatic region of ^1^H-NMR spectrum of mixture of (*E*)- and (Z)-2’-methoxyacetophenone thiosemicarbazone (TSC 1); Figure S4. ^13^C{^1^H}-NMR spectrum of mixture of (*E*)- and (Z)-2’-methoxyacetophenone thiosemicarbazone (TSC 1); Figure S5. Expanded aromatic region of ^13^C{^1^H}-NMR spectrum of mixture of (*E*)- and (Z)-2’-methoxyacetophenone thiosemicarbazone (TSC 1); Figure S6. Expanded aliphatic region of ^13^C{^1^H}-NMR spectrum of mixture of (*E*)- and (Z)-2’-methoxyacetophenone thiosemicarbazone (TSC 1); Figure S7. NOESY spectrum of the mixture of (*E*)- and (Z)-2’-methoxyacetophenone thiosemicarbazone (TSC 1); Figure S8. ^1^H-NMR spectrum of (*E*)-3’-methoxyacetophenone thiosemicarbazone (TSC 2); Figure S9. Expanded aromatic region of ^1^H-NMR spectrum of (*E*)-3’-methoxyacetophenone thiosemicarbazone (TSC 2); Figure S10. ^1^H-NMR spectrum of (*E*)-4’-methoxyacetophenone thiosemicarbazone (TSC 3); Figure S11. Expanded aromatic region of ^1^H-NMR spectrum of (*E*)-4’-methoxyacetophenone thiosemicarbazone (TSC 3); Figure S12. ^1^H-NMR spectrum of mixture of (*E*)- and (Z)-2’-aminoacetophenone thiosemicarbazone (TSC 4); Figure S13. Expanded aromatic region of ^1^H-NMR spectrum of mixture of (*E*)- and (Z)-2’-aminoacetophenone thiosemicarbazone (TSC 4); Figure S14. Expanded aliphatic region of ^1^H-NMR spectrum of mixture of (*E*)- and (Z)-2’-methoxyacetophenone thiosemicarbazone (TSC 4); Figure S15. ^13^C{^1^H}-NMR spectrum of mixture of (*E*)- and (Z)-2’-aminoacetophenone thiosemicarbazone (TSC 4); Figure S16. Expanded aromatic region of ^13^C{^1^H}-NMR spectrum of mixture of (*E*)- and (Z)-2’-aminoacetophenone thiosemicarbazone (TSC 4); Figure S17. Expanded aliphatic region of ^13^C{^1^H}-NMR spectrum of mixture of (*E*)- and (Z)-2’-aminoacetophenone thiosemicarbazone (TSC 4); Figure S18. NOESY spectrum of the mixture of (*E*)- and (Z)-2’-aminoacetophenone thiosemicarbazone (TSC 4); Figure S19. ^1^H-NMR spectrum of mixture of (*E*)-3’-aminoacetophenone thiosemicarbazone (TSC 5); Figure S20. Expanded aromatic region of ^1^H-NMR spectrum of (*E*)-3’-aminoacetophenone thiosemicarbazone (TSC 5); Figure S21. ^1^H-NMR spectrum of mixture of (*E*)-4’-aminoacetophenone thiosemicarbazone (TSC 6); Figure S22. Expanded aromatic region of ^1^H-NMR spectrum of (*E*)-4’-aminoacetophenone thiosemicarbazone (TSC 6); Figure S23. ^1^H-NMR spectrum of mixture of (*E*)- and (Z)-2’-hydroxyacetophenone thiosemicarbazone (TSC 7); Figure S24. Expanded aromatic region of ^1^H-NMR spectrum of mixture of (*E*)- and (Z)-2’-hydroxyacetophenone thiosemicarbazone (TSC 7); Figure S25. Expanded aliphatic region of ^1^H-NMR spectrum of mixture of (*E*)- and (Z)-2’-hydroxyacetophenone thiosemicarbazone (TSC 7); Figure S26. ^13^C-NMR spectrum of mixture of (*E*)- and (Z)-2’-hydroxyacetophenone thiosemicarbazone (TSC 7); Figure S27. Expanded aromatic region of ^13^C-NMR spectrum of mixture of (*E*)- and (Z)-2’-hydroxyacetophenone thiosemicarbazone (TSC 7); Figure S28. NOESY spectrum of mixture of (*E*)- and (Z)-2’-hydroxyacetophenone thiosemicarbazone (TSC 7); Figure S29. ^1^H-NMR spectrum of methanol solvate of (*E*)-3’-hydroxyacetophenone thiosemicarbazone (TSC 8•CH_3_OH); Figure S30. Expanded aromatic region of ^1^H-NMR spectrum of methanol solvate of (*E*)-3’-hydroxyacetophenone thiosemicarbazone (TSC 8•CH_3_OH); Figure S31. ^13^C-NMR spectrum of methanol solvate of (*E*)-3’-hydroxyacetophenone thiosemicarbazone (TSC 8•CH_3_OH); Figure S32. Expanded aromatic region of ^13^C-NMR spectrum of methanol solvate of (*E*)-3’-hydroxyacetophenone thiosemicarbazone (TSC 8•CH_3_OH); Figure S33. ^1^H-NMR spectrum of (*E*)-4’-hydroxyacetophenone thiosemicarbazone (TSC 9); Figure S34. Expanded aromatic region of ^1^H-NMR spectrum of (*E*)-4’-hydroxyacetophenone thiosemicarbazone (TSC 9); Figure S35. ^1^H-NMR spectrum of (*E*)-2’-nitroacetophenone thiosemicarbazone (TSC 10); Figure S36. Expanded aromatic region of ^1^H-NMR spectrum of (*E*)-2’-nitroacetophenone thiosemicarbazone (TSC 10); Figure S37. ^1^H-NMR spectrum of (*E*)-3’-nitroacetophenone thiosemicarbazone (TSC 11); Figure S38. Expanded aromatic region of ^1^H-NMR spectrum of (*E*)-3’-nitroacetophenone thiosemicarbazone (TSC 11); Figure S39. ^1^H-NMR spectrum of (*E*)-4’-nitroacetophenone thiosemicarbazone (TSC 12); Figure S40. Expanded aromatic region of ^1^H-NMR spectrum of (*E*)-4’-nitroacetophenone thiosemicarbazone (TSC 12); Section 2. Enzyme preparation.
