# Supplementary material for: Monosubstituted Acetophenone Thiosemicarbazones as Potent Inhibitors of Tyrosinase: Synthesis, Inhibitory Studies, and Molecular Docking
Source: Pharmaceuticals (Basel). 2021 Jan 18;14(1):74. doi: 10.3390/ph14010074 (PMC7831505; doi:10.3390/ph14010074)
Supplement: Supplementary file 1 [file pharmaceuticals-14-00074-s001.pdf]

ELECTRONIC SUPPLEMENTARY INFORMATION

Monosubstituted acetophenone thiosemicarbazones as potent inhibitors of tyrosinase: Synthesis, inhibitory studies and molecular docking

Katarzyna Hałdys [1,*], Waldemar Goldeman [2], Natalia Anger-Góra [3], Joanna Rossowska [4] and Rafał Latajka [5,*]

[1] Department of Bioorganic Chemistry, Wrocław University of Science and Technology, 50-370 Wrocław, Poland
[2] Department of Organic and Medicinal Chemistry, Wrocław University of Science and Technology, 50-370 Wrocław, Poland; waldemar.goldeman@pwr.edu.pl
[3] Ludwik Hirszfeld Institute of Immunology and Experimental Therapy, Polish Academy of Science, 53-114 Wrocław, Poland; natalia.anger@hirszfeld.pl (N.A.-G.); joanna.rossowska@hirszfeld.pl (J.R.)
* Correspondence: katarzyna.haldys@pwr.edu.pl (K.H.); rafal.latajka@pwr.edu.pl (R.L.)

Table S1. GoldScore results for all TSCs.

Compound	GoldScore
TSC 1	57.9
TSC 2	56.2
TSC 3	57.9
TSC 4	57.3
TSC 5	56.8
TSC 6	55.9
TSC 7	58.3
TSC 8	56.2
TSC 9	57.1
TSC 10	59.2
TSC 11	59.4
TSC 12	55.2

Section 1. Detailed description of the interaction between TSCs 1-12 with the active site of tyrosinase

TSC 1

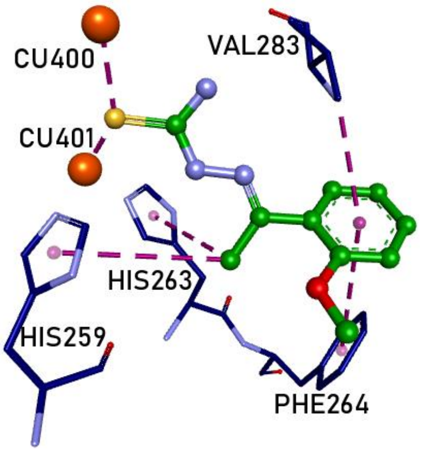

Sulfur atom of the thiourea moiety interacts with copper ions Cu400 (2.56 A) and Cu401 (2.05 A). The structure is stabilized by hydrophobic interactions: π-π interaction between the aromatic ring of the inhibitor and Phe264 (4.72 A), π-sigma between the aromatic ring and Val283 (3.65 A) and π-alkyl interaction of CH_3 group of the inhibitor with imidazole of His259 (4.83 A) and His263 (4.12 A). There are no interactions of the methoxy group substituted on the aromatic ring of the inhibitor.

TSC 2

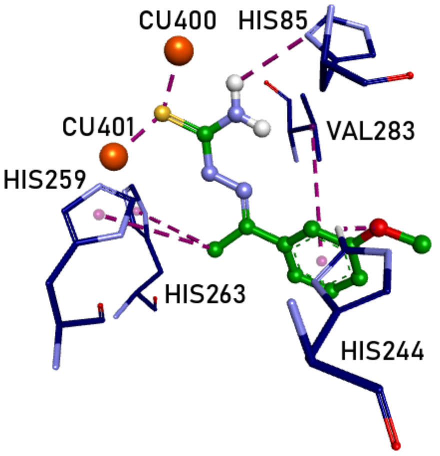

Sulfur atom of the thiourea moiety interact with catalytic copper ions Cu400 (2.23 A) and Cu401 (2.32 A). Moreover, the structure is stabilized by hydrophobic interactions: π-alkyl interactions of the CH_3 group of the inhibitor with imidazole of His259 (4.59 A) and His263 (4.14 A) as well as the aromatic ring of the inhibitor with Val283 (5.65 A). One of the hydrogens of the terminal amino group in thiosemicarbazide moiety forms a hydrogen bond with nitrogen in the imidazole ring of His85 (2.92 A) and one of the hydrogen of imidazole ring of His244 and oxygen of methoxy group of the inhibitor (2.17 A).

TSC 3

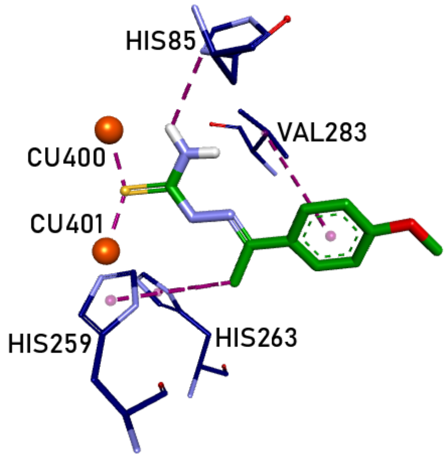

Sulfur atom of the thiourea moiety interact with catalytic copper ions Cu400 (2.23 A) and Cu401 (2.31 A). Moreover, the structure is stabilized by hydrophobic interactions: π-alkyl interactions of the CH_3 group of the inhibitor with imidazole of His259 (4.58 A) and His263 (4.06 A) as well as the aromatic ring of the inhibitor with Val283 (5.53 A). One of the hydrogens of the terminal amino group in thiosemicarbazide moiety forms a hydrogen bond with nitrogen in the imidazole ring of His85 (3.00 A). There are no interactions of the methoxy group substituted on the aromatic ring of the inhibitor.

TSC 4

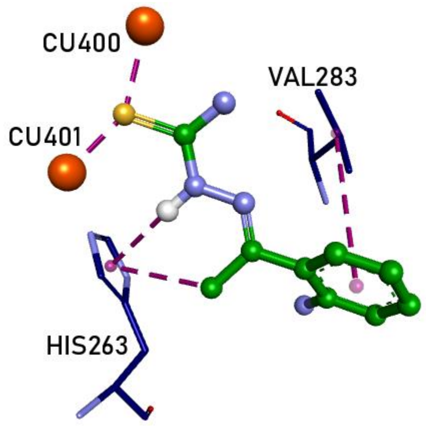

Sulfur atom of the thiourea moiety interacts with copper ions Cu400 (2.36 A) and Cu401 (2.16 A). Moreover, the structure is stabilized by hydrophobic interactions: π-sigma interaction of the CH_3 group of the inhibitor with imidazole of His263 (3.42 A) as well as π-alkyl of the aromatic ring of the inhibitor with Val283 (4.77 A). Hydrogen of the hydrazine group a hydrogen bond with the imidazole ring (π donor) of His263 (2.92 A). There are no interactions of the amino group substituted on the aromatic ring of the inhibitor.

TSC 5

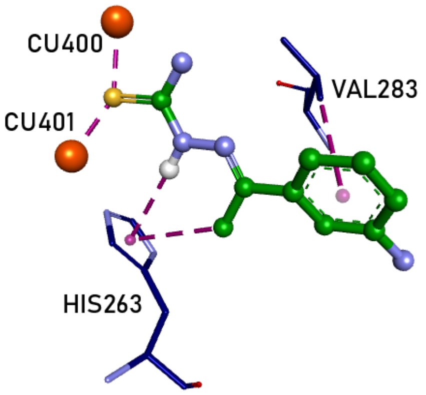

Sulfur atom of the thiourea moiety interacts with copper ions Cu400 (2.39 A) and Cu401 (2.20 A). Moreover, the structure is stabilized by hydrophobic interactions: π-sigma interaction of the CH_3 group of the inhibitor with imidazole of His263 (3.31 A) as well the aromatic ring of the inhibitor with Val283 (3.15 A). Hydrogen of the hydrazine group forms a hydrogen bond with the imidazole ring (π donor) of His263 (2.81 A). There are no interactions of the amino group substituted on the aromatic ring of the inhibitor.

TSC 6

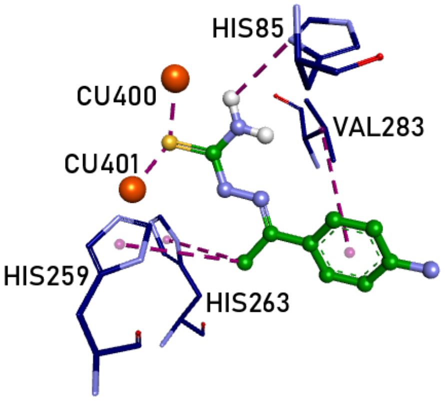

Sulfur atom of the thiourea moiety interacts with copper ions with catalytic copper ions Cu400 (2.30 A) and Cu401 (2.24 A). Moreover, the structure is stabilized by hydrophobic interactions: π-alkyl interactions of the CH_3 group of the inhibitor with imidazole of His259 (4.69 A) and His263 (4.24 A) as well as the aromatic ring of the inhibitor with Val283 (5.90 A). One of the hydrogens of the terminal amino group in thiosemicarbazide moiety forms a hydrogen bond with nitrogen in the imidazole ring of His85 (3.00 A). There are no interactions of the amino group substituted on the aromatic ring of the inhibitor.

TSC 7

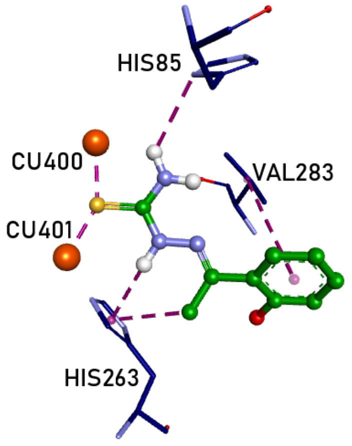

Sulfur atom of the thiourea moiety interacts with copper ions Cu400 (2.33 A) and Cu401 (2.20 A). Moreover, the structure is stabilized by hydrophobic interactions: π-sigma interaction of the CH_3 group of the inhibitor with imidazole of His263 (3.40 A) as well a π-alkyl of the aromatic ring of the inhibitor with Val283 (4.72 A). Hydrogen of the hydrazine group forms a hydrogen bond with the imidazole ring (π donor) of His263 (2.93 A) and one of the hydrogens of the terminal amino group in thiosemicarbazide moiety forms a hydrogen bond with nitrogen in the imidazole ring of His85 (3.09 A) There are no interactions of the amino group substituted on the aromatic ring of the inhibitor.

TSC 8

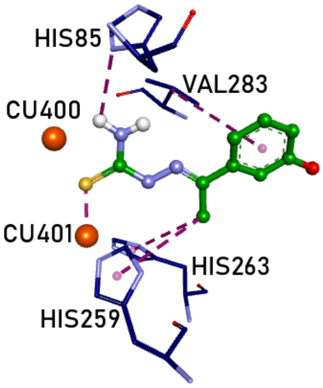

Sulfur atom of the thiourea moiety interact with catalytic copper ion Cu401 (2.21 A). Moreover, the structure is stabilized by hydrophobic interactions: π-alkyl interactions of the CH_3 group of the inhibitor with imidazole of His259 (4.66 A) and His263 (4.45 A) as well as the aromatic ring of the inhibitor with Val283 (5.46 A). One of the hydrogens of the terminal amino group in thiosemicarbazide moiety forms a hydrogen bond with nitrogen in the imidazole ring of His85 (3.04 A). There are no interactions of the hydroxyl group substituted on the aromatic ring of the inhibitor.

TSC 9

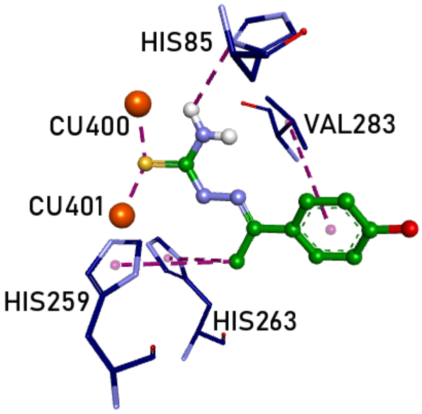

Sulfur atom of the thiourea moiety interacts with copper ions Cu400 (2.30 A) and Cu401 (2.24 A). Moreover, the structure is stabilized by hydrophobic interactions: π-alkyl interactions of the CH_3 group of the inhibitor with imidazole of His259 (4.55 A) and His263 (4.23 A) as well as the aromatic ring of the inhibitor with Val283 (5.54 A). One of the hydrogens of the terminal amino group in thiosemicarbazide moiety forms a hydrogen bond with nitrogen in the imidazole ring of His85 (3.00 A). There are no interactions of the hydroxyl group substituted on the aromatic ring of the inhibitor.

TSC 10

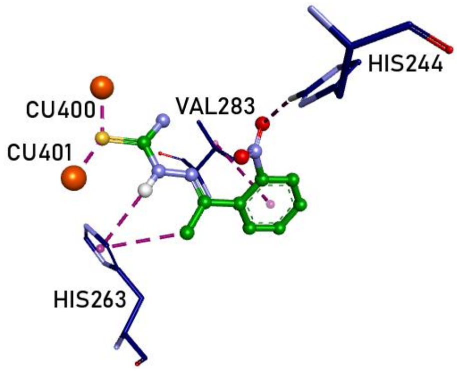

Sulfur atom of the thiourea moiety interacts with copper ions Cu400 (2.37 A) and Cu401 (2.16 A). Moreover, the structure is stabilized by hydrophobic interactions: π-sigma interaction of the CH_3 group of the inhibitor with imidazole of His263 (3.65 A) as well a π-alkyl of the aromatic ring of the inhibitor with Val283 (4.60 A). Hydrogen of the hydrazine group forms a hydrogen bond with the imidazole ring (π donor) of His263 (3.05 A) and one of the hydrogen of imidazole ring of His244 forms a hydrogen bond with oxygen of nitro group of the inhibitor (2.69 A).

TSC11

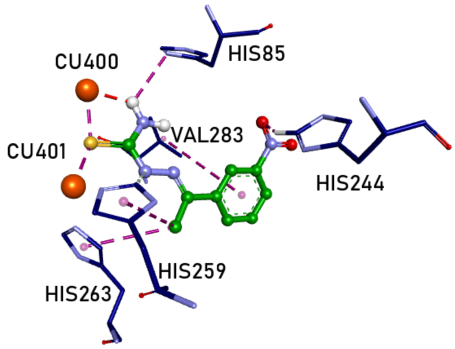

Sulfur atom of the thiourea moiety interacts with copper ions Cu400 (2.30 A) and Cu401 (2.24 A). Moreover, the structure is stabilized by hydrophobic interactions: π-alkyl interactions of the CH_3 group of the inhibitor with imidazole of His259 (4.60 A) and His263 (4.08 A) as well as the aromatic ring of the inhibitor with Val283 (5.47 A). One of the hydrogens of the terminal amino group in thiosemicarbazide moiety forms a hydrogen bond with nitrogen in the imidazole ring of His85 (3.03 A) and one of the hydrogen of imidazole ring of His244 forms a hydrogen bond with oxygen of nitro group of the inhibitor (2.24 A).

TSC 12

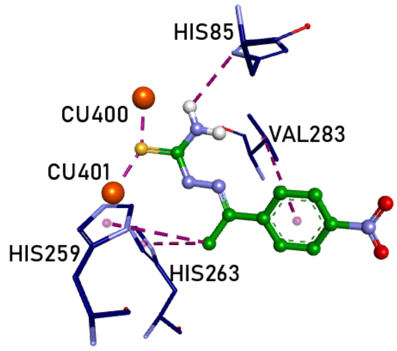

Sulfur atom of the thiourea moiety interacts with copper ions Cu400 (2.22 A) and Cu401 (2.29 A). Moreover, the structure is stabilized by hydrophobic interactions: π-alkyl interactions of the CH_3 group of the inhibitor with imidazole of His259 (4.54 A) and His263 (4.08 A) as well as the aromatic ring of the inhibitor with Val283 (5.54 A). One of the hydrogens of the terminal amino group in thiosemicarbazide moiety forms a hydrogen bond with nitrogen in the imidazole ring of His85 (2.94 A). There are no interactions of the nitro group substituted on the aromatic ring of the inhibitor.

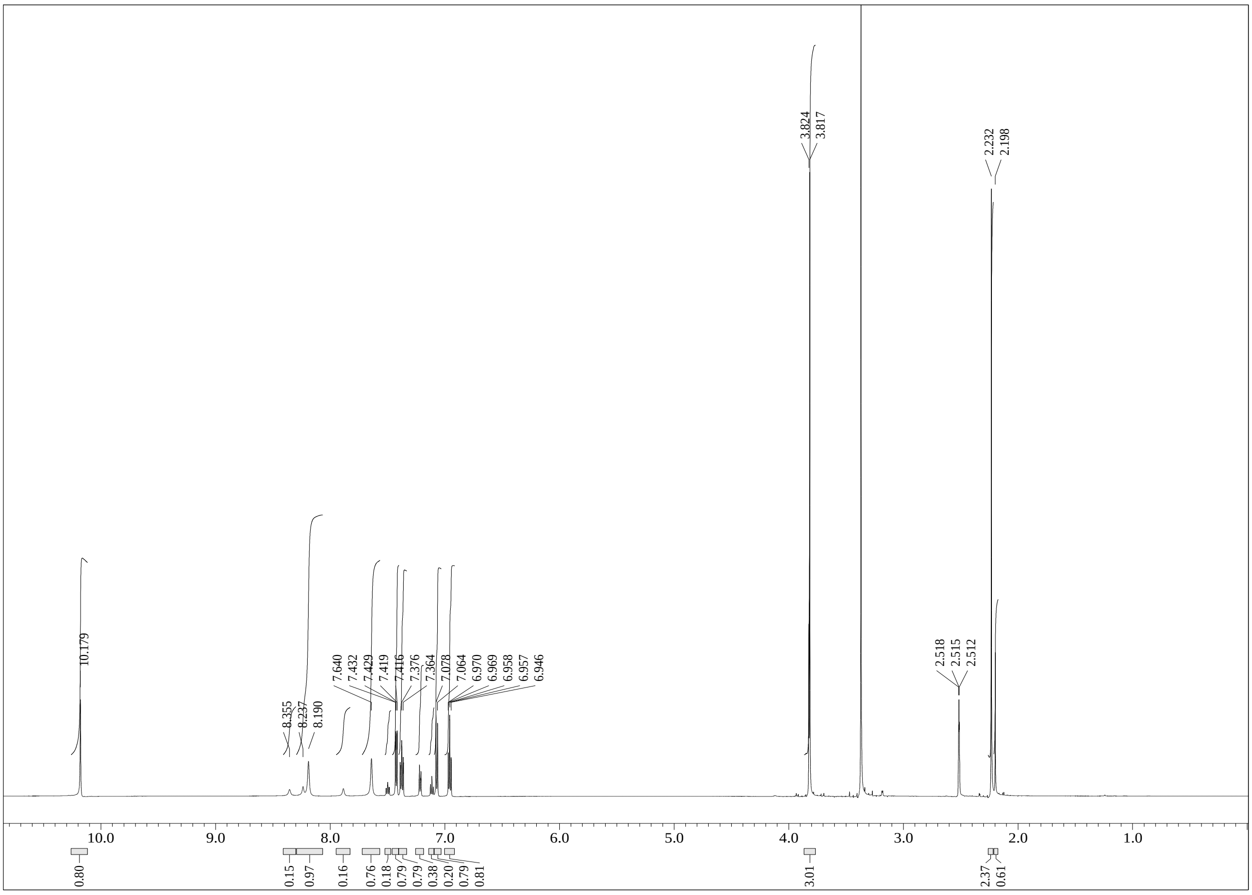

Figure S1. ^{1}H NMR spectrum of mixture of (*E*)- and (Z)-2′-methoxyacetophenone thiosemicarbazone (TSC 1)

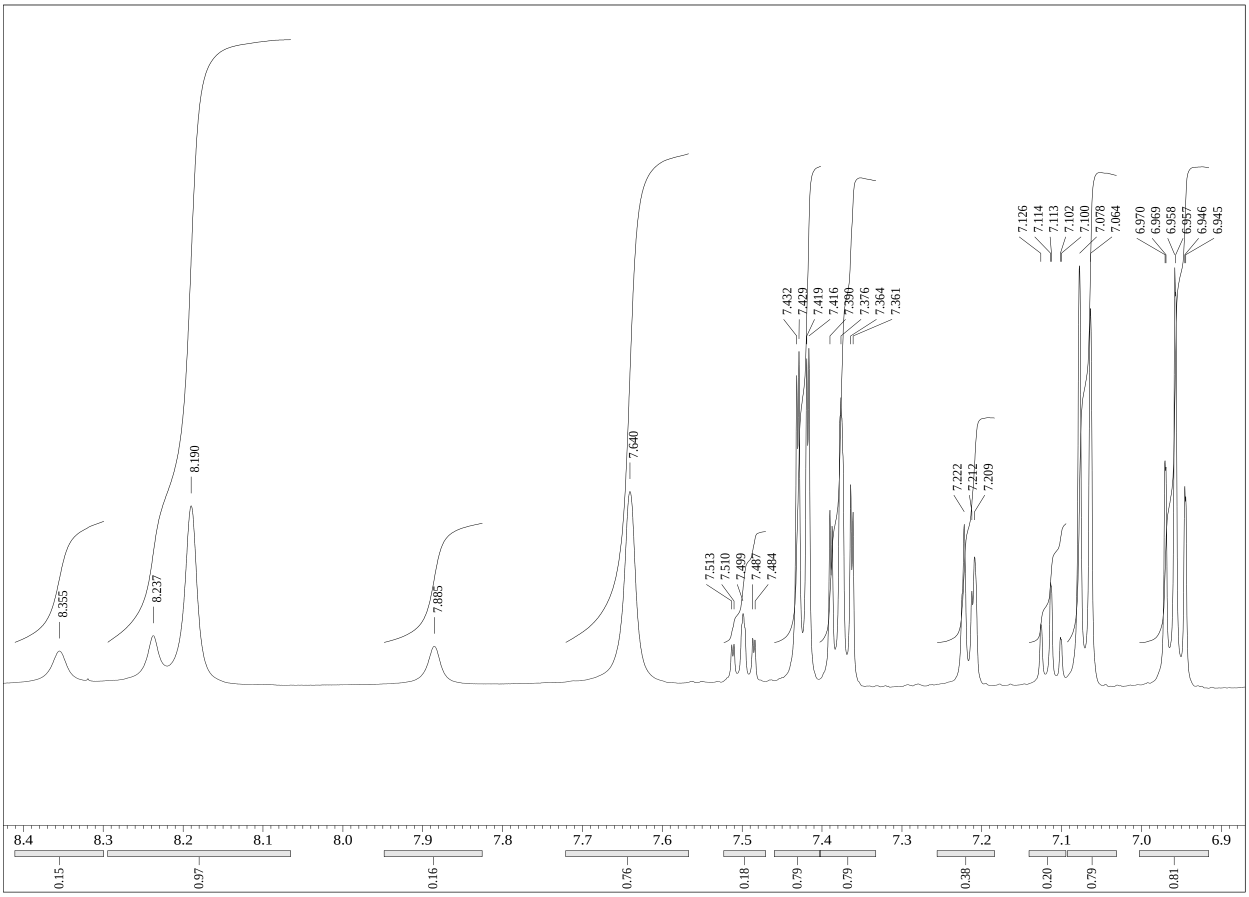

Figure S2. Expanded aromatic region of ^{1}H NMR spectrum of mixture of (*E*)- and (Z)-2′-methoxyacetophenone thiosemicarbazone (TSC 1)

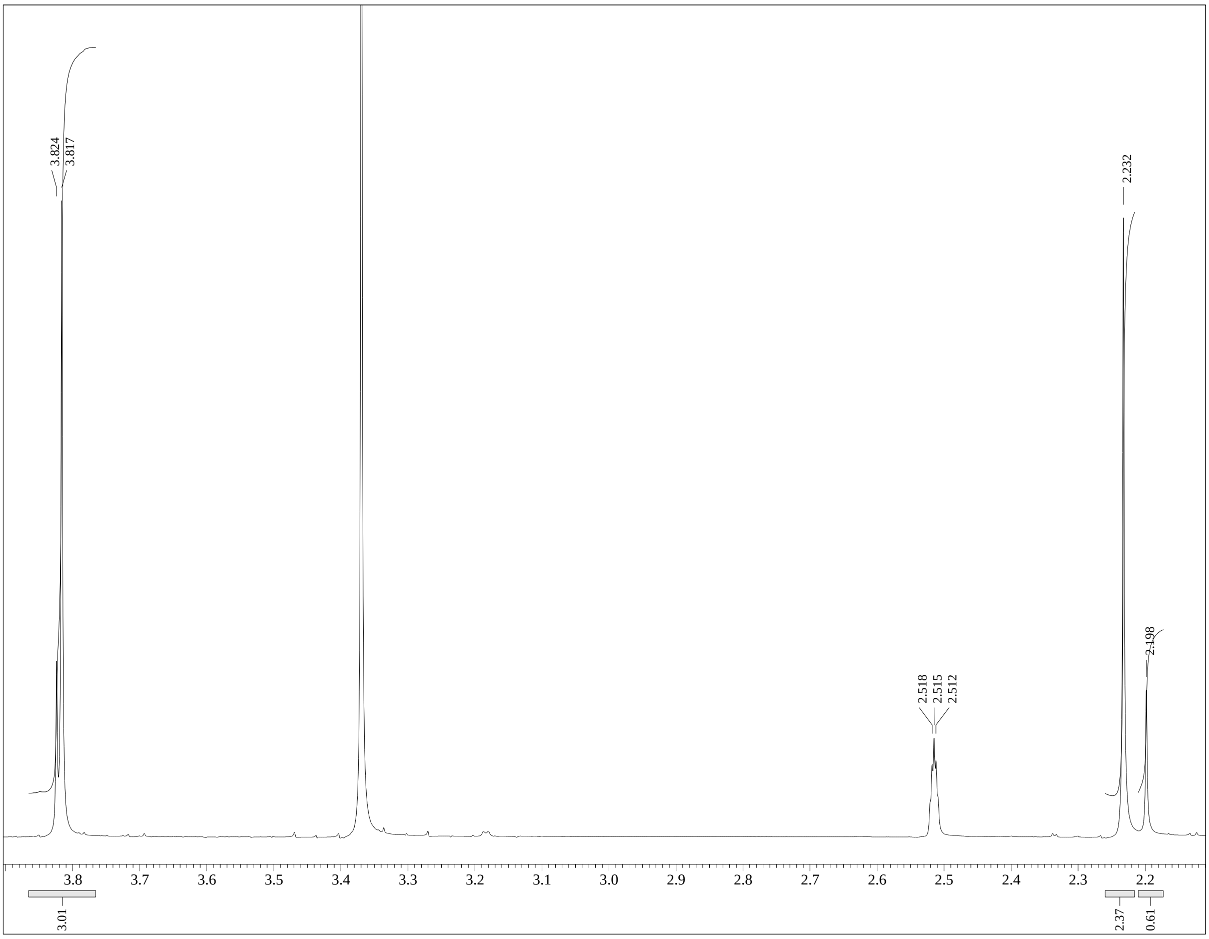

Figure S3. Expanded aliphatic region of ^{1}H NMR spectrum of mixture of (*E*)- and (Z)-2′-methoxyacetophenone thiosemicarbazone (TSC 1)

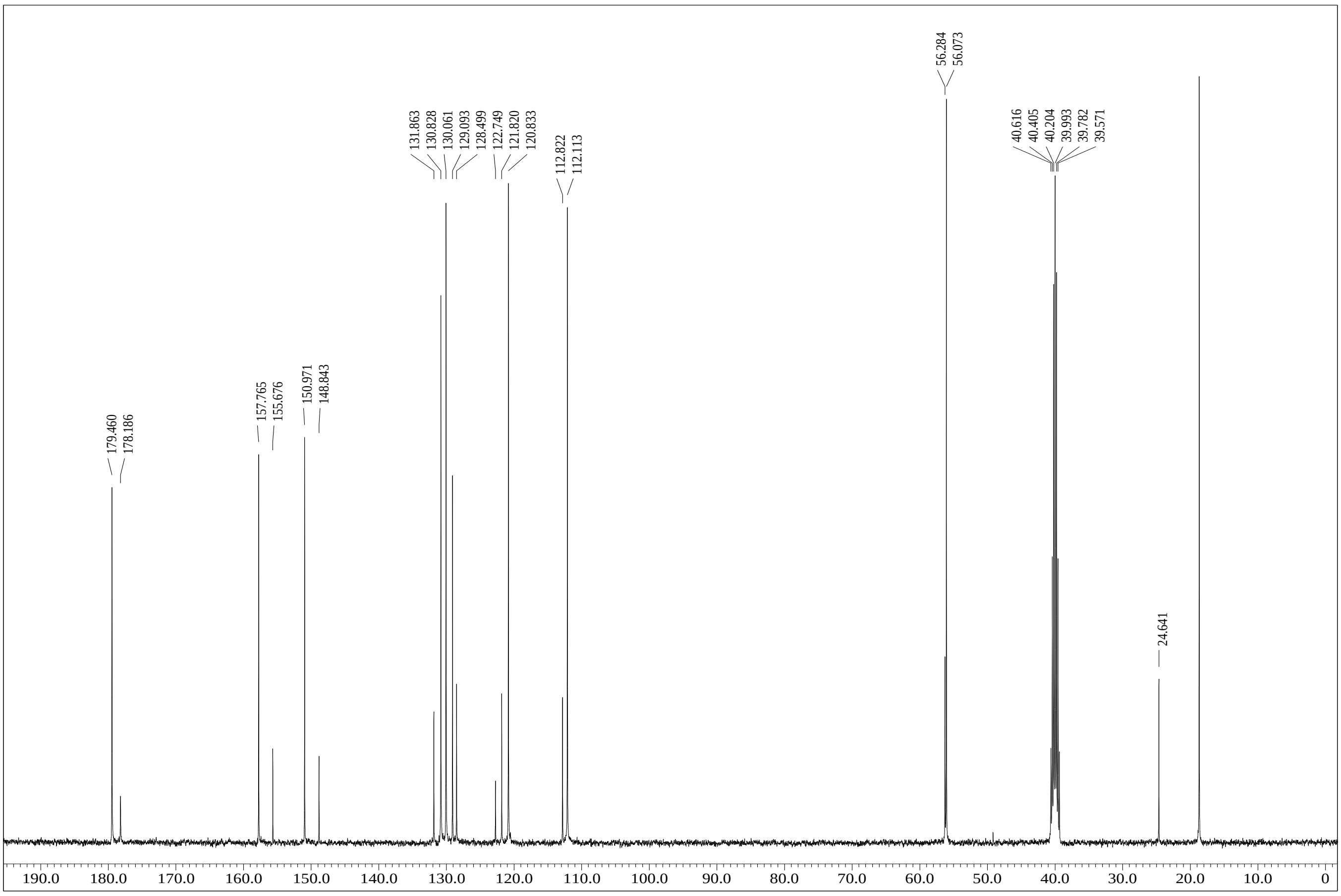

Figure S4. ^{13}C{^{1}H} NMR spectrum of mixture of (*E*)- and (Z)-2′-methoxyacetophenone thiosemicarbazone (TSC 1)

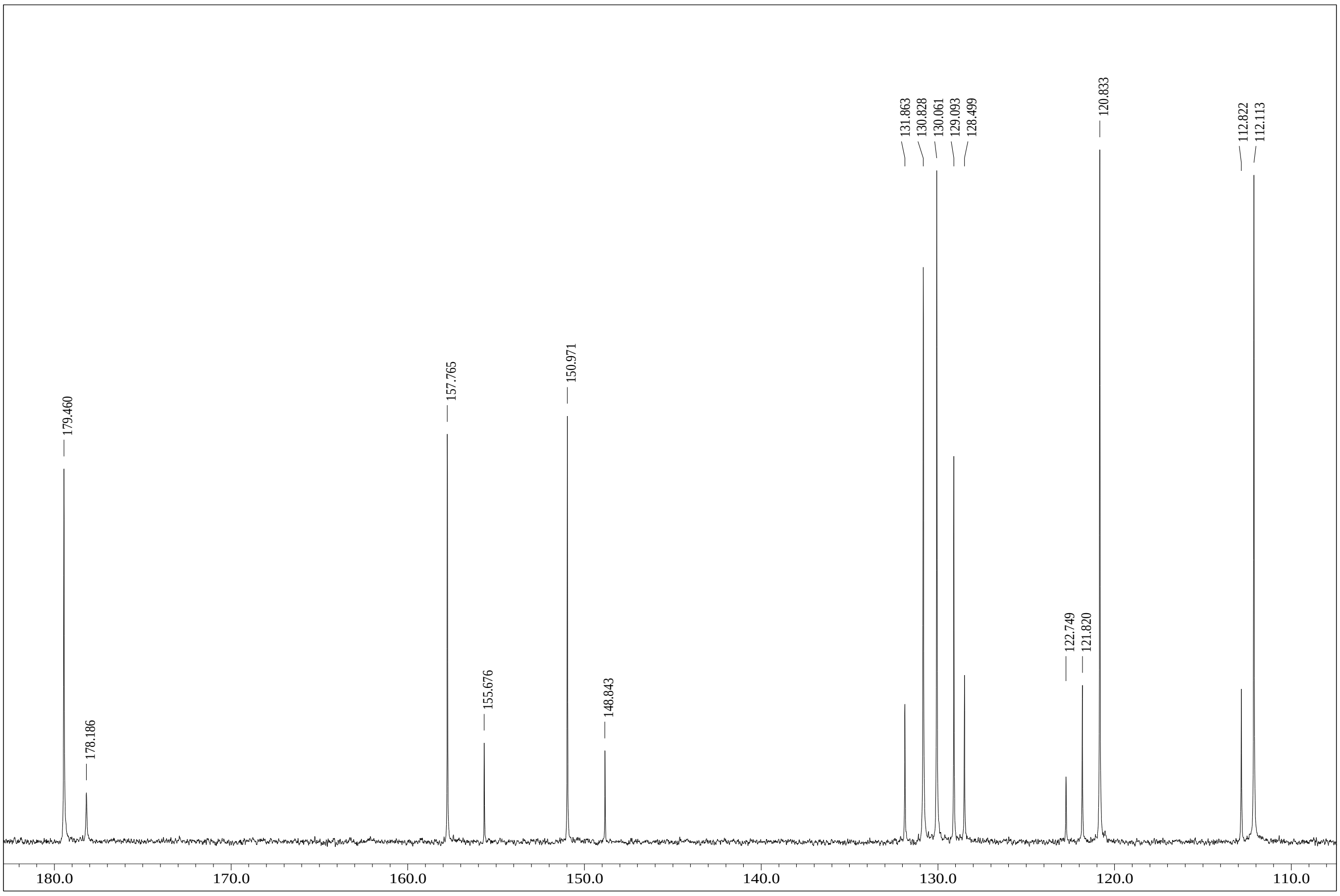

Figure S5. Expanded aromatic region of $^{13}C\{^{1}H\}$ NMR spectrum of mixture of (*E*)- and (Z)-2′-methoxyacetophenone thiosemicarbazone (TSC 1)

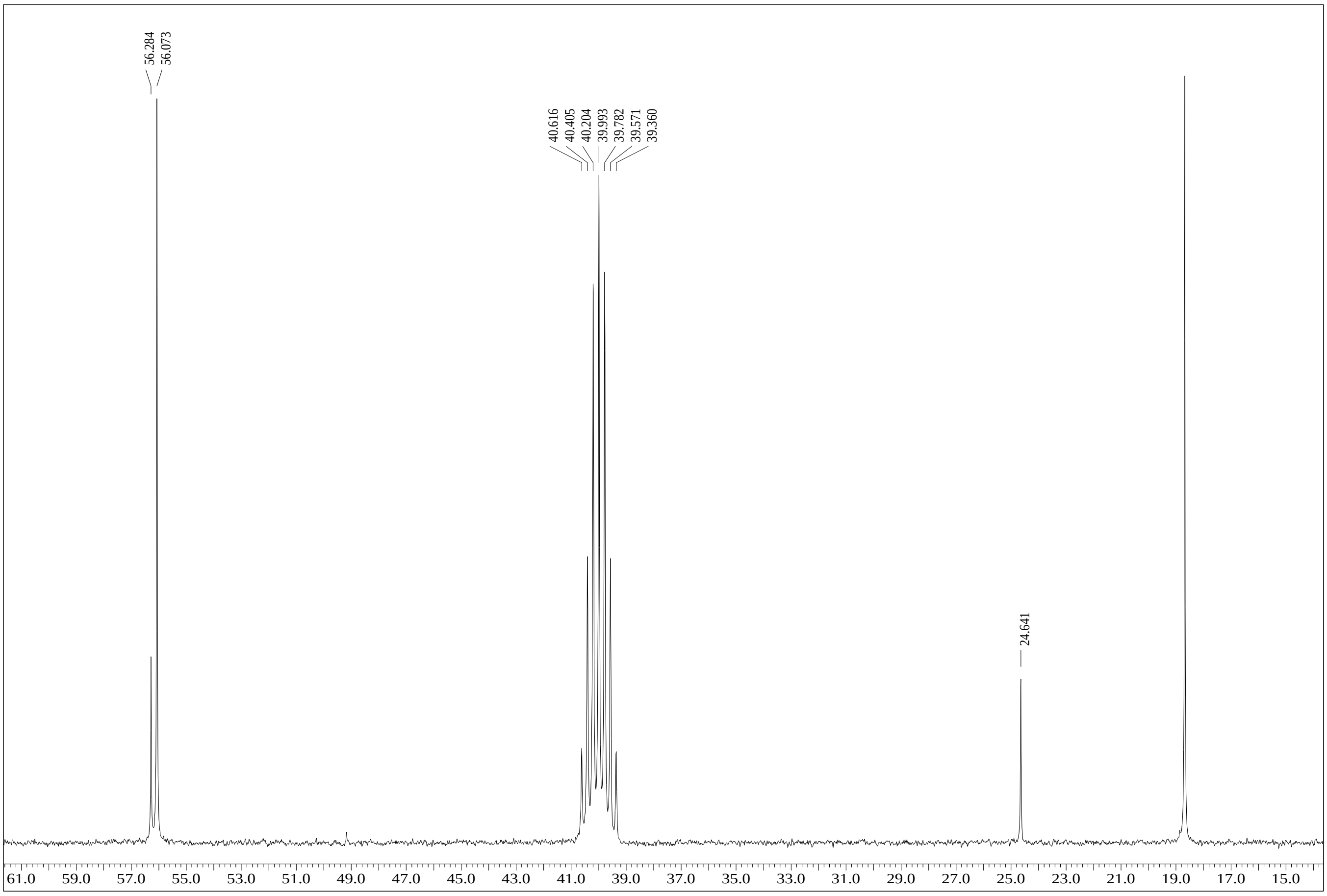

Figure S6. Expanded aliphatic region of $^{13}C\{^{1}H\}$ NMR spectrum of mixture of (*E*)- and (Z)-2′-methoxyacetophenone thiosemicarbazone (TSC 1)

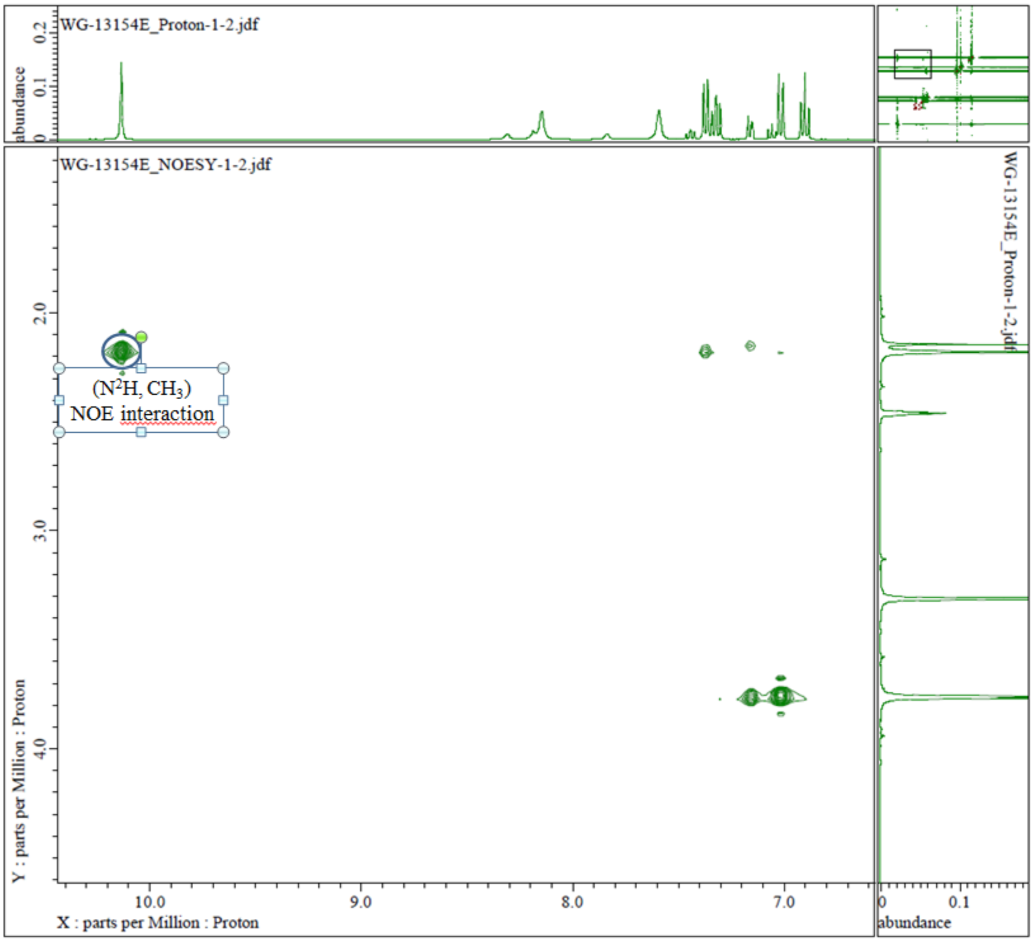

Figure S7. NOESY spectrum of the mixture of (*E*)- and (*Z*)-2′-methoxyacetophenone thiosemicarbazone (TSC 1)

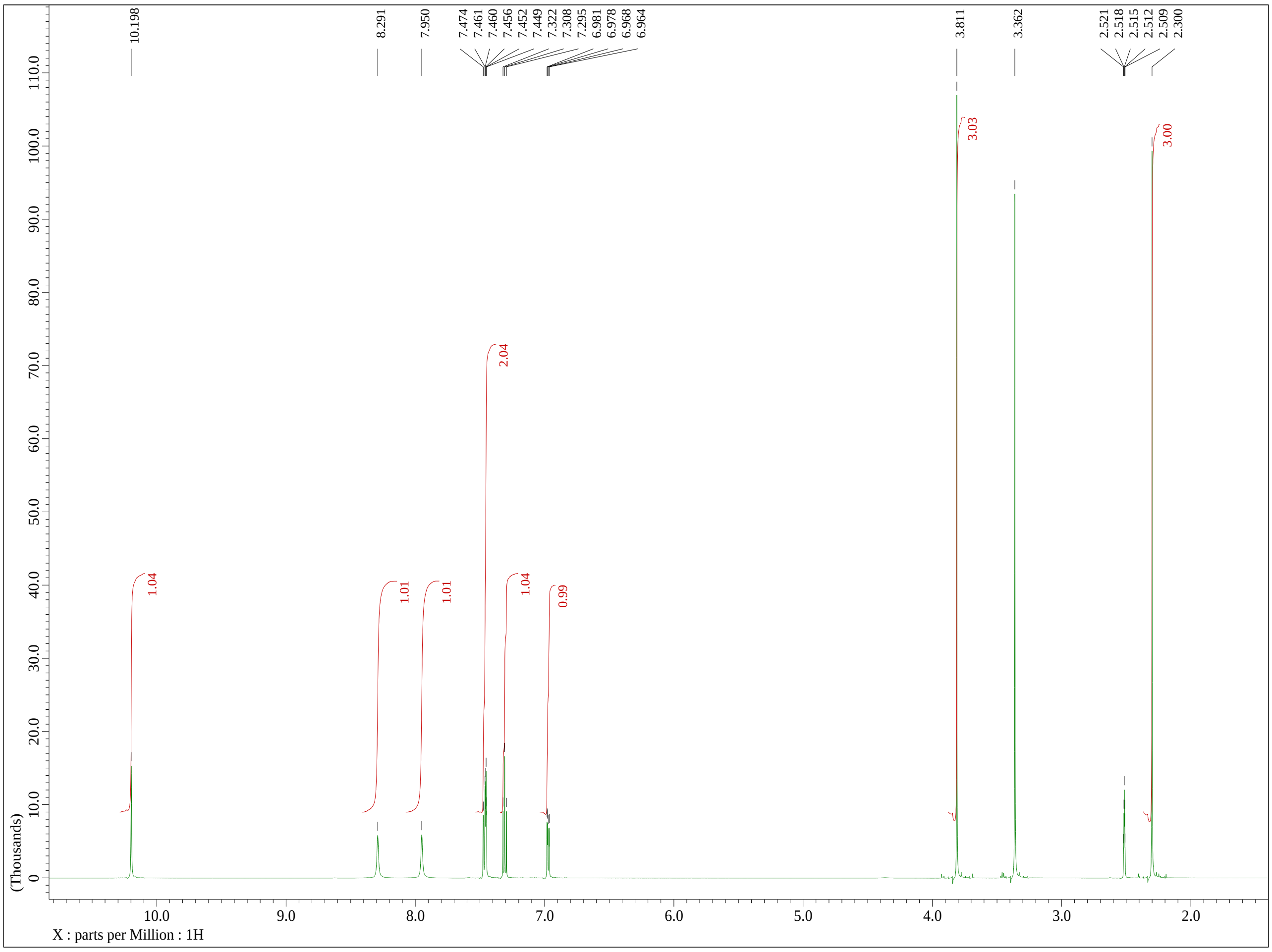

Figure S8. ¹H NMR spectrum of (*E*)-3′-methoxyacetophenone thiosemicarbazone (TSC 2)

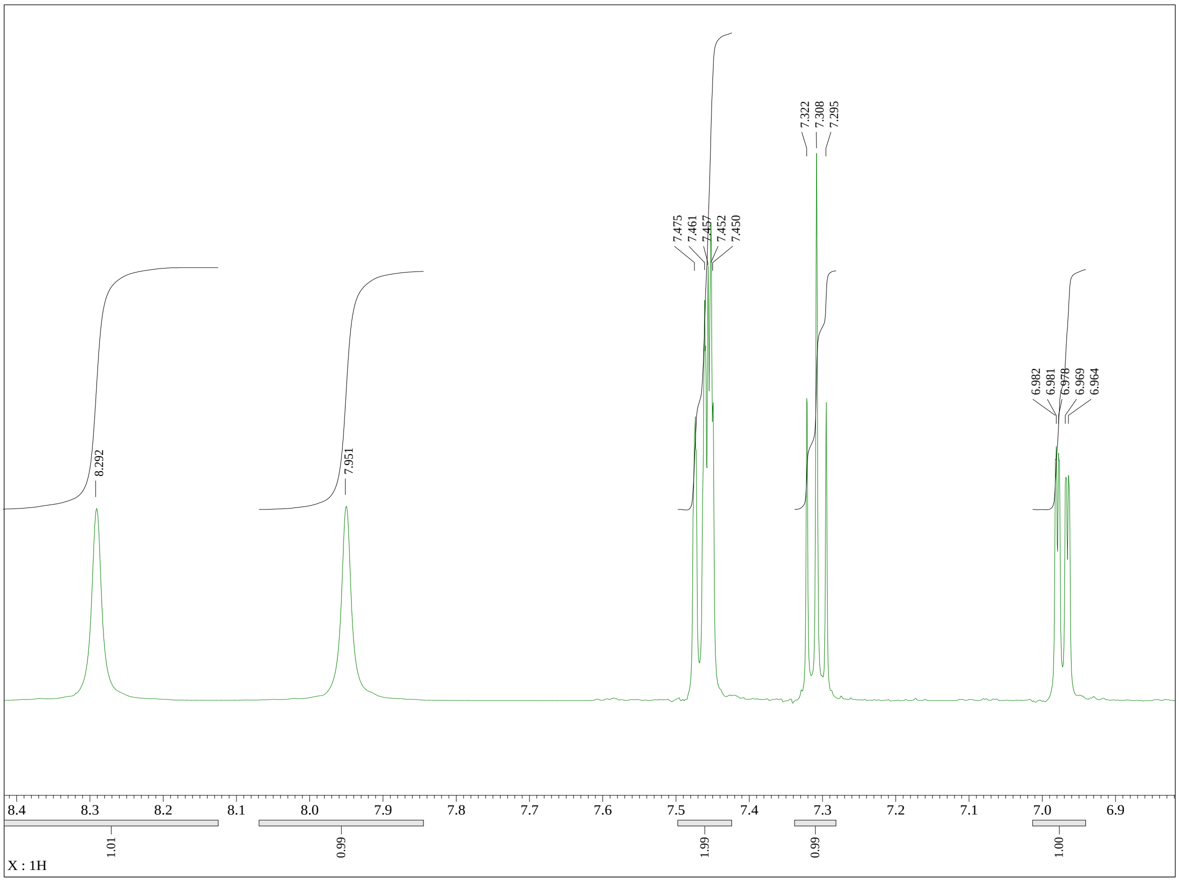

Figure S9. Expanded aromatic region of ^{1}H NMR spectrum of (*E*)-3′-methoxyacetophenone thiosemicarbazone (TSC 2)

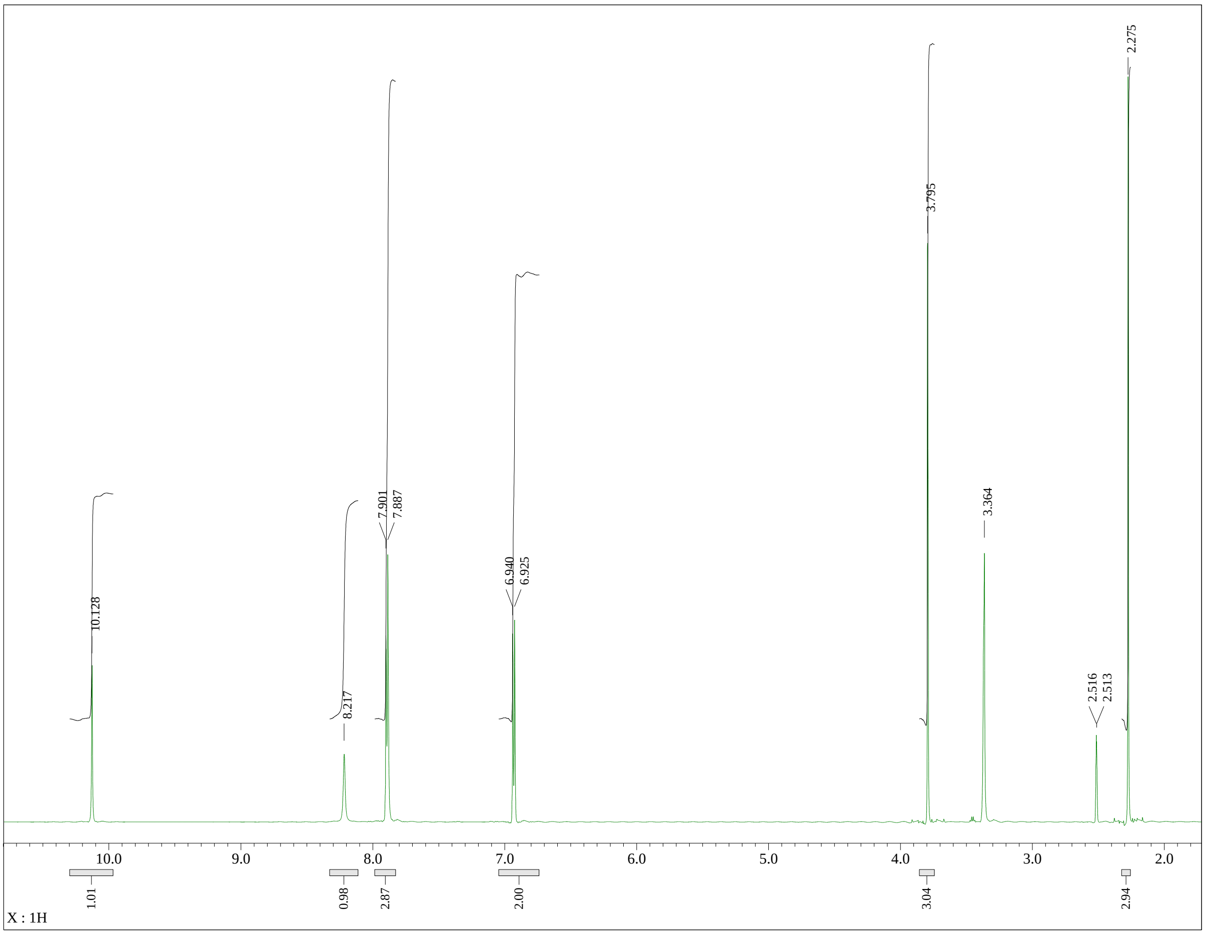

Figure S10. ^{1}H NMR spectrum of (*E*)-4′-**methoxy**acetophenone thiosemicarbazone (TSC 3)

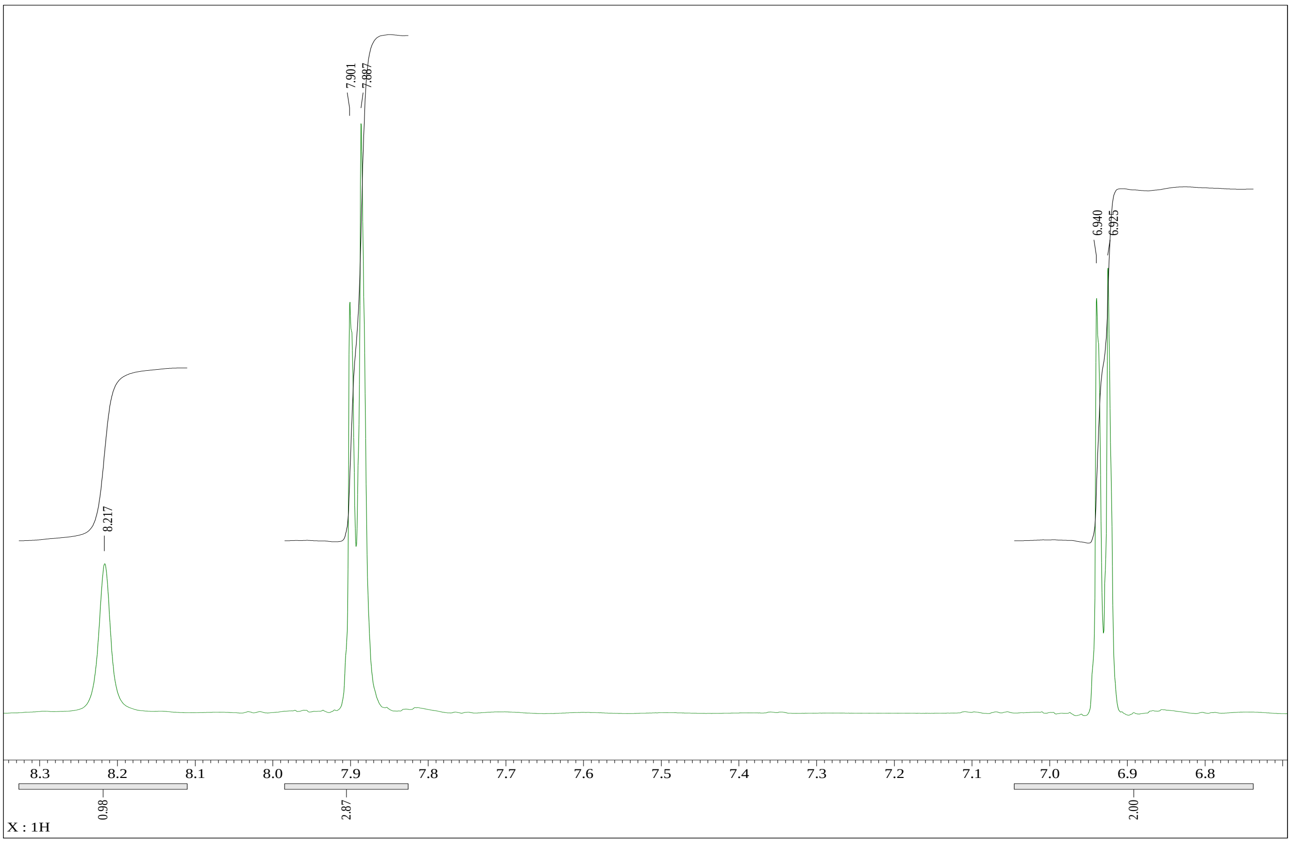

Figure S11. Expanded aromatic region of ^{1}H NMR spectrum of (*E*)-4′-**methoxy**acetophenone thiosemicarbazone (TSC 3)

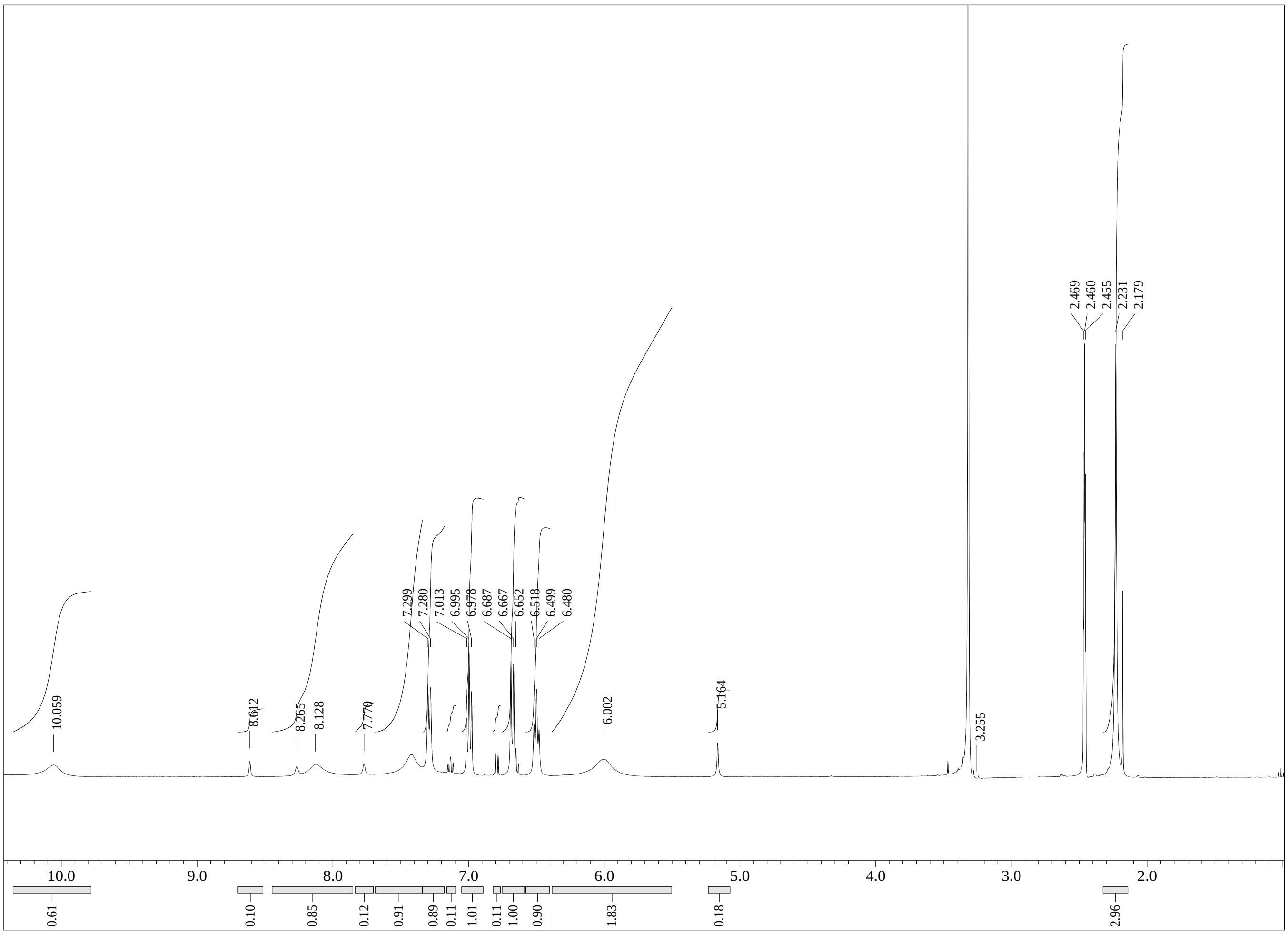

Figure S12. ^{1}H NMR spectrum of mixture of (*E*)- and (*Z*)-2′-aminoacetophenone thiosemicarbazone (TSC 4)

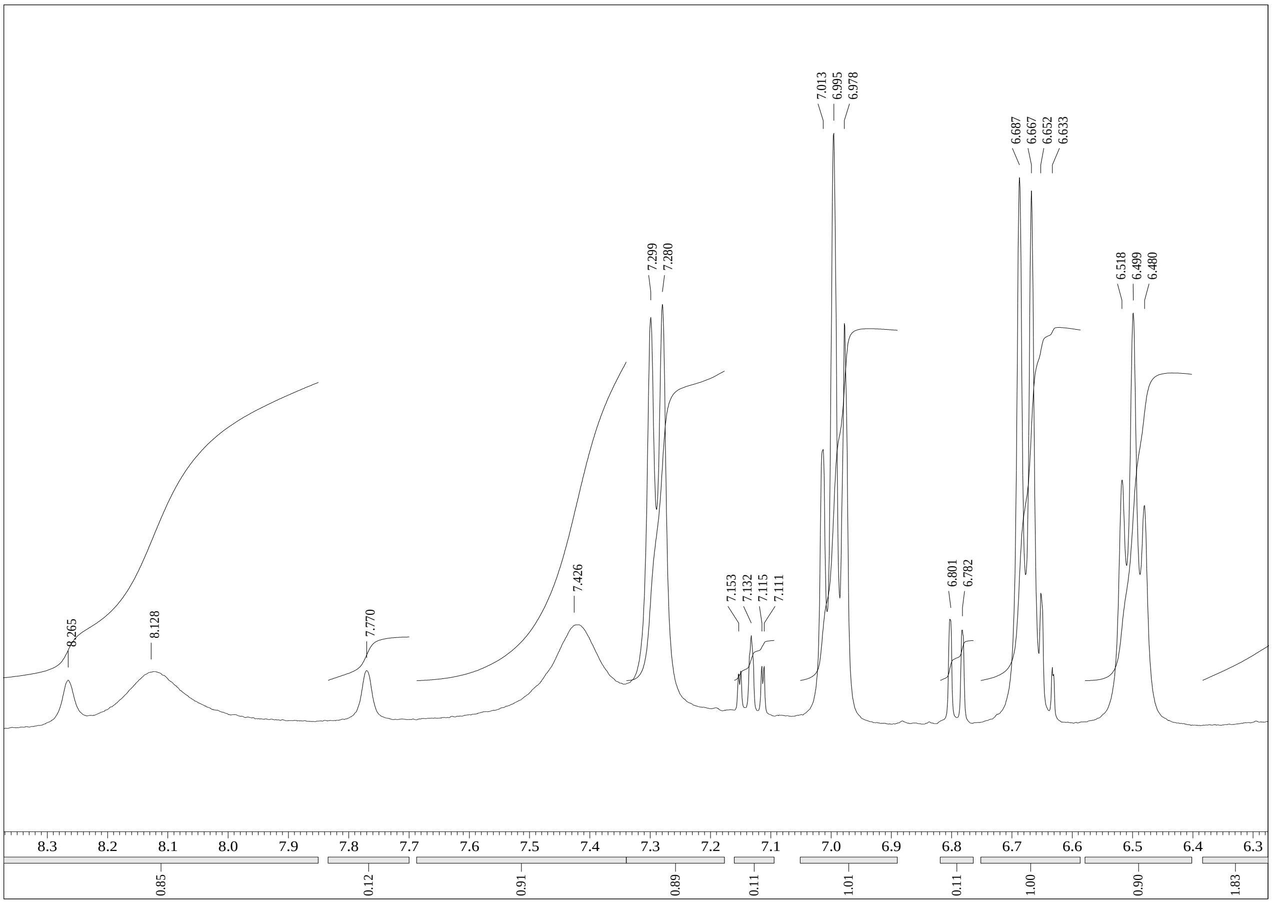

Figure S13. Expanded aromatic region of ^{1}H NMR spectrum of mixture of (*E*)- and (Z)-2′-aminoacetophenone thiosemicarbazone (TSC 4)

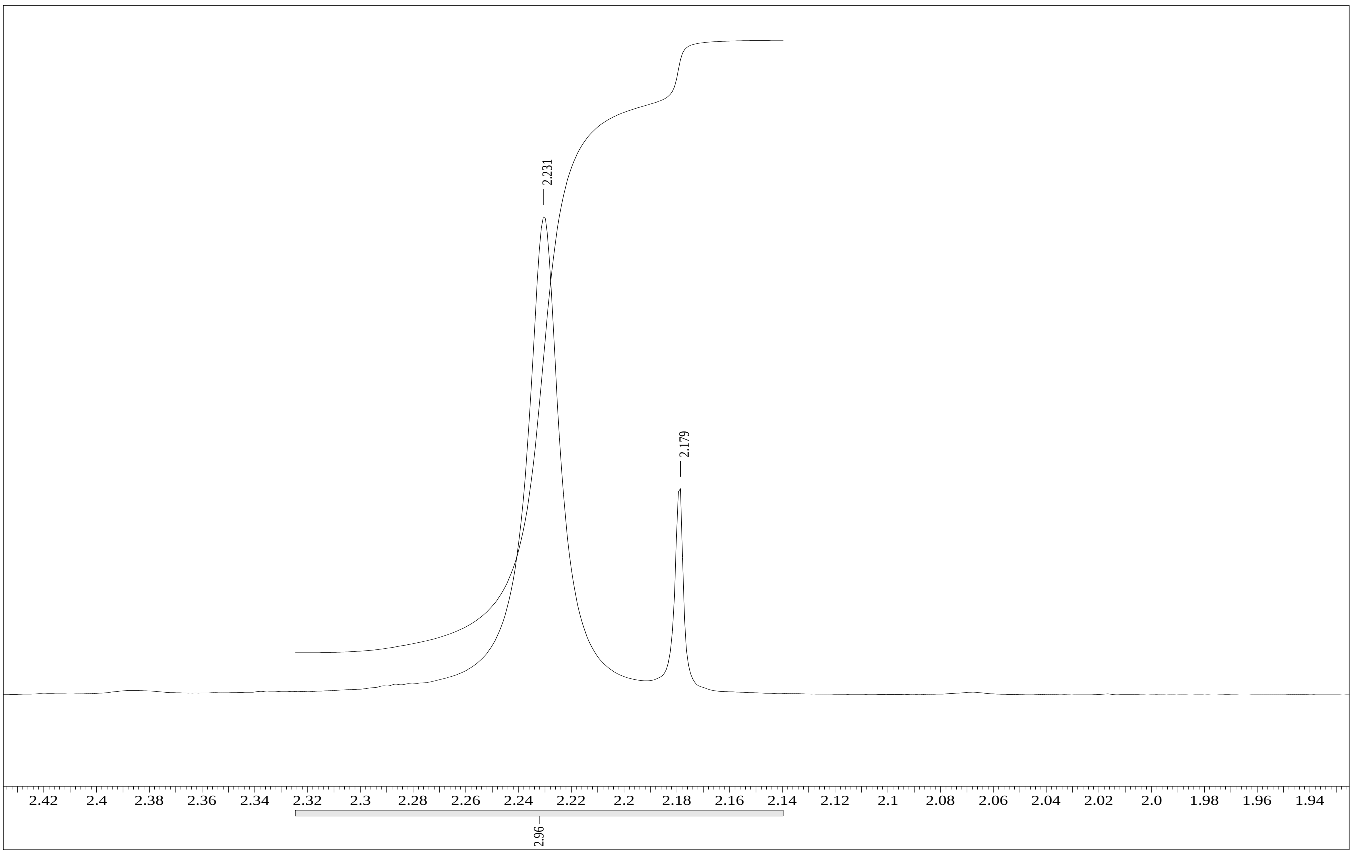

Figure S14. Expanded aliphatic region of ^{1}H NMR spectrum of mixture of (*E*)- and (Z)-2′-methoxyacetophenone thiosemicarbazone (TSC 4)

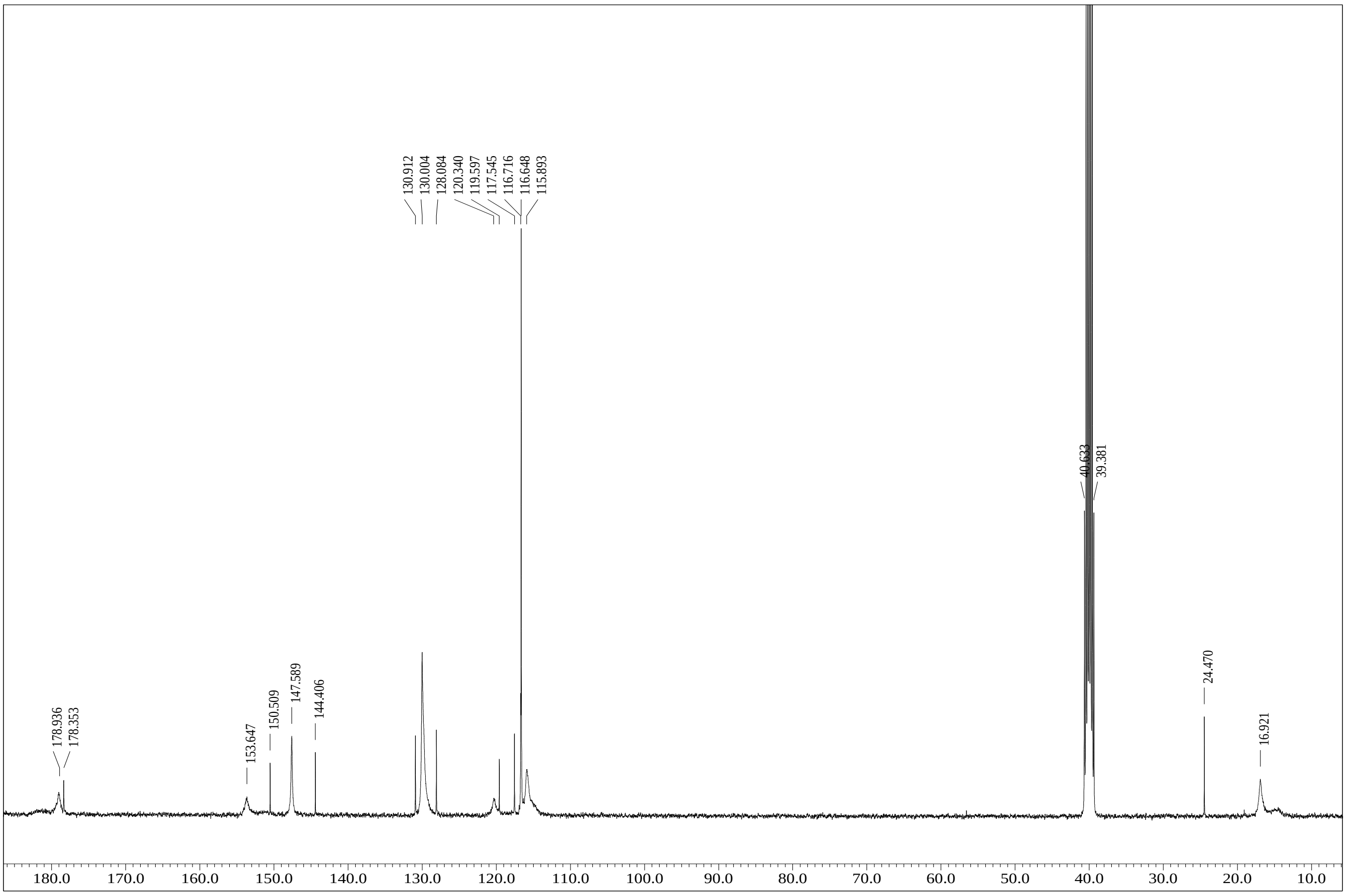

Figure S15. $^{13}C\{^{1}H\}$ NMR spectrum of mixture of (*E*)- and (Z)-2′-aminoacetophenone thiosemicarbazone (TSC 4)

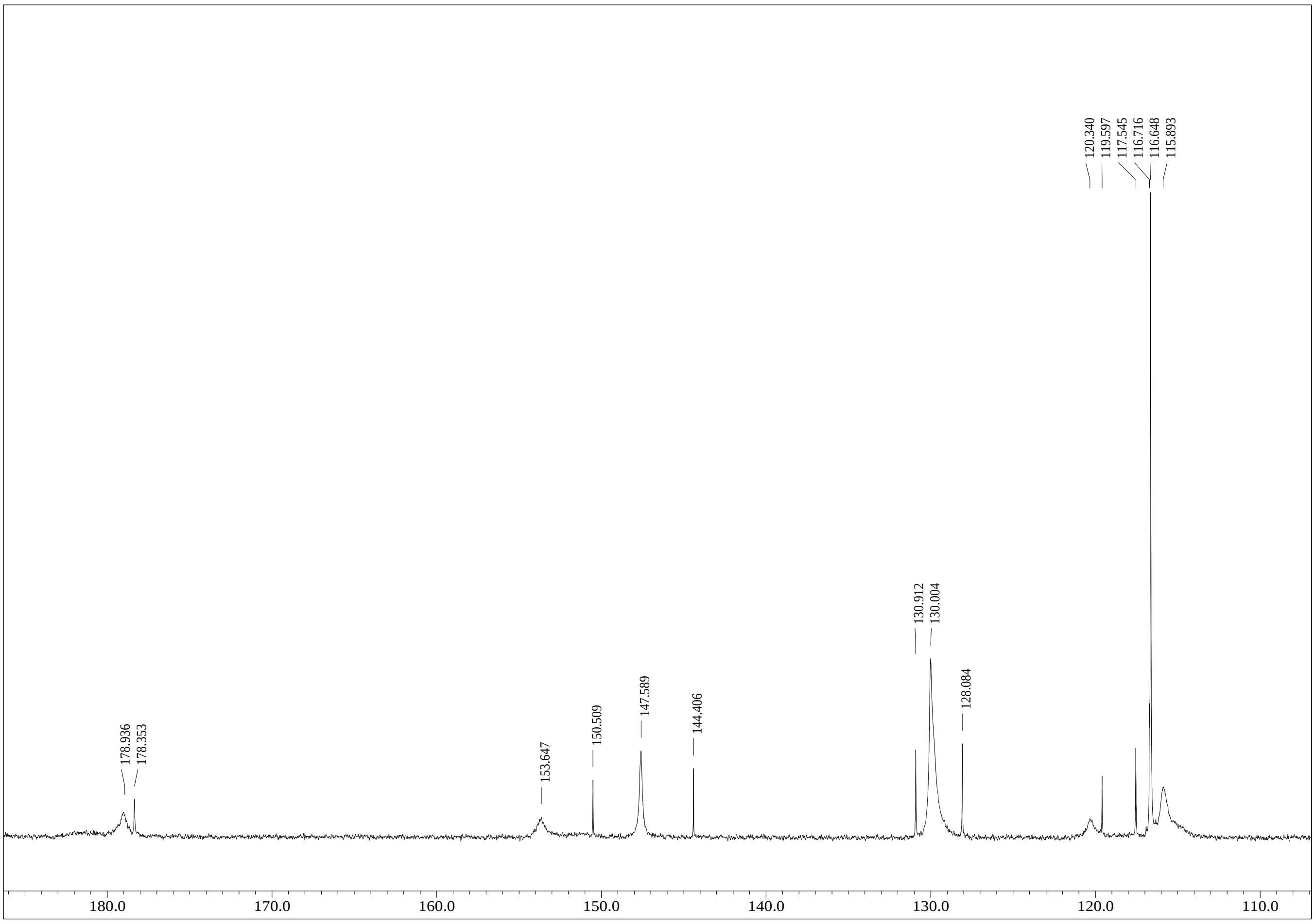

Figure S16. Expanded aromatic region of $^{13}C\{^{1}H\}$ NMR spectrum of mixture of (*E*)- and (Z)-2′-aminoacetophenone thiosemicarbazone (TSC 4)

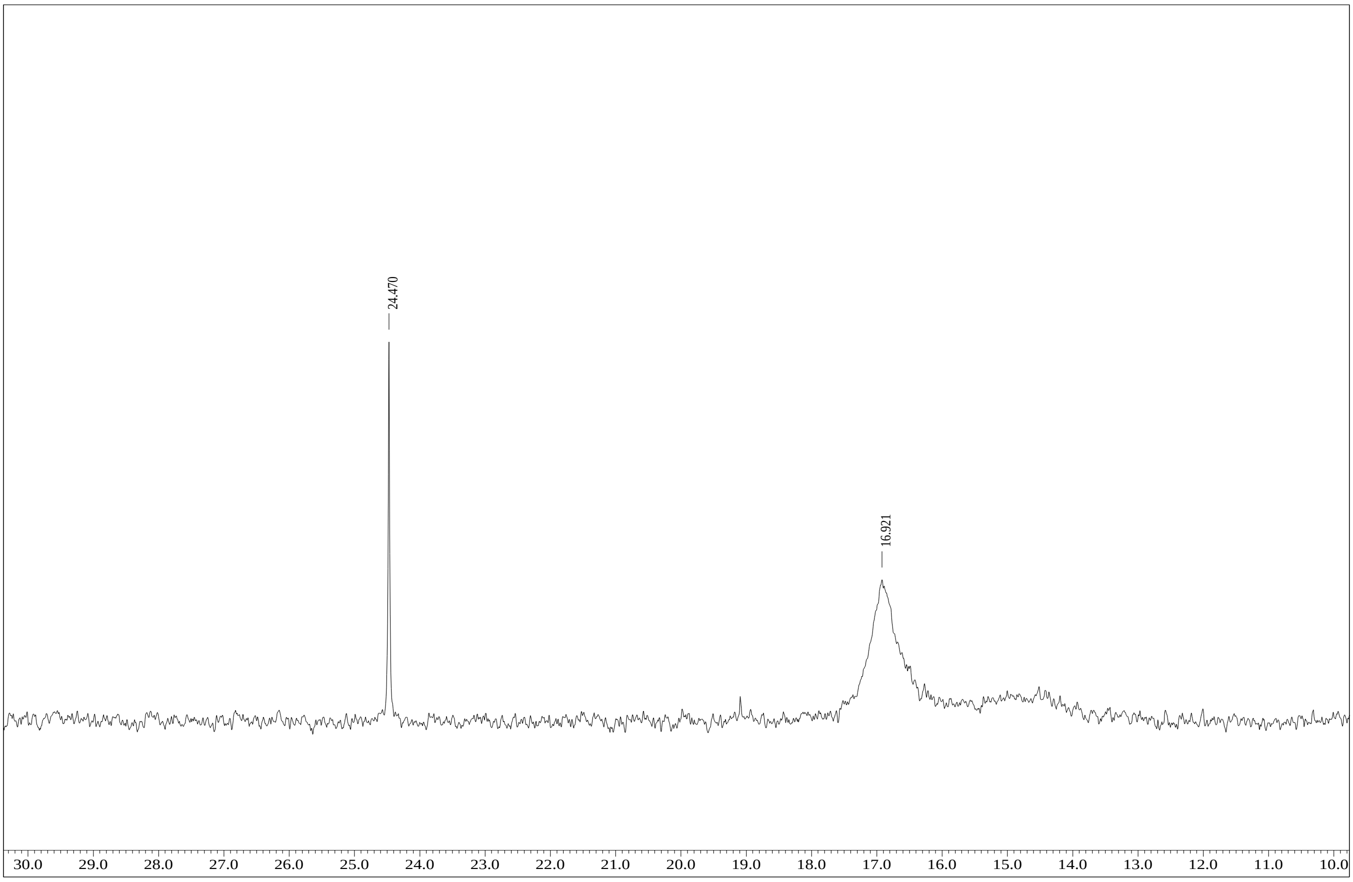

Figure S17. Expanded aliphatic region of $^{13}C\{^{1}H\}$ NMR spectrum of mixture of (*E*)- and (Z)-2′-aminoacetophenone thiosemicarbazone (TSC 4)

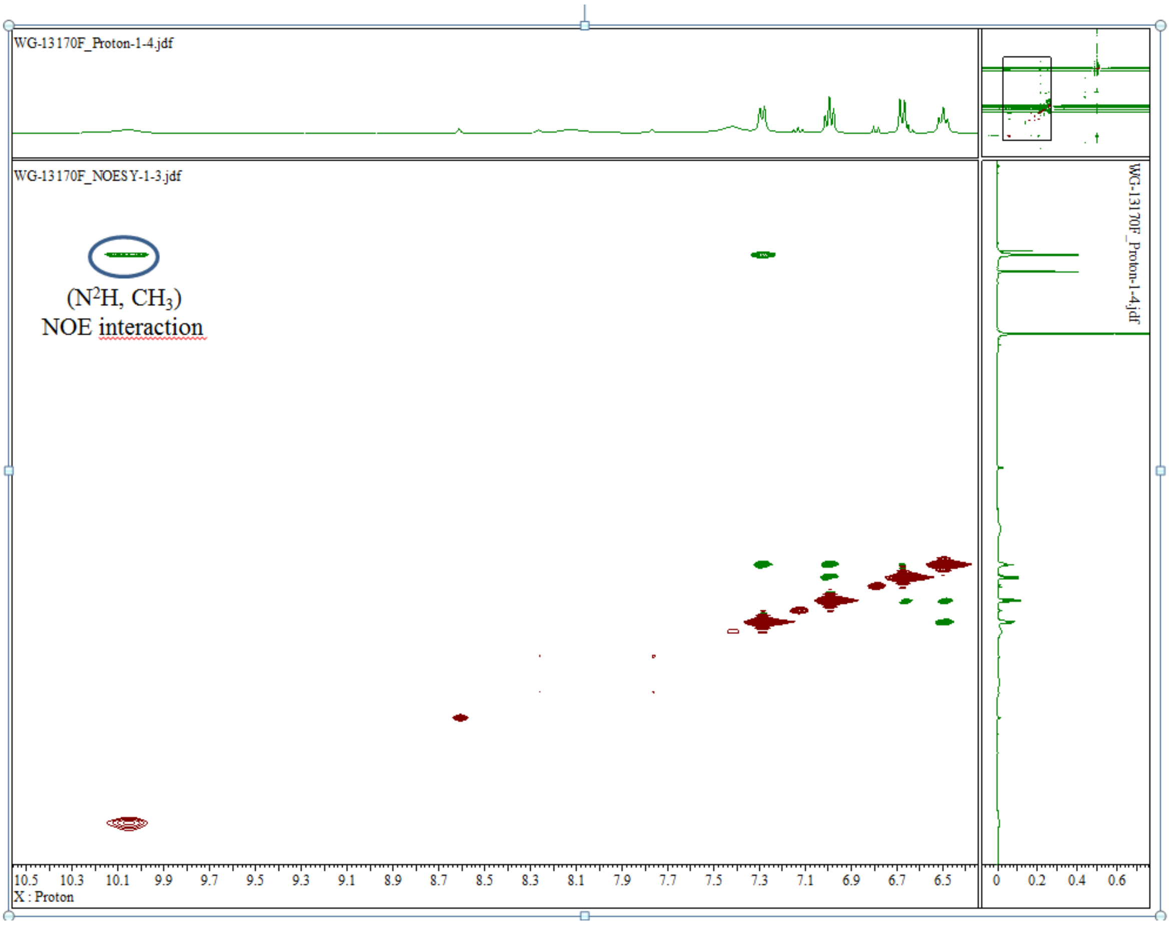

Figure S18. NOESY spectrum of the mixture of (*E*)- and (Z)-2′-aminoacetophenone thiosemicarbazone (TSC 4)

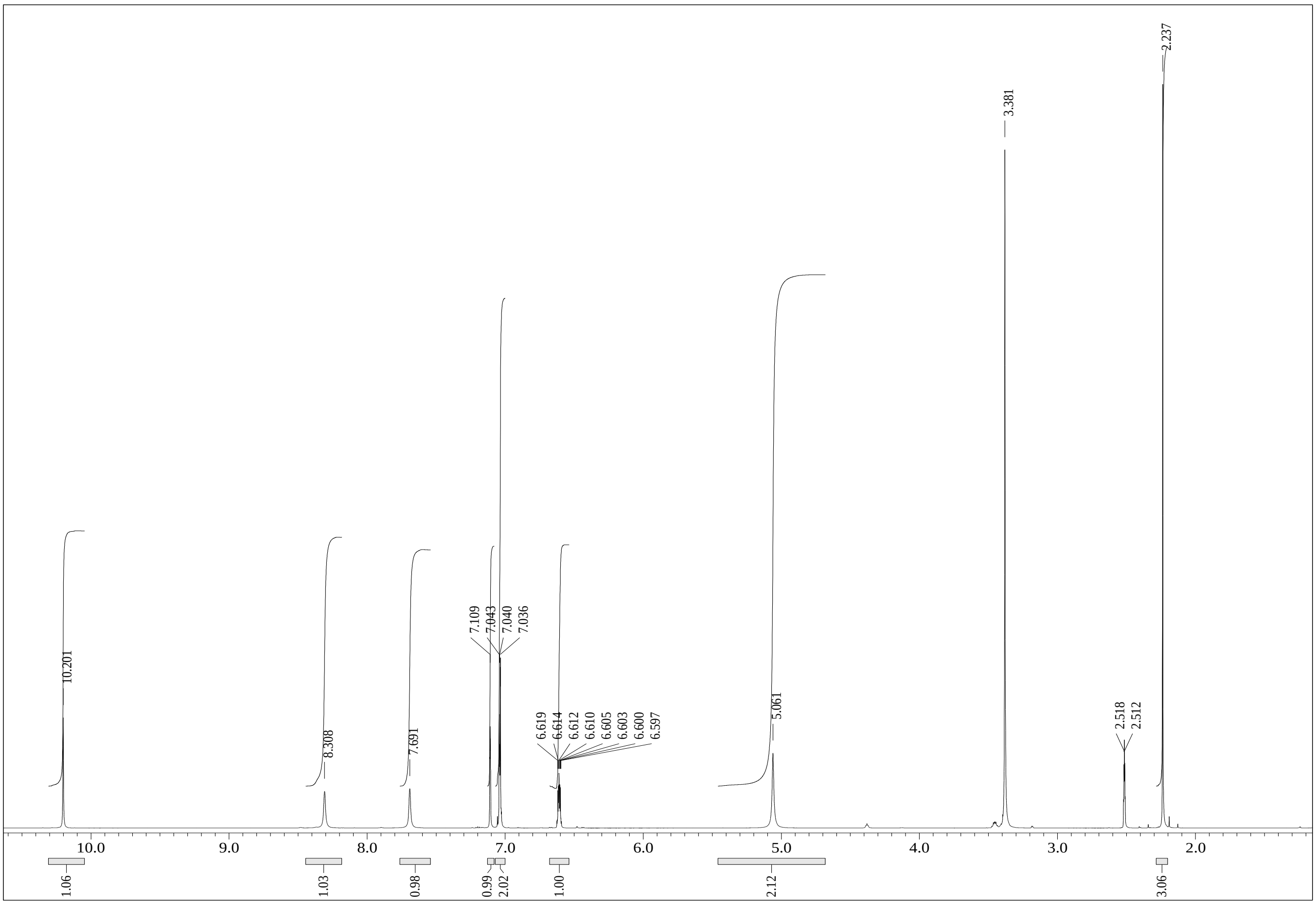

Figure S19. ^{1}H NMR spectrum of mixture of (*E*)- 3′-aminoacetophenone thiosemicarbazone (TSC 5)

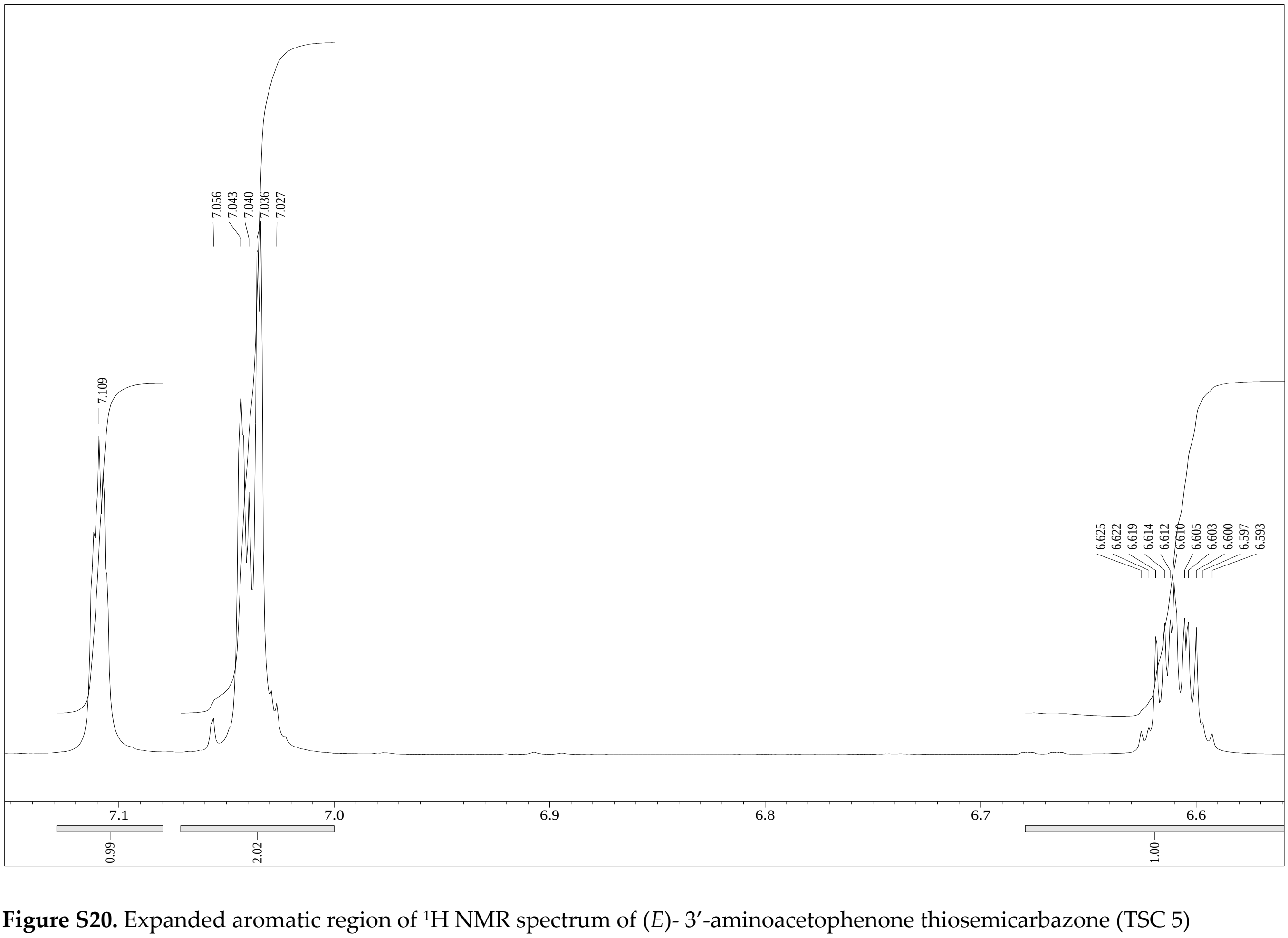

Figure S20. Expanded aromatic region of ^{1}H NMR spectrum of (*E*)- 3′-aminoacetophenone thiosemicarbazone (TSC 5)

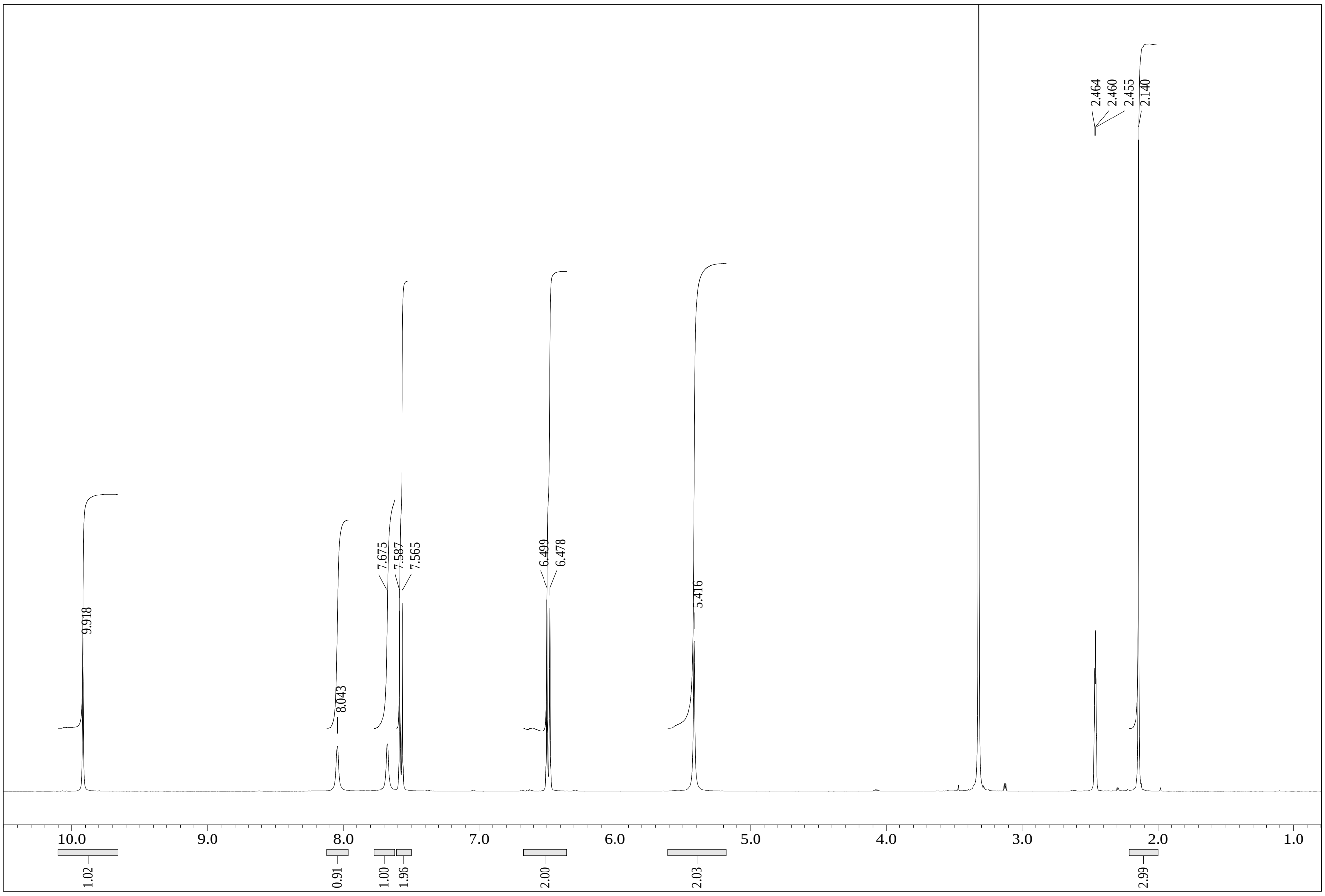

Figure S21. ^{1}H NMR spectrum of mixture of (*E*)- 4′-aminoacetophenone thiosemicarbazone (TSC 6)

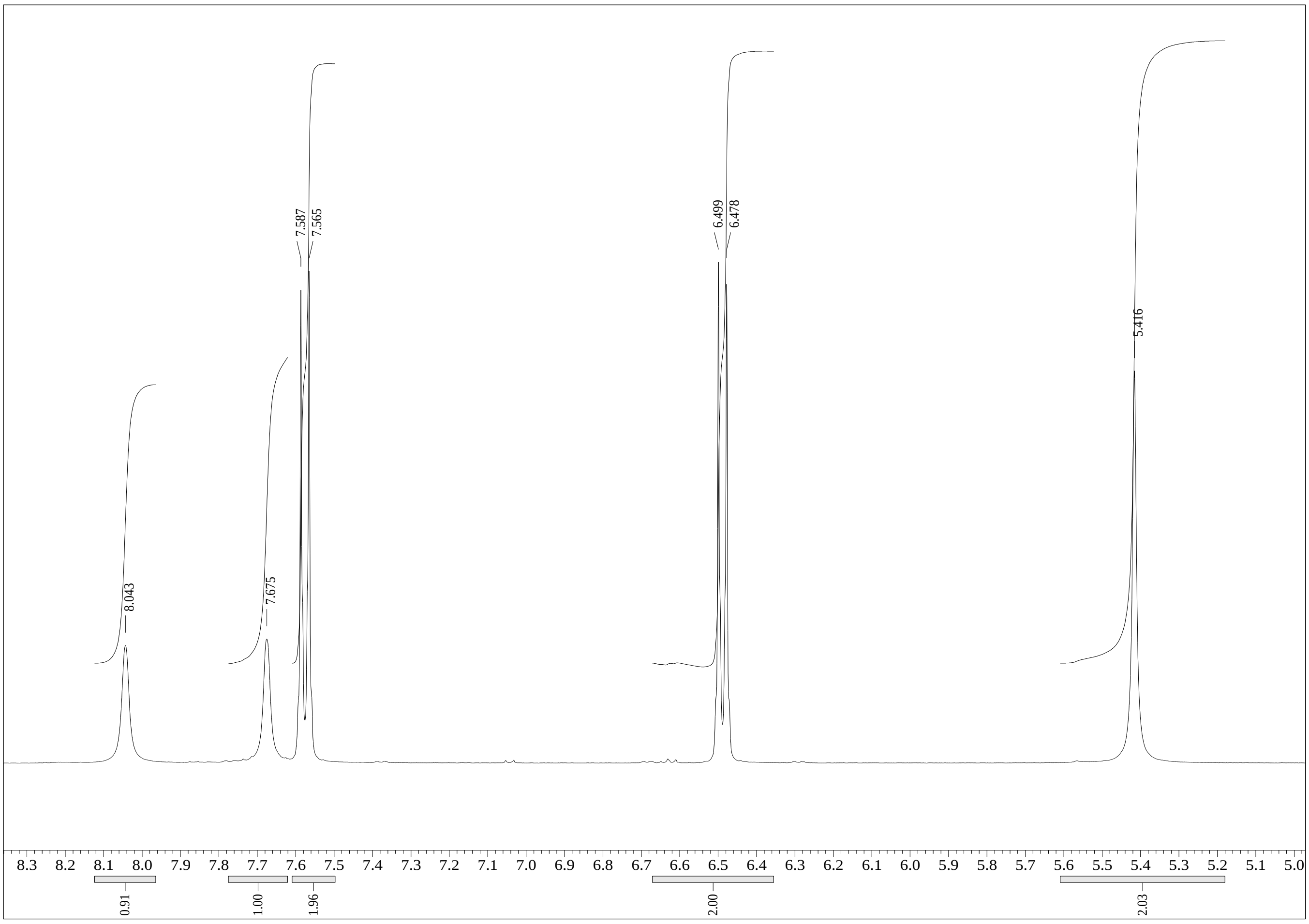

Figure S22. Expanded aromatic region of ^{1}H NMR spectrum of (*E*)- 4′-aminoacetophenone thiosemicarbazone (TSC 6)

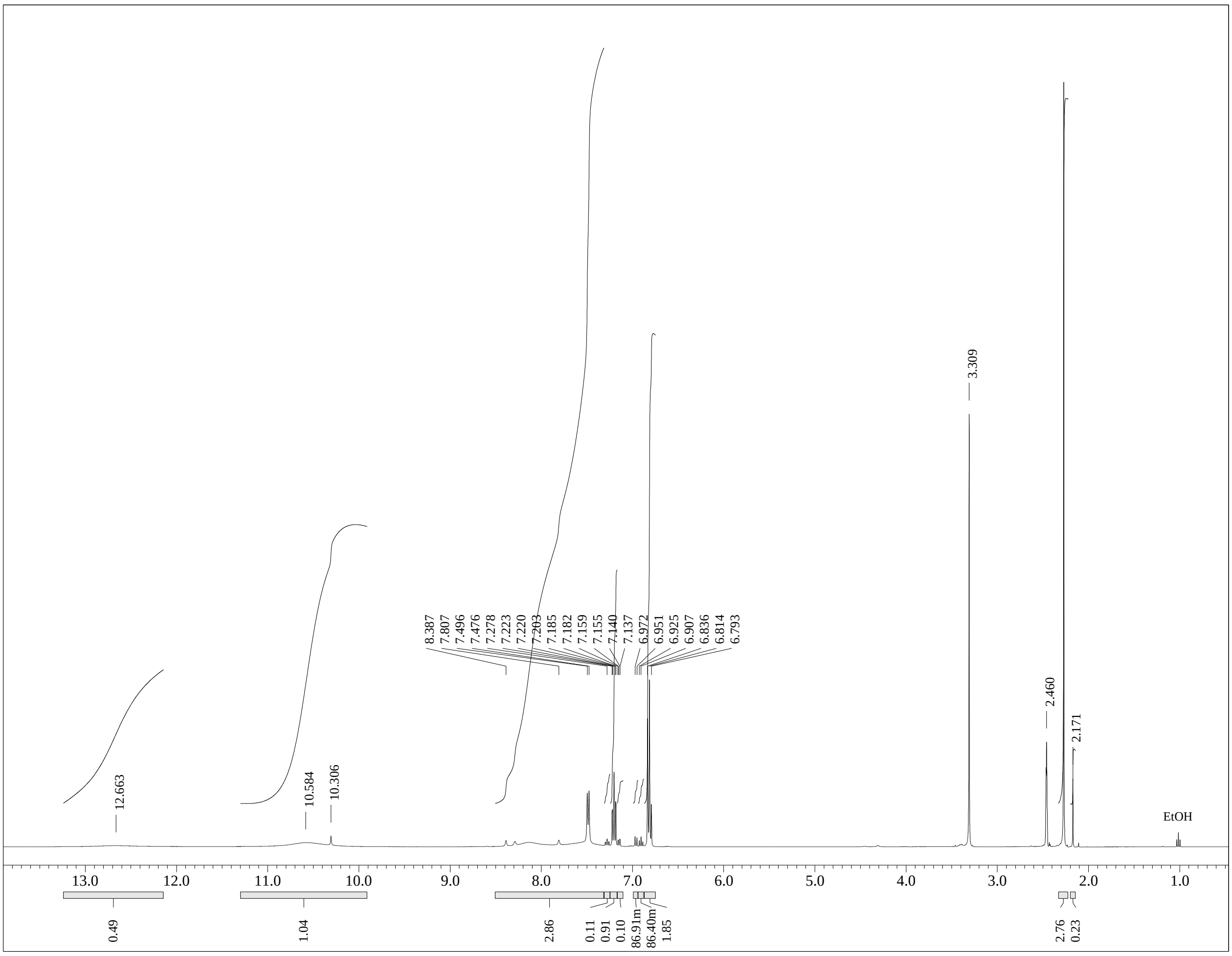

Figure S23. ^{1}H NMR spectrum of mixture of (*E*)- and (Z)-2′-hydroxyacetophenone thiosemicarbazone (TSC 7)

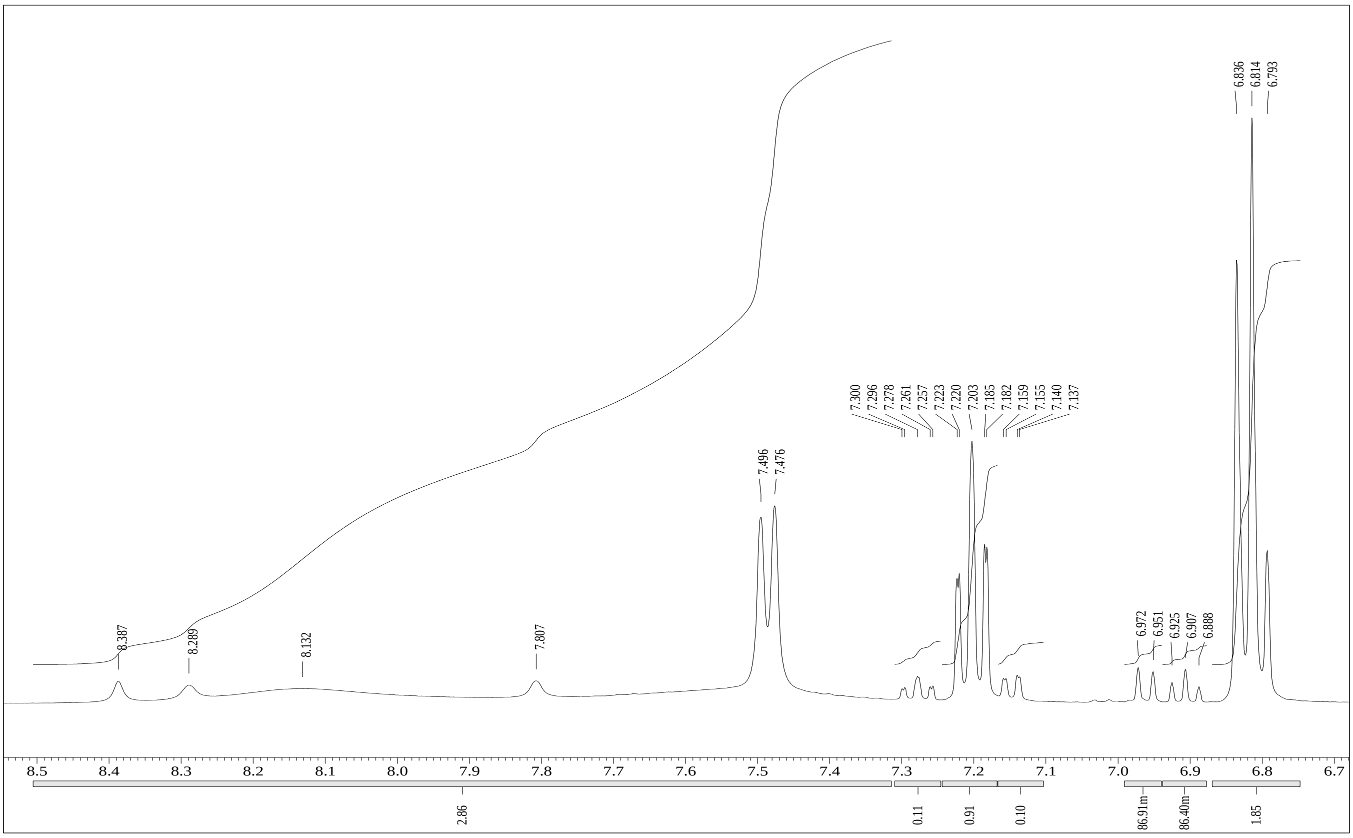

Figure S24. Expanded aromatic region of ^{1}H NMR spectrum of mixture of (*E*)- and (Z)-2′-hydroxyacetophenone thiosemicarbazone (TSC 7)

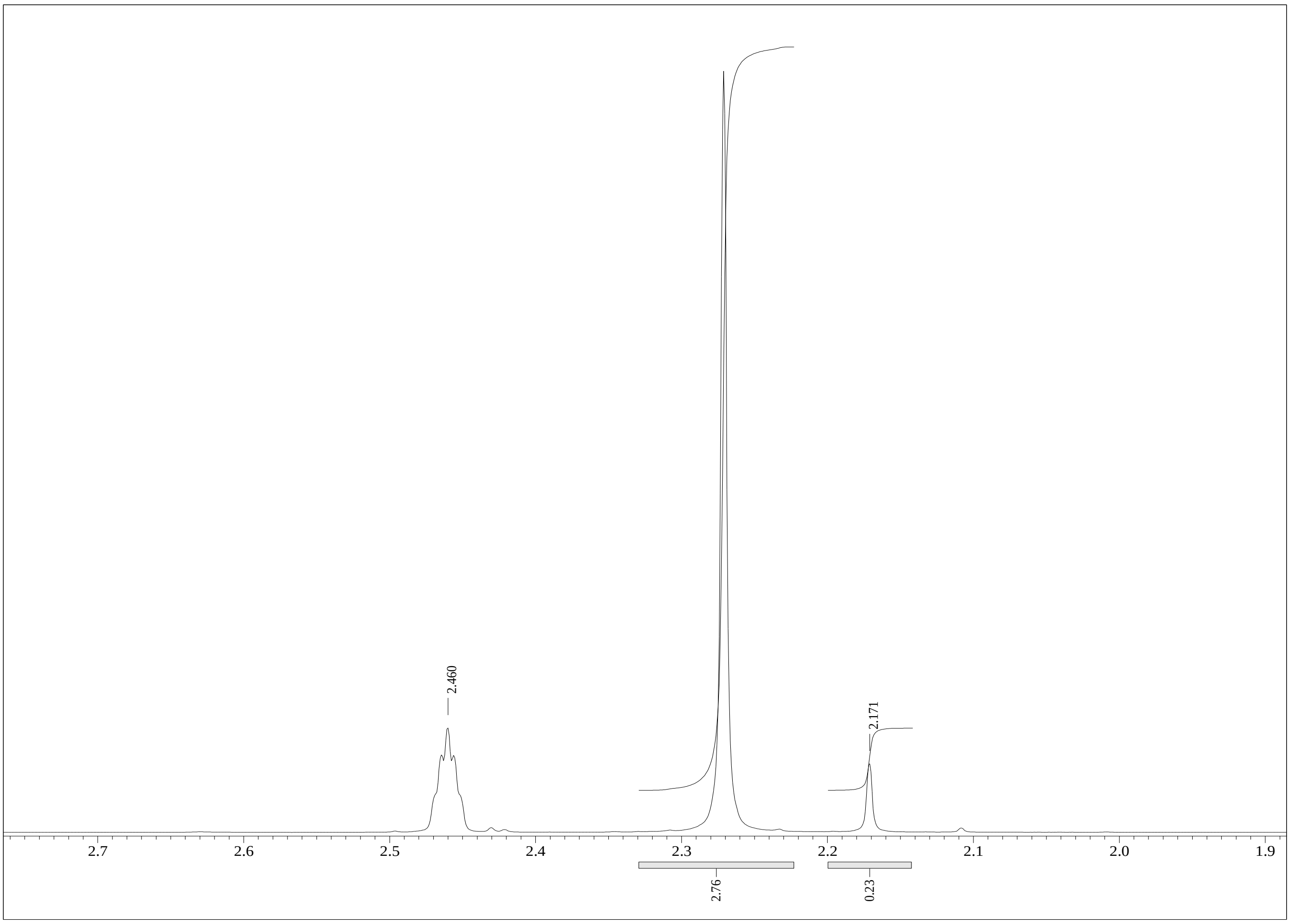

Figure S25. Expanded aliphatic region of ^{1}H NMR spectrum of mixture of (*E*)- and (Z)-2′-hydroxyacetophenone thiosemicarbazone (TSC 7)

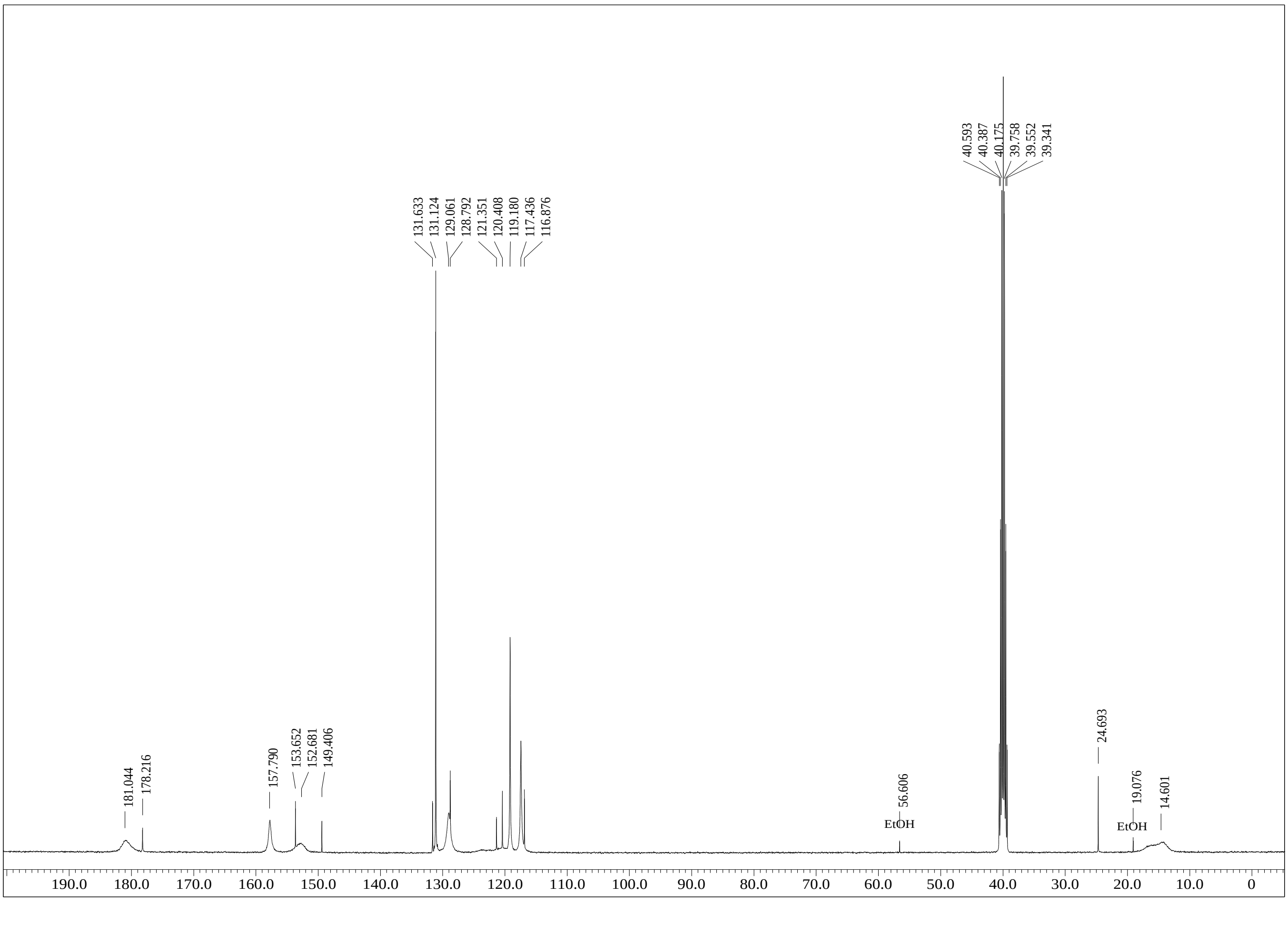

Figure S26. ^{13}C NMR spectrum of mixture of (*E*)- and (Z)-2′-hydroxyacetophenone thiosemicarbazone (TSC 7)

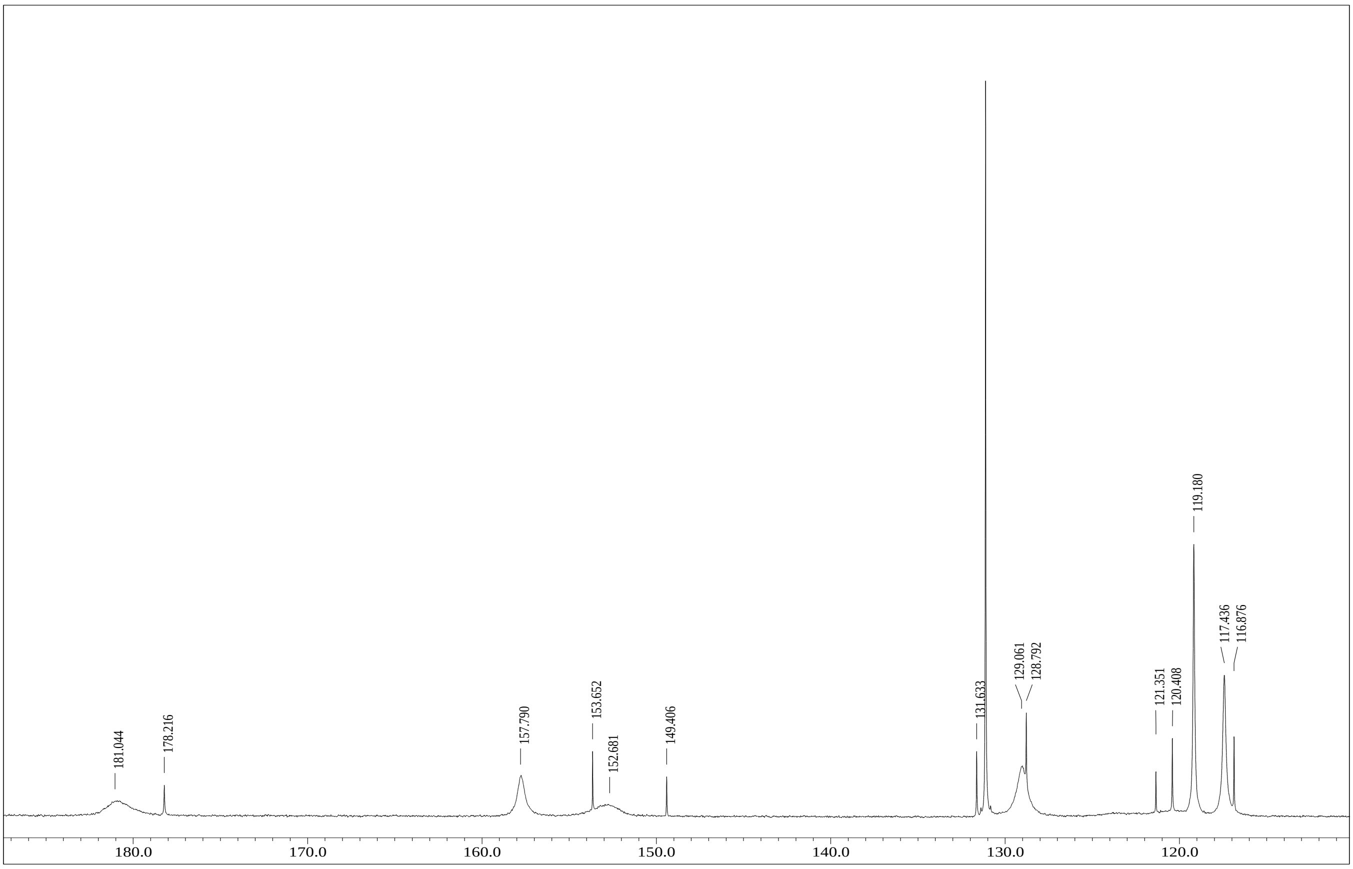

Figure S27. Expanded aromatic region of ^{13}C NMR spectrum of mixture of (*E*)- and (Z)-2′-hydroxyacetophenone thiosemicarbazone (TSC 7)

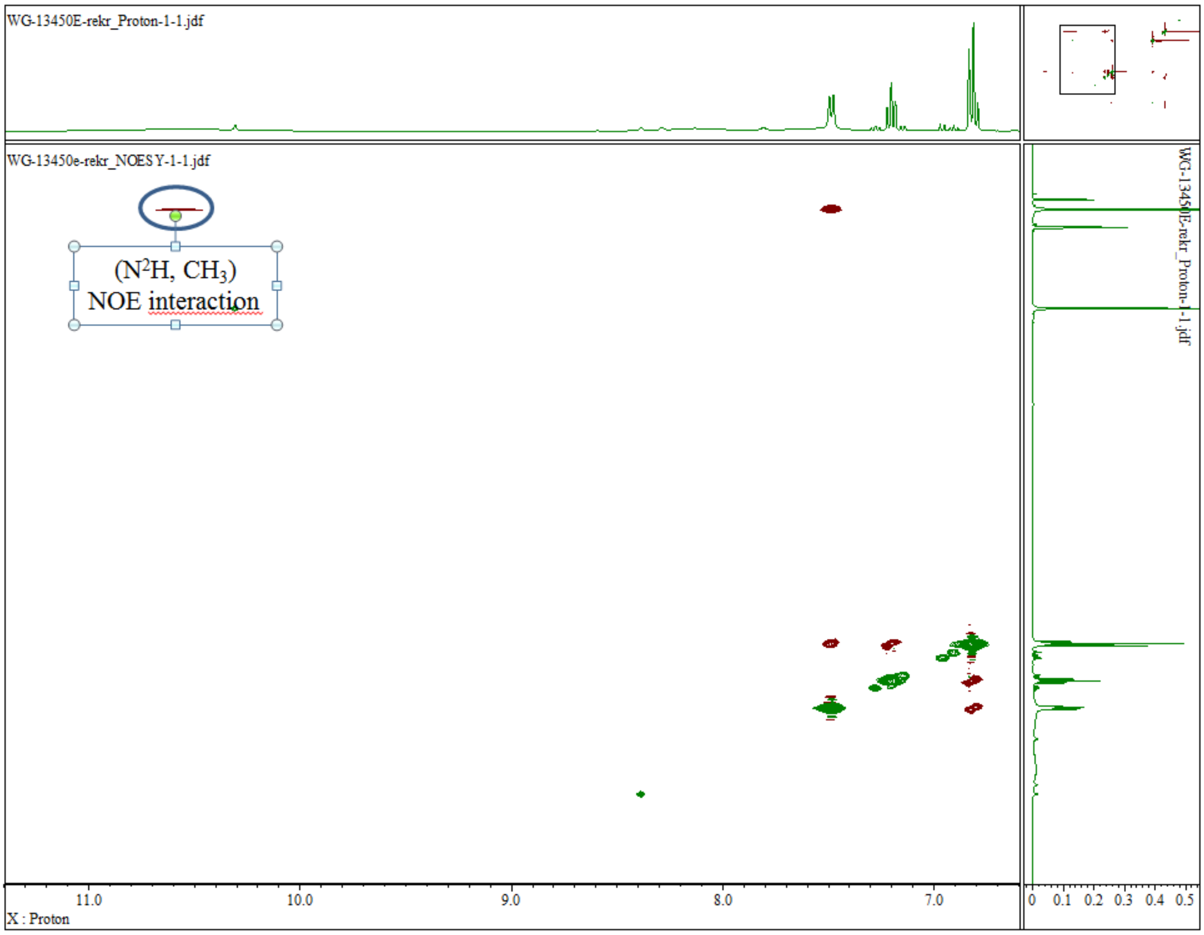

Figure S28. NOESY spectrum of mixture of (*E*)- and (Z)-2′-hydroxyacetophenone thiosemicarbazone (TSC 7)

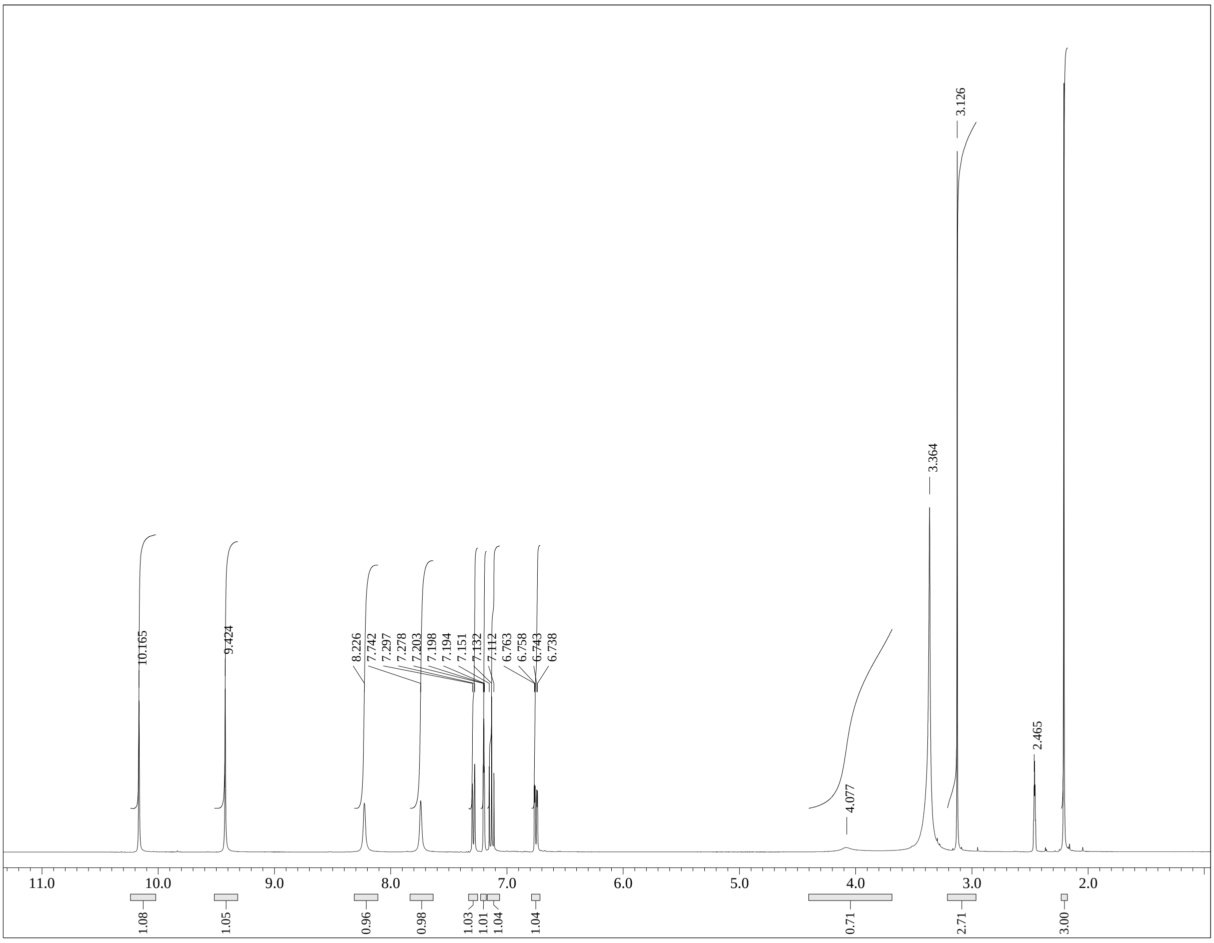

Figure S29. ^{1}H NMR spectrum of methanol solvate of (*E*)- 3′-hydroxyacetophenone thiosemicarbazone (TSC 8 • CH_3OH)

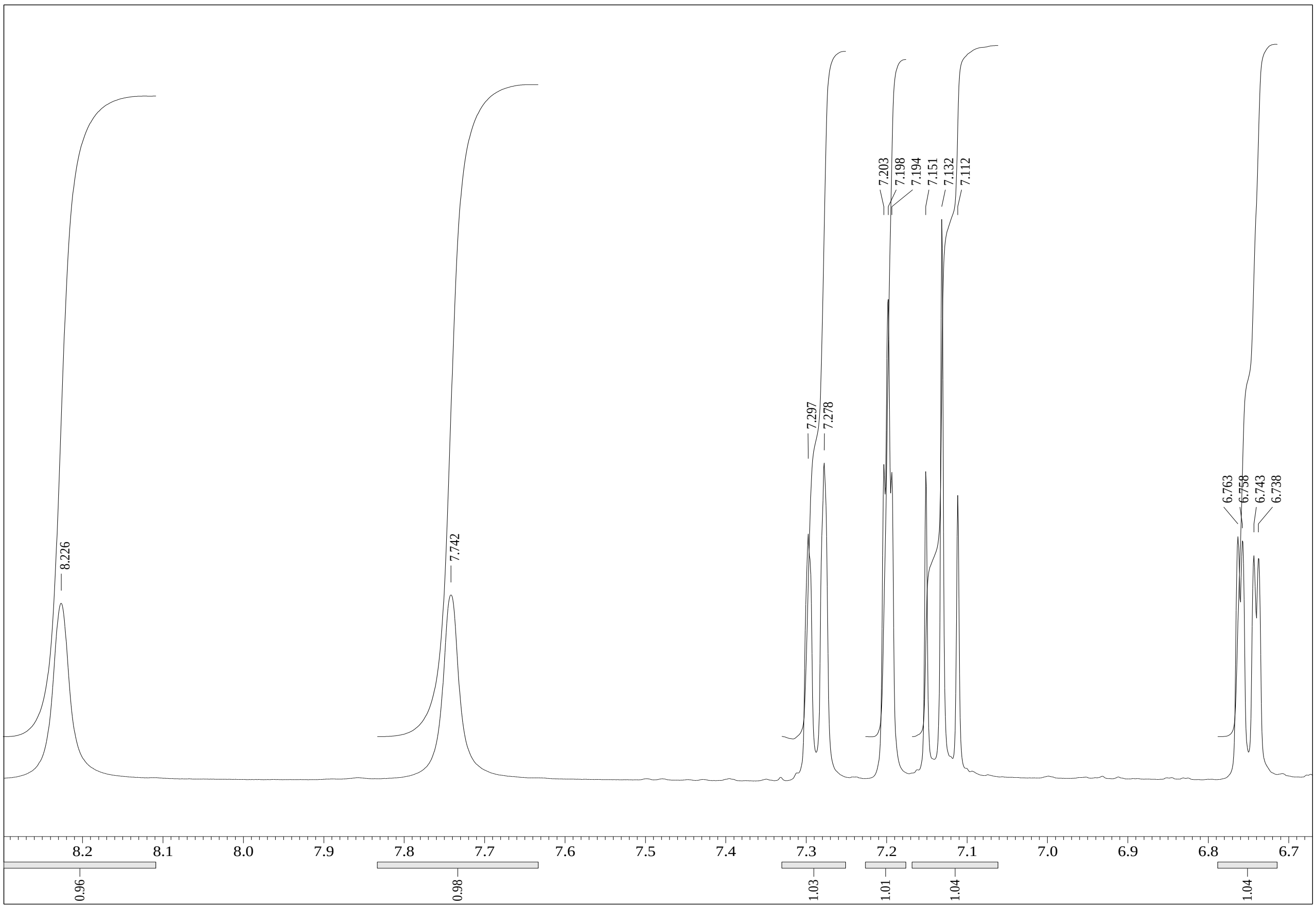

Figure S30. Expanded aromatic region of ^{1}H NMR spectrum of methanol solvate of (*E*)- 3′-hydroxyacetophenone thiosemicarbazone (TSC 8 • CH_3OH)

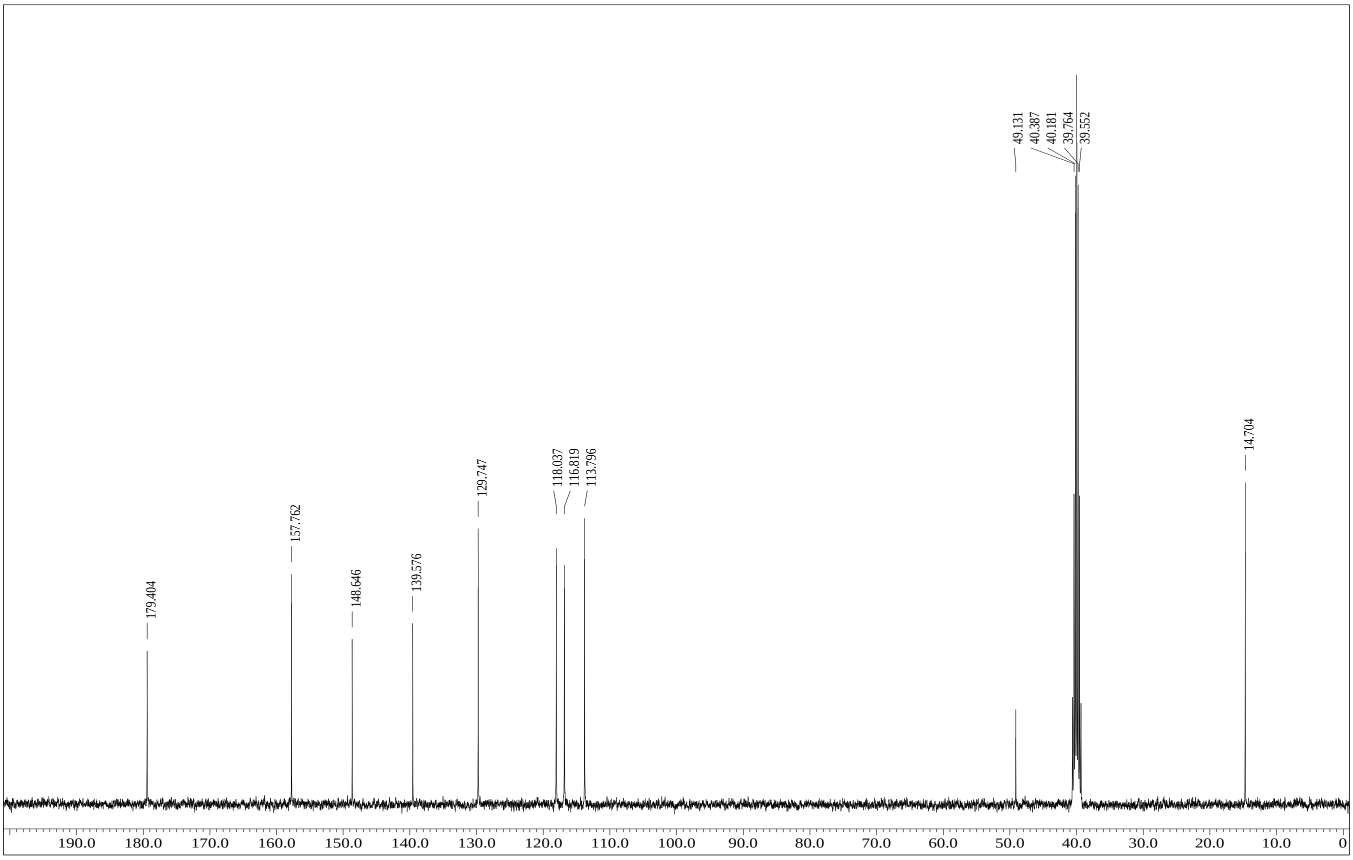

Figure S31. ^{13}C NMR spectrum of methanol solvate of (*E*)- 3′-hydroxyacetophenone thiosemicarbazone (TSC 8 • CH_3OH)

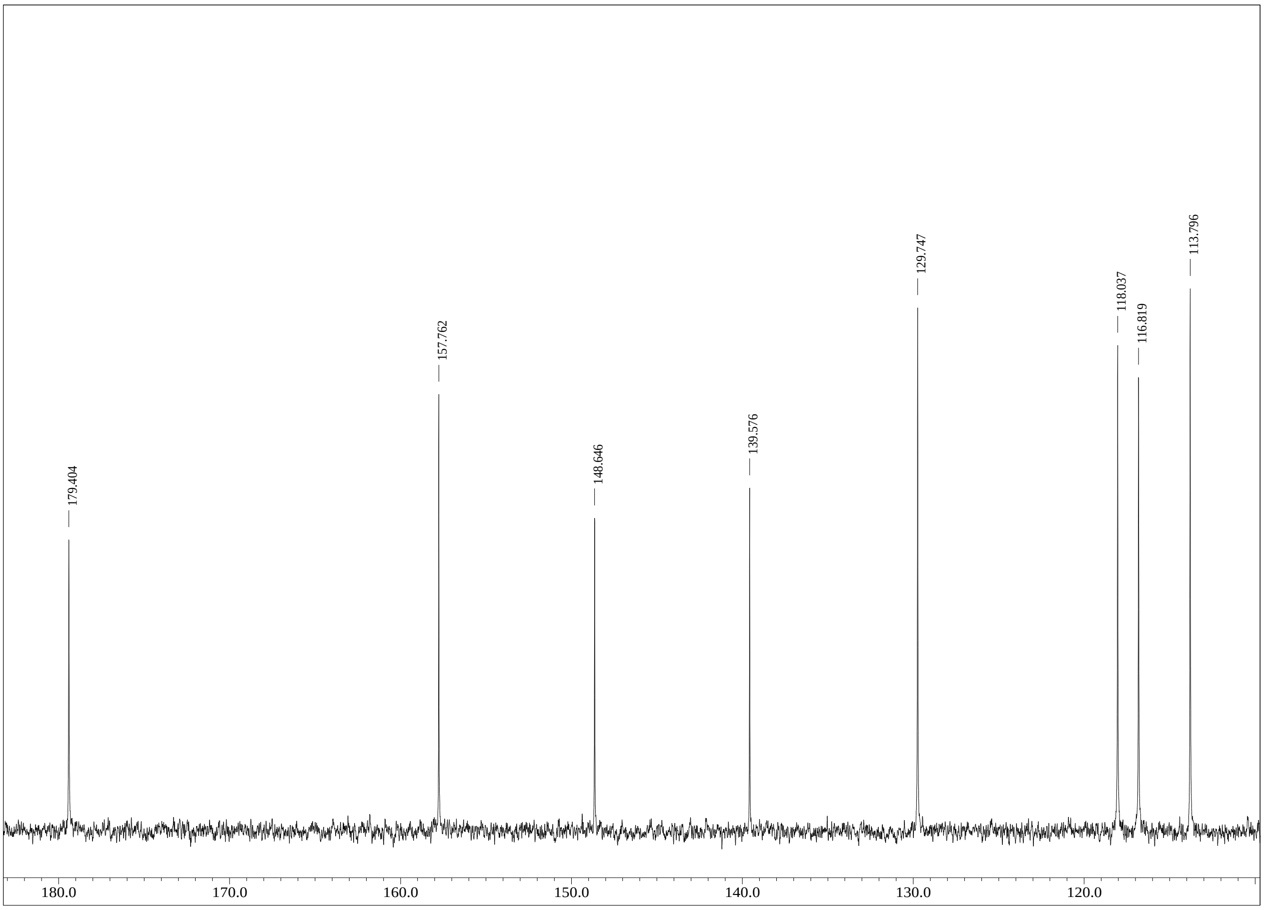

Figure S32. Expanded aromatic region of ^{13}C NMR spectrum of methanol solvate of (*E*)- 3′-hydroxyacetophenone thiosemicarbazone (TSC 8 • CH_3OH)

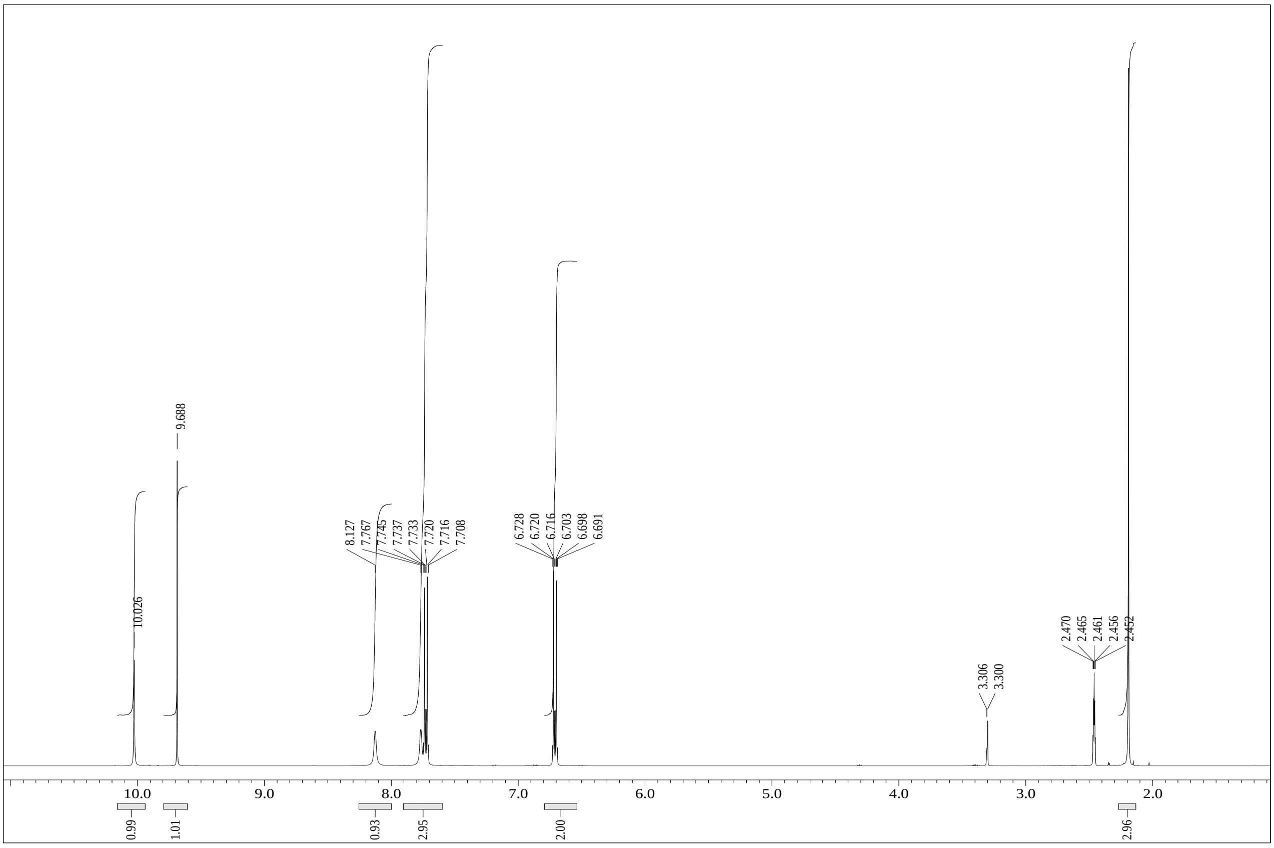

Figure S33. ^{1}H NMR spectrum of (*E*)- 4′-hydroxyacetophenone thiosemicarbazone (TSC 9)

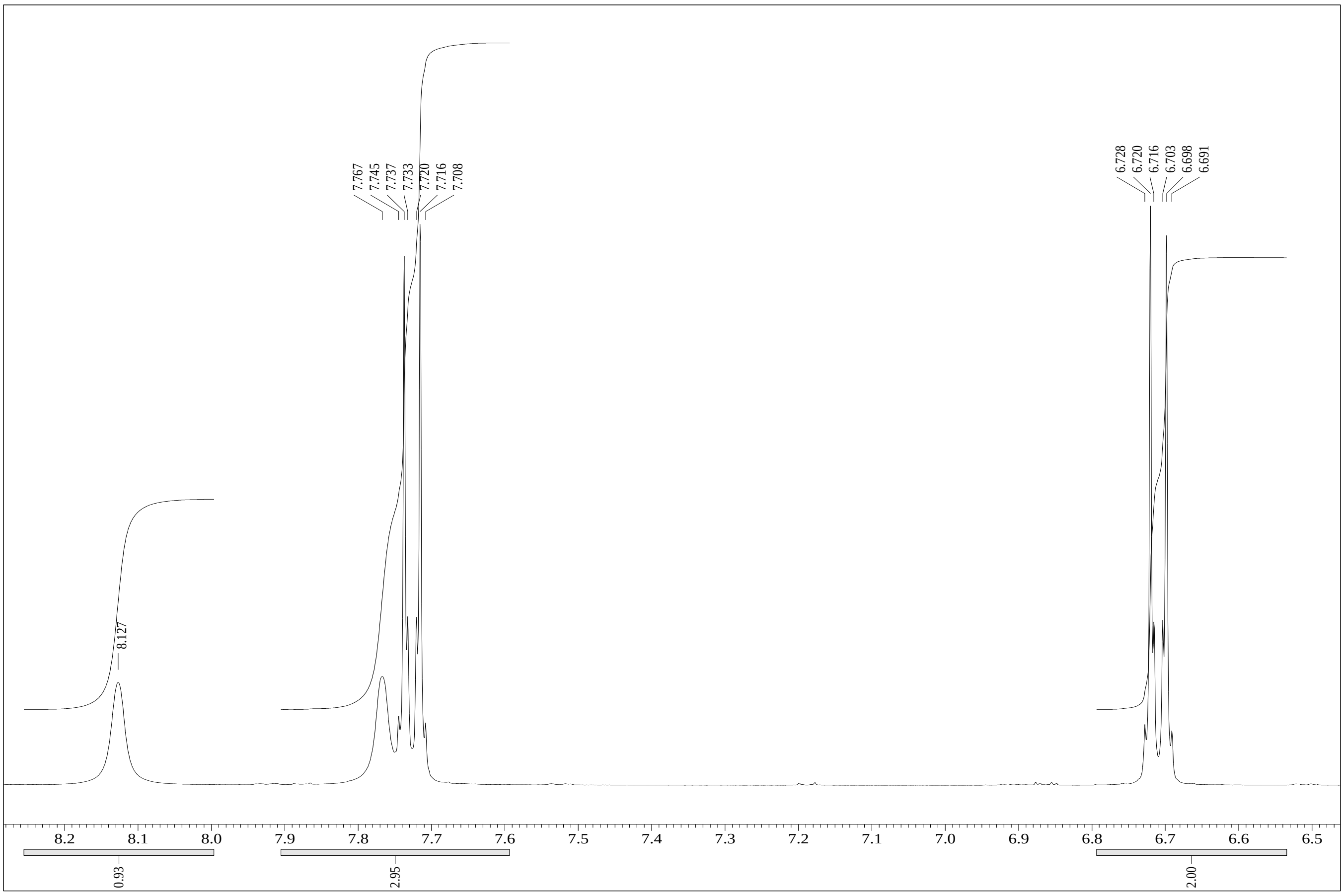

Figure S34. Expanded aromatic region of ^{1}H NMR spectrum of (*E*)- 4′-hydroxyacetophenone thiosemicarbazone (TSC 9)

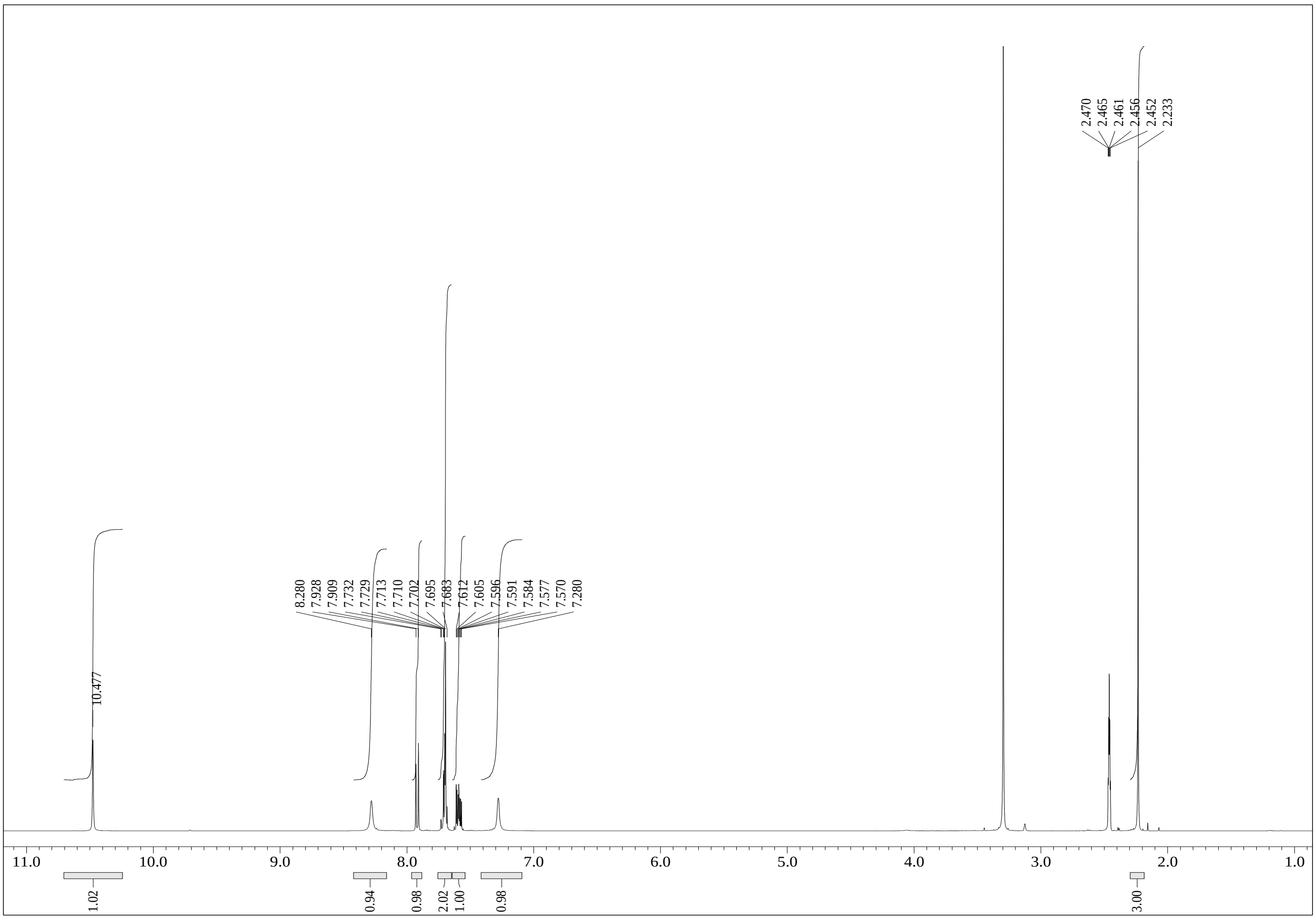

Figure S35. ^{1}H NMR spectrum of (*E*)- 2′-nitroacetophenone thiosemicarbazone (TSC 10)

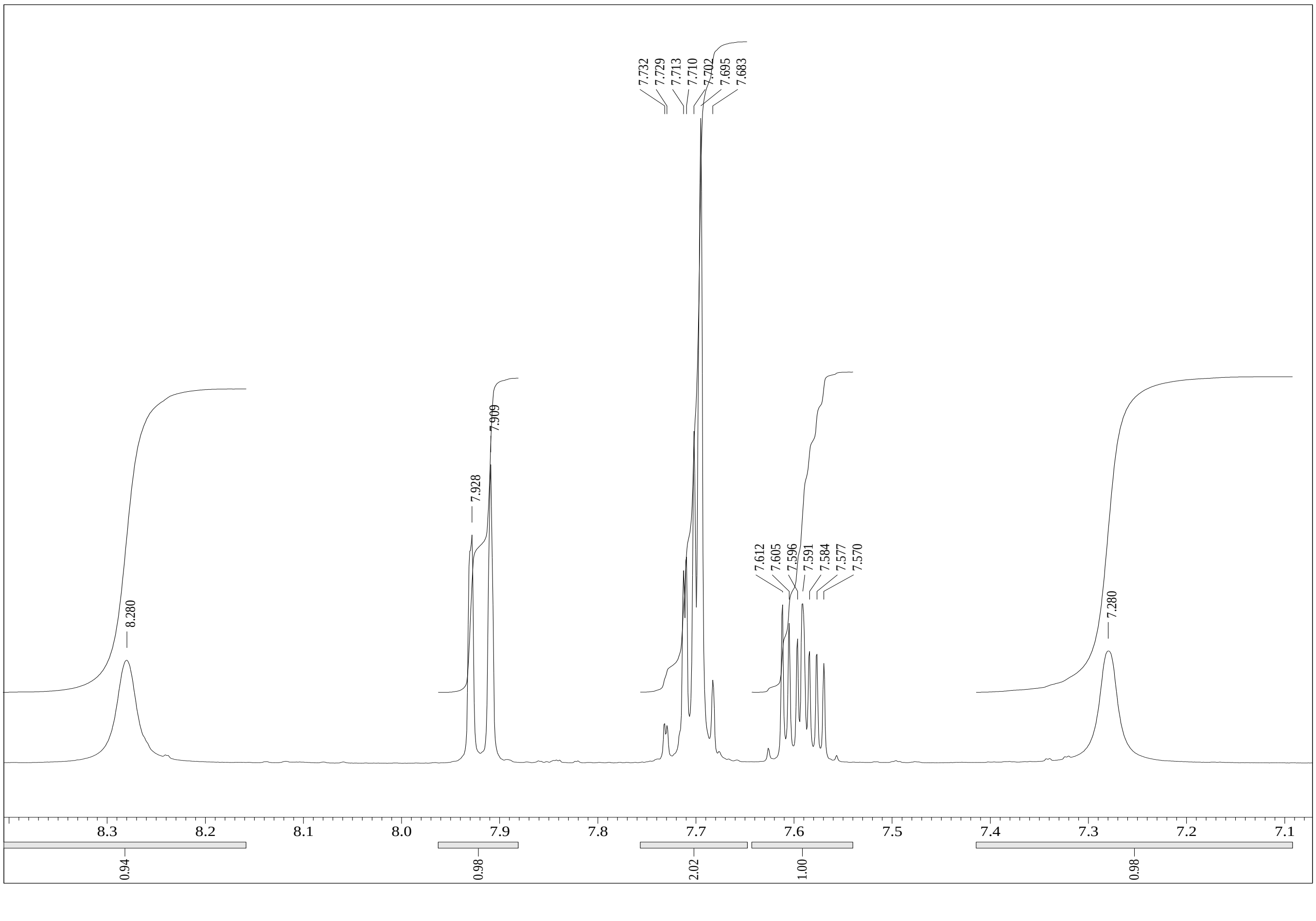

Figure S36. Expanded aromatic region of ^{1}H NMR spectrum of (*E*)- 2′-nitroacetophenone thiosemicarbazone (TSC 10)

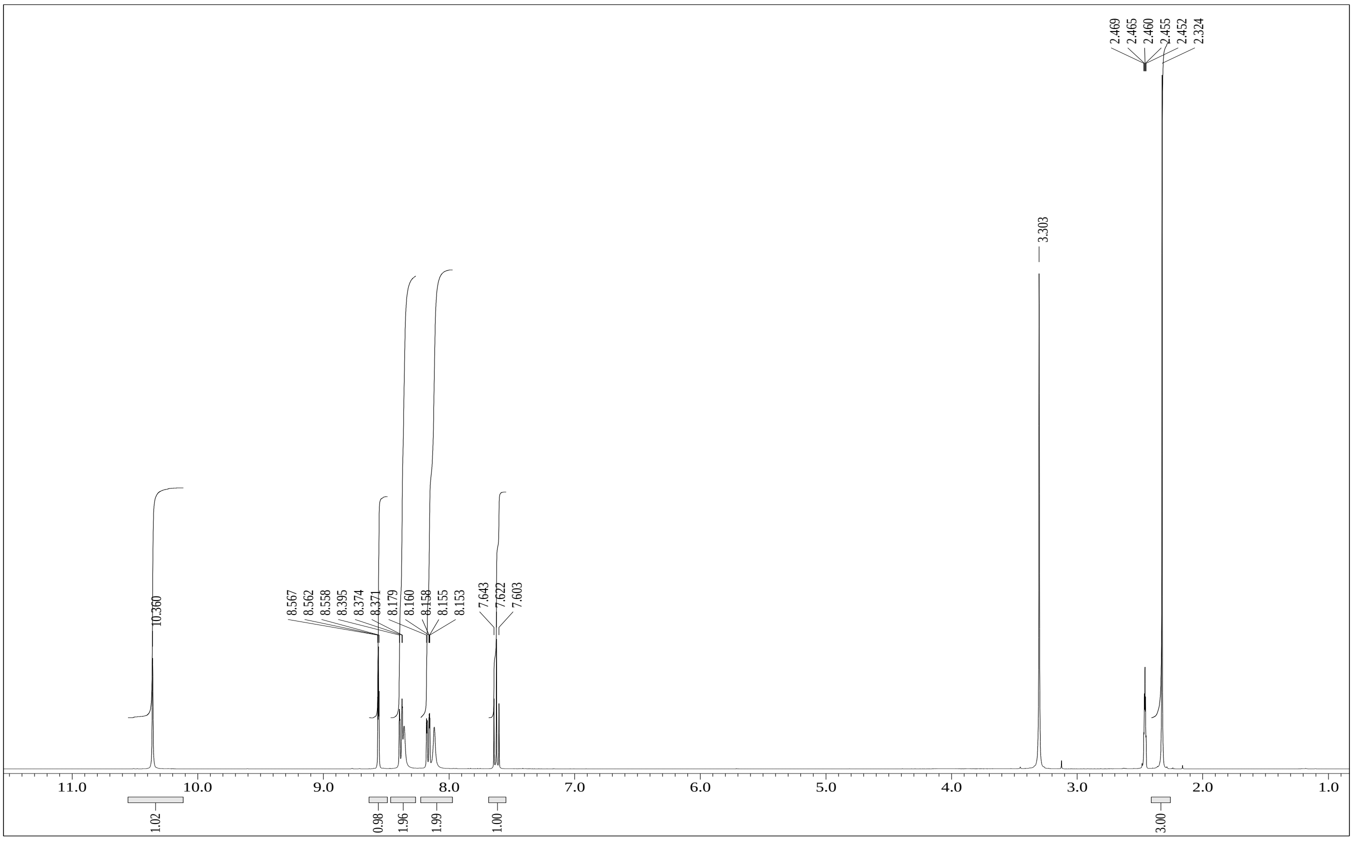

Figure S37. ^{1}H NMR spectrum of (*E*)- 3′-nitroacetophenone thiosemicarbazone (TSC 11)

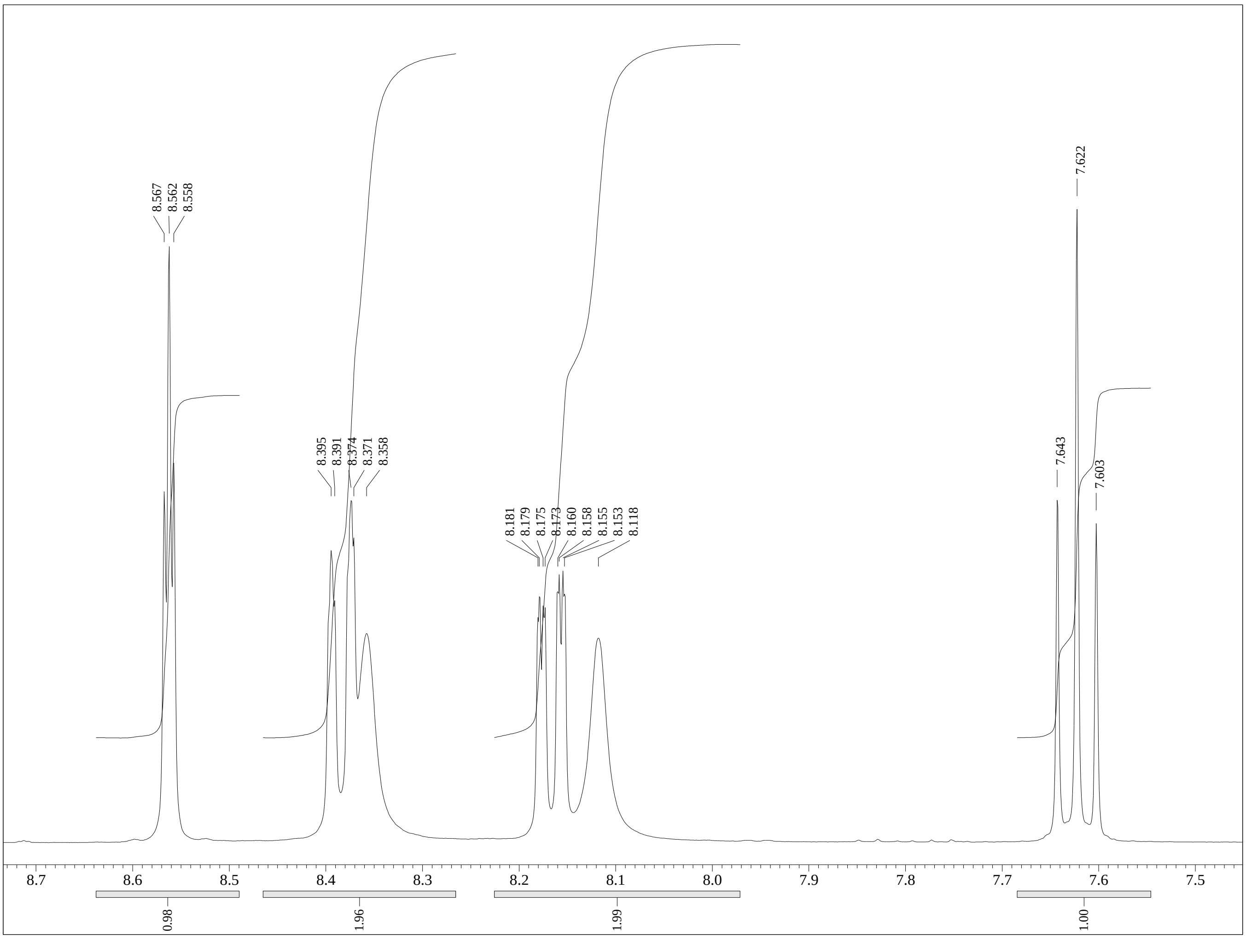

Figure S38. Expanded aromatic region of ^{1}H NMR spectrum of (*E*)- 3′-nitroacetophenone thiosemicarbazone (TSC 11)

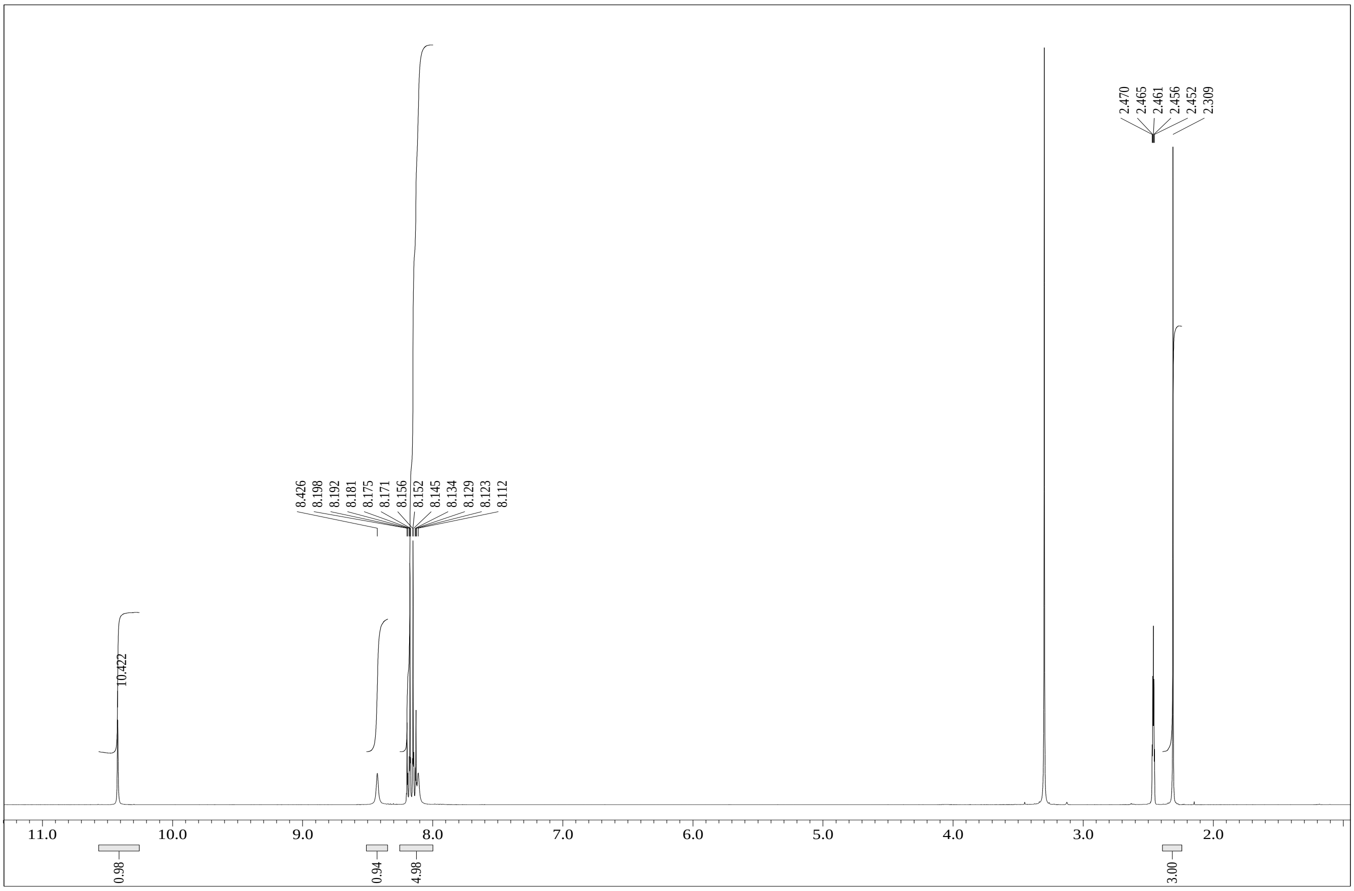

Figure S39. ^{1}H NMR spectrum of (*E*)- 4′-nitroacetophenone thiosemicarbazone (TSC 12)

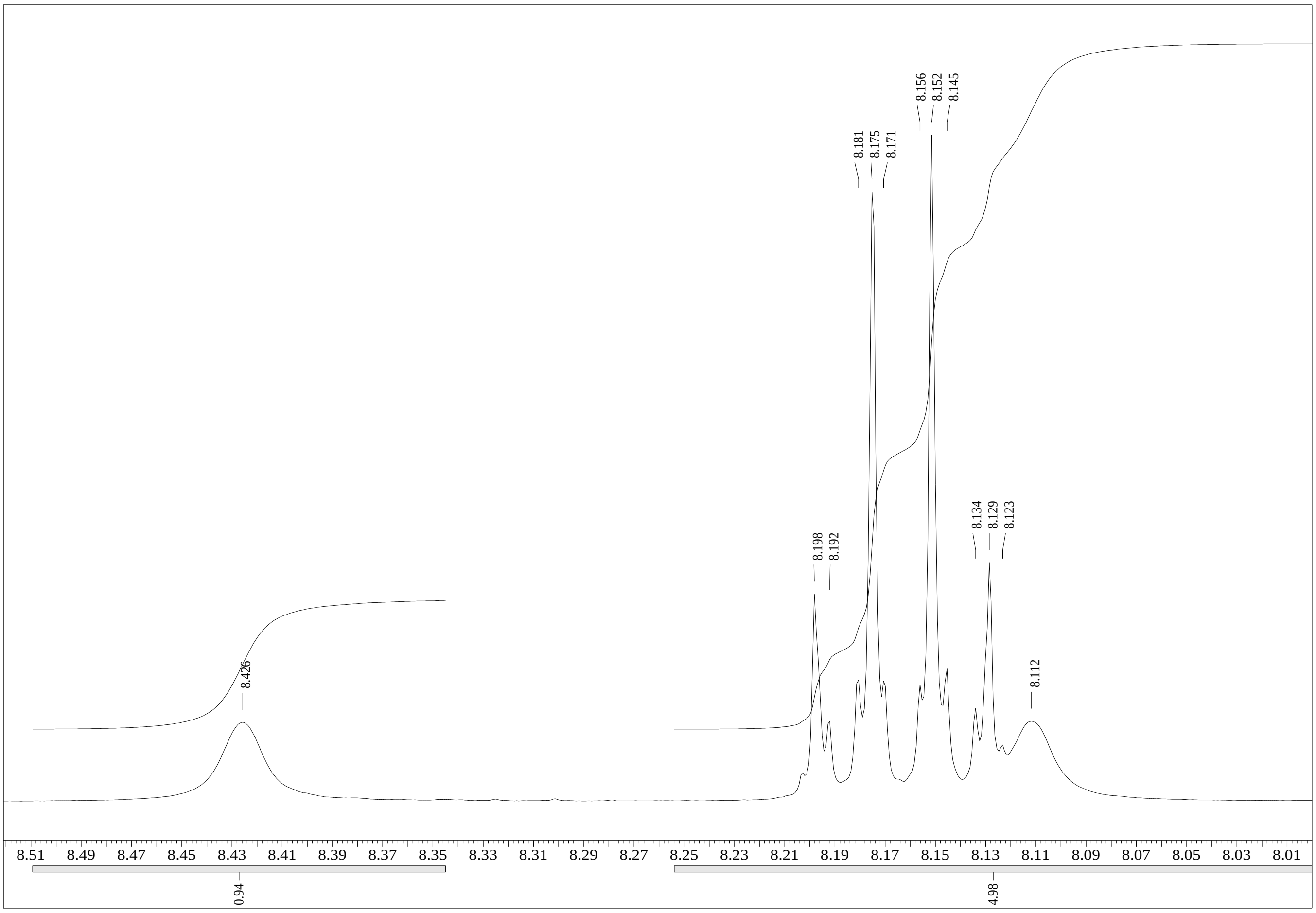

Figure S40. Expanded aromatic region of ^{1}H NMR spectrum of (*E*)- 4′-nitroacetophenone thiosemicarbazone (TSC 12)

Section 2: Enzyme preparation

Tyrosinase was isolated and purified as described in [48-50 references in the manuscript]. Briefly, fresh mushrooms – *Agaricus bisporus* (local supplier in Wrocław, 300 g) were cut into small pieces, mixed with cold acetone (550 ml) and homogenized (3 min.). The suspension was extracted on magnetic stirrer for 40 min.. Then the mixture was centrifuged (20 min., 8000 rpm). The pellet was suspended in phosphate buffer (200 ml, 0.1 M, pH 7.0), containing 2 g of polyvinyl pyrrolidone (Sigma-Aldrich). Remaining acetone was removed with vacuum pump. The suspension was centrifuged (20 min., 8000 rpm). Ammonium sulphate was added to the supernatant to 30% saturation. Salting out was performed using magnetic stirrer (30 min., 4 °C). Then the mixture was centrifuged (20 min., 8000 rpm). Ammonium sulphate was added to the supernatant to 60% saturation. Salting out and centrifugation were carried out the same way as described above. The pellet was dissolved in phosphate buffer (0.1 M, pH 7.0) and loaded onto the DEAE-Sepharose (Sigma-Aldrich) column to perform the gradient elution with NaCl. Collected tyrosinase fractions were salted out with ammonium sulphate to 65% saturation. Then the mixture was centrifuged (40 min., 8000 rpm) and the pellet was dissolved in 0.1 M sodium phosphate buffer containing 0.7 M ammonium sulfate. Phenyl-Sepharose (Sigma-Aldrich) column was washed with 0.7 M ammonium sulfate in phosphate buffer (0.1 M, pH 6.8) and the enzyme was eluted with 0.2 M ammonium sulfate in the same buffer. Collected active tyrosinase fractions were salted out with ammonium sulphate to 65% saturation. Then the mixture was centrifuged (40 min., 8000 rpm) and the he pellet was dissolved in 0.1 M sodium phosphate buffer. The enzyme was then desalted on a Sephadex G-25 (Sigma-Aldrich, Taufkirchen, Germany). At each step the tyrosinase activity was monitored with 1 mM L-Dopa. Protein concentration was measured according to Bradford's method using bovine serum albumin (Sigma-Aldrich, Taufkirchen, Germany) as a standard. Protein purity was monitored with SDS-polyacrylamide gel electrophoresis. Specific activity of the purified enzyme was 8376 U/mg.
